# Synthesis of Benzofuro[3,2-*b*]indol-3-one Derivatives via Dearomative (3 + 2) Cycloaddition of 2-Nitrobenzofurans and *para*-Quinamines

**DOI:** 10.3390/molecules29051163

**Published:** 2024-03-05

**Authors:** Wei-Cheng Yuan, Hai-Ying Zeng, Yan-Ping Zhang, Jian-Qiang Zhao, Yong You, Jun-Qing Yin, Ming-Qiang Zhou, Zhen-Hua Wang

**Affiliations:** 1 Innovation Research Center of Chiral Drugs, Institute for Advanced Study, Chengdu University, Chengdu 610106, Chinazhangyanping@cdu.edu.cn (Y.-P.Z.); zhaojianqiang@cdu.edu.cn (J.-Q.Z.); youyong@cdu.edu.cn (Y.Y.); yinjunqing@cdu.edu.cn (J.-Q.Y.); 2 China National Engineering Research Center of Chiral Drugs, Chengdu Institute of Organic Chemistry, Chinese Academy of Sciences, Chengdu 610041, China

**Keywords:** dearomatization, *para*-quinamines, 2-nitrobenzofurans, cycloaddition reactions, heterocycles

## Abstract

An efficient dearomative (3 + 2) cycloaddition of *para*-quinamines and 2-nitrobenzofurans has been developed. This reaction proceeds smoothly under mild conditions and affords a series of benzofuro[3,2-*b*]indol-3-one derivatives in good to excellent yields (up to 98%) with perfect diastereoselectivities (all cases > 20:1 *dr*). The scale-up synthesis and versatile derivatizations demonstrate the potential synthetic application of the protocol. A plausible reaction mechanism is also proposed to account for the observed reaction process. This work represents the first instance of the *N*-triggered dearomative (3 + 2) cycloaddition of 2-nitrobenzofurans.

## 1. Introduction

**Figure 1:**
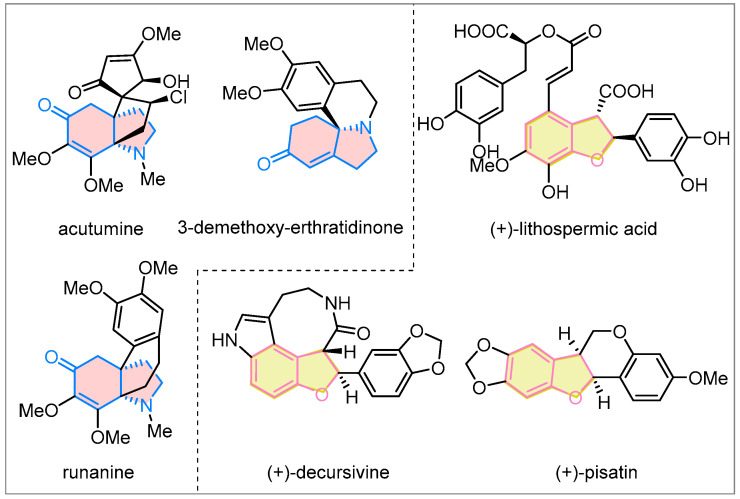
Natural products containing hydroindoline-5-one or 2,3-dihydrobenzofuran cores.

Polycyclic frameworks are not only commonly found in biologically active molecules and natural products [1,2,3,4,5], but they also have the potential to provide structural foundations for fragment-based drug discovery [6,7,8,9,10]. The ring-fusion strategy has been one of the most powerful methods for efficiently constructing polycyclic frameworks [11,12,13,14,15,16]. Hydroindoline-5-one and 2,3-dihydrobenzofuran are abundant bioactive core structures (Figure 1) [17,18,19,20,21,22]. In fact, most natural products and their analogs containing hydroindoline-5-one and 2,3-dihydrobenzofuran cores exhibit promising biological properties, such as selective T-cell cytotoxicity and antimalarial, anti-HIV, and antifungal activity [20,23,24,25]. In this context, developing effective and innovative approaches for fusing these two frameworks to construct structurally diverse polycyclic compounds is appealing and highly desirable.

**Scheme 1:**
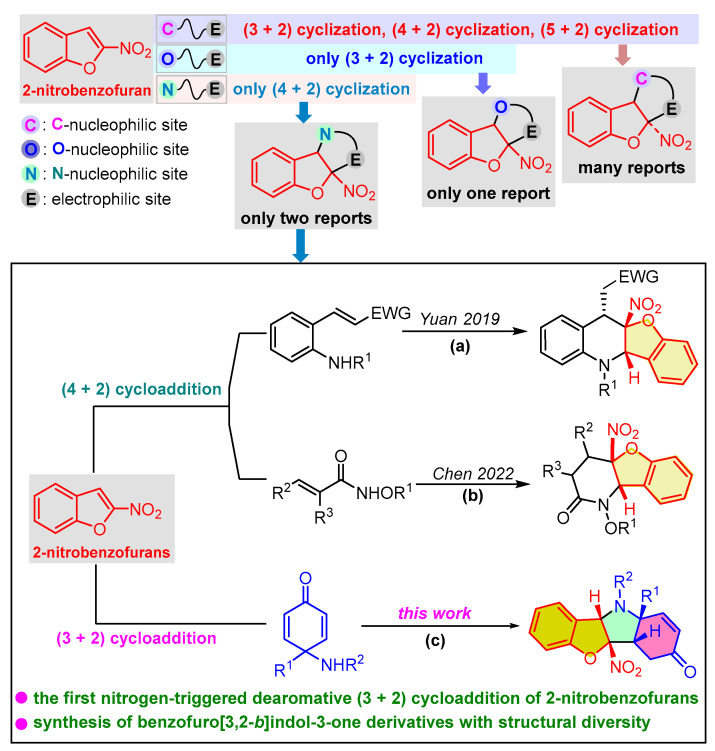
Profile of dearomative cyclization reactions of 2-nitrobenzofurans [38,39].

In recent years, extensive research on the dearomative cyclization reaction of 2-nitrobenzofuran has revealed the significant potential of this strategy in the construction of polycyclic compounds containing a 2,3-dihydrobenzofuran core (Scheme 1, top) [26,27,28]. Among the numerous transformations with respect to the dearomative cyclization reaction of 2-nitrobenzofurans, the carbon nucleophile-triggered dearomative (3 + 2), (4 + 2), and (5 + 2) cyclization reactions have been extensively studied [29,30,31,32,33,34,35,36]. In contrast, there have been limited reports on oxygen or nitrogen nucleophile-triggered dearomative cyclization reactions so far. You and co-workers reported on the only reaction of oxygen nucleophile-triggered dearomative (3 + 2) cyclization between 2-nitrobenzofurans and epoxybutenes for the straightforward construction of tetrahydrofurobenzofurans with a chiral palladium catalyst [37]. In addition, there are two reports so far on dearomative cyclization reactions of 2-nitrobenzofurans triggered by nitrogen nucleophiles [38,39]. One is the asymmetric dearomative (4 + 2) cycloaddition reaction reported by our group, involving 2-nitrobenzofurans and 2-aminochalcones to construct tetrahydrobenzofuro[3,2-*b*]quinolines by using a chiral squaramide catalyst (Scheme 1a) [38]. The other is the base-catalyzed (4 + 2) cycloaddition reaction between 2-nitrobenzofurans and *N*-alkoxyacrylamides to access [3,2-*b*]benzofuropyridinones (Scheme 1b) [39]. These precedents have demonstrated the great potential of heteroatomic nucleophile-driven dearomative cyclization reaction of 2-nitrobenzofurans in the construction of complex polyheterocyclic compounds. On the other hand, much attention has been paid to the application of *para*-quinamines as three-atom building blocks for the cascade reaction to construct nitrogen-containing heterocycles with structural diversity [40,41,42,43,44,45,46,47,48]. *Para*-quinamines are often used as *N*-nucleophiles to undergo (3 + 2) cycloaddition with two-atom reaction partners, thus leading to the formation of hydroindoline-5-one scaffolds [49,50,51,52,53]. Therefore, we speculated that conducting the dearomative (3 + 2) cycloaddition reaction between *para*-quinamines and 2-nitrobenzofurans would lead to the fusion of hydroindoline-5-one and 2,3-dihydrobenzofuran. As part of our ongoing research on the dearomatization of electron-deficient heteroarenes [54,55,56,57], herein, we will report the first nitrogen nucleophile-triggered dearomative (3 + 2) cycloaddition reaction of 2-nitrobenzofurans by using *para*-quinamines to construct benzofuro[3,2-*b*]indol-3-one skeletons (Scheme 1c).

## 2. Results and Discussion

In the early stage of the experiment, we chose potassium *tert*-butoxide (*^t^*BuOK) as the base to verify the possibility of the (3 + 2) cycloaddition reaction between *para*-quinamine **1a** and 2-nitrobenzofuran **2a** at 65 °C with acetonitrile as the solvent. To our delight, we found that the reaction proceeded smoothly under these conditions and produced the cycloadduct **3aa** in 51% isolated yield with >20:1 *dr* value (Table 1, entry 1). With this promising result in hand, we continued our investigations on other types of bases, and the outcomes indicated that inorganic bases have an advantage over organic bases for the (3 + 2) cycloaddition transformation (Table 1, entries 2–4, 7, and 8 vs. 5 and 6). Among them, K_2_CO_3_ serving as a base could produce a better result (Table 1, entry 4). Afterward, we further examined the influence of the solvent on the reaction. When acetonitrile was changed to ethyl acetate, tetrahydrofuran, methyl tertiary butyl ether, alcohol, acetone, or ethyl acetate, the reaction did not show a positive influence on improving the yield of **3aa** (Table 1, entries 9–15). Subsequently, the ratio of the starting materials **1a** to **2a** was also thoroughly investigated (Table 1, entries 16 and 17). Adjusting the ratio of **1a**/**2a** from 1:1.2 to 1.2:1 could elevate the yield of product **3aa** to 88% (Table 1, entry 16). Moreover, by changing the ratio of **1a**/**2a** to 1.5:1, the dearomative (3 + 2) cycloaddition reaction could proceed smoothly and furnish product **3aa** at a 95% yield (Table 1, entry 17). Conducting the reaction at 80 °C did not lead to an increase in the yield of **3aa** (Table 1, entry 18). Delightfully, reducing the amount of K_2_CO_3_ from 2.0 equiv to 1.0 equiv had no negative impact on the yield of product **3aa** (Table 1, entry 19). We also examined strong bases, including NaH and LiHMDS, and found that the reaction time was significantly reduced. However, the isolated yield of product **3aa** did not show any further improvement.

**Scheme 2:**
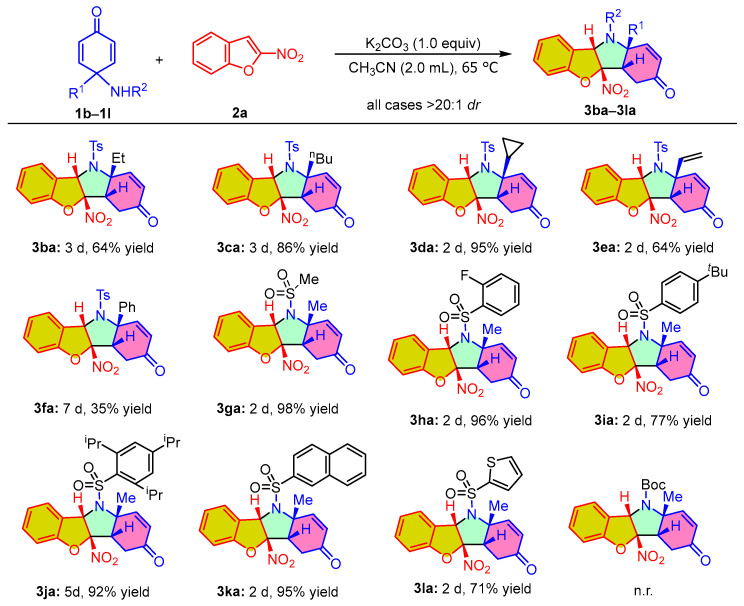
Scope study on the dearomative (3 + 2) cycloaddition reaction of various *para*-quinamines **1** with 2-nitrobenzofuran **2a**. Reaction conditions: unless otherwise noted, the reactions were carried out with **1** (0.15 mmol), **2a** (0.10 mmol), and K_2_CO_3_ (1.0 equiv) in 2.0 mL of acetonitrile at 65 °C for the specified reaction time. The *dr* values were determined by ^1^H NMR analysis. The yields refer to the isolated yield of product. n.r. = no reaction.

After successfully obtaining the optimal conditions of the dearomative (3 + 2) cycloaddition reaction, we next investigated the substrate adaptability of this protocol by using various *para*-quinamines **1** to react with 2-nitrobenzofuran **2a**. As shown in Scheme 2, the reactions showed good adaptability with *para*-quinamines and furnished a series of benzofuro[3,2-*b*]indol-3-one derivatives in excellent diastereoselectivities (all cases > 20:1 *dr*). Substrates **1b**–**1d** equipped with a linear or cyclic alkyl group (Et, *^t^*Bu, or cyclopropyl) could smoothly react with **2a** under the standard conditions, producing the corresponding cycloadducts **3ba**–**3da** in good to high yields. *Para*-quinamine **1e** bearing a vinyl substituent also could smoothly react with 2-nitrobenzofuran **2a** and produce product **3ea** at 64% yield. However, when installing a phenyl group into the *para*-quinamine, the reactivity of the reaction significantly decreased. Even extending the reaction time to seven days, the corresponding product **3fa** could be obtained only at 35% yield. On the other hand, we also investigated the influence of the substituents attached to the nitrogen atom of *para*-quinamine on the dearomative (3 + 2) cycloaddition reaction. It was found that the methanesulfonyl-substituted *para*-quinamine was compatible with the developed cycloaddition reaction, leading to the formation of product **3ga** at 98% yield. In addition, we also found that the developed protocol exhibited tolerance towards *para*-quinamine **1h**–**1j** bearing various substituted phenylsulfonyl groups, allowing for the successful synthesis of the desired cycloadducts **3ha**–**3ja** at 77–96% yield. Moreover, introducing the naphthalenesulfonyl group or heteroaromatic cyclic sulfonyl group into *para*-quinamines did not significantly affect the dearomative (3 + 2) cycloaddition reaction, as exemplified by the formation of products **3ka** and **3la** at 95% and 71% yield, respectively. When we replaced the sulfonyl substituent with the Boc group, the corresponding dearomative (3 + 2) cycloaddition reaction did not occur under the standard conditions, which indicated that the proton attached to the nitrogen is not sufficiently acidic to be deprotonated by K_2_CO_3_.

**Scheme 3:**
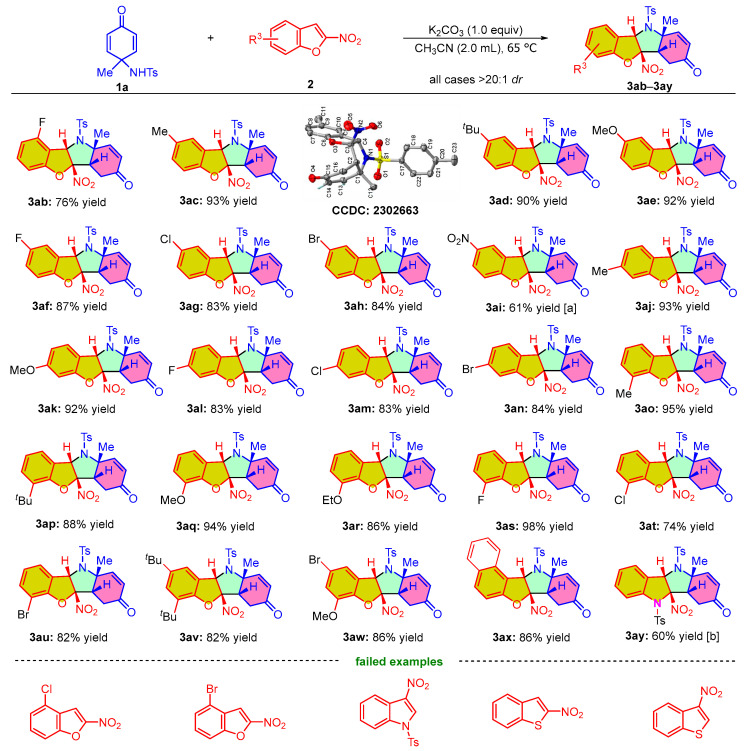
Substrate scope on the dearomative (3 + 2) cycloaddition reaction of various 2-nitrobenzofurans **2** with *para*-quinamines **1a**. Reaction conditions: unless otherwise noted, the reactions were carried out with **1a** (0.15 mmol), **2** (0.10 mmol), and K_2_CO_3_ (1.0 equiv) in 2.0 mL of acetonitrile at 65 °C for 2 days. The *dr* values were determined by ^1^H NMR analysis. The yields refer to the isolated yield of product. [a] The reaction was carried out for 3 days. [b] The reaction was carried out with **1a** (0.15 mmol), **2y** (0.10 mmol), and Cs_2_CO_3_ (1.0 equiv) in 2.0 mL of acetonitrile at 25 °C for 12 h.

The scope of substrates with respect to 2-nitrobenzofurans **2** for the developed protocol was next tested. As shown in Scheme 3, all the reactions that occurred were able to produce polyheterocyclic compounds with excellent diastereoselectivities (>20:1 *dr*). 4-fluoro-2-nitrobenzofuran **1b** could smoothly react with *para*-quinamine **2a** to produce the corresponding product **3ab** at 76% yield. However, when the fluorine atom was replaced with a bromine or chlorine atom, perhaps due to the increasing steric hindrance limiting the transformation, the reactions with the substrates did not occur under standard conditions. We also examined the different substituents at C5-position of the 2-nitrobenzofurans and found that substrates **2c**–**2e** with electron-donating groups (Me-, *^t^*Bu-, MeO-) exhibited better performance for providing the corresponding products **3ac**–**3ae** at 90–93% yield. In contrast, 2-nitrobenzofurans **2f**–**2i** bearing an electron-withdrawing substituent (F-, Cl-, Br-, NO_2_-) at C5-position could furnish the cycloaddition products **3af**–**3ai** at 61–87% isolated yield. As to the reaction of the C6-substituted 2-nitrobenzofurans with **1a**, regardless of the electron-donating (Me-, MeO-) or electron-withdrawing substitution (F-, Cl-, Br-), it was found that the transformations proceeded smoothly and generated their respective cycloadducts **3aj**–**3an** at 83–93% yield. Likewise, upon incorporating an electron-donating (Me-, *^t^*Bu-, MeO-, EtO-) or electron-withdrawing substituent (F-, Cl-, Br-) at C7-position, the corresponding 2-nitrobenzofurans **2ao**–**2au** were also applicable to the dearomative (3 + 2) cycloaddition reaction, affording cyclicadducts **3ao**–**3au** at 74–98% yield. Moreover, the reactions between 2-nitrobenzofurans bearing two substituents and *para*-quinamine **1a** proceeded smoothly and gave rise to the desired products **3av** and **3aw** at 82% and 86% yield, respectively. In addition, 2-nitronaphtho[2,1-*b*]furan **2x** was added to the (3 + 2) cycloaddition reaction to generate product **3ax** at 86% yield. However, the dearomative (3 + 2) cycloaddition reaction of 2-nitroindole **2y** and **1a** required Cs_2_CO_3_ as the base to initiate the reaction, delivering the corresponding product **3ay** at 60% yield. Unfortunately, the dearomative (3 + 2) cycloaddition reactions with 3-nitroindole and 2- and 3-nitrobenzo[b]thiophene as substrates did not proceed under the developed conditions. Ultimately, we performed single crystal growth and X-ray diffraction analysis to verify the structure and relative configuration of product **3ac** (CCDC 2302663 contains the supplementary crystallographic data for this paper. For details, see the Supporting Information).

**Scheme 4:**
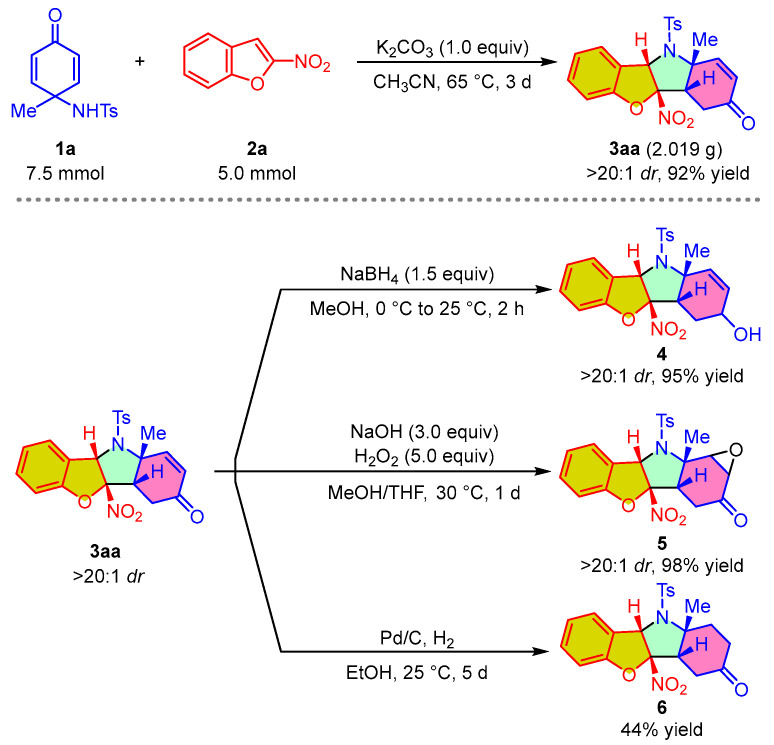
Scale-up synthesis and synthetic transformations of **3aa**.

To demonstrate the practicality and scalability of this methodology, we performed scale-up preparation and diverse transformations of product **3aa**. As shown in Scheme 4, the dearomative (3 + 2) cycloaddition reaction of **1a** and **2a** was performed on a preparative scale of 5.0 mmol under standard reaction conditions to generate product **3aa** at 92% isolated yield with >20:1 *dr*. The treatment of compound **3aa** with sodium borohydride in methanol at room temperature resulted in the selective reduction of the carbonyl group in **3aa**, leading to the formation of product **4** at 95% yield with >20:1 *dr*. In addition, treating **3aa** with H_2_O_2_ as an oxidizing agent under alkaline conditions led to the efficient epoxidation of the double bond, affording product **5** at 95% yield with no loss of diastereoselectivity. The conversion of compound **3aa** into **6** was achieved with the combination of Pd/C and H_2_ in ethyl alcohol at room temperature, but only a moderate yield.

**Scheme 5:**
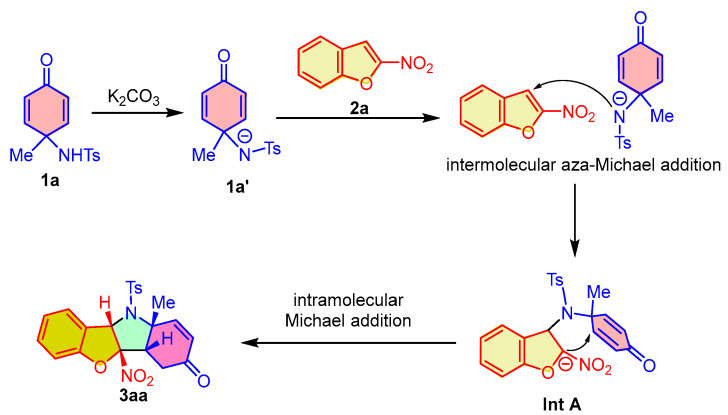
Reaction mechanism for the dearomative (3 + 2) cycloaddition of *para*-quinamines and 2-nitrobenzofurans.

In order to further provide a more comprehensive explanation for the dearomative (3 + 2) cycloaddition reaction, we propose a possible reaction mechanism according to the experimental results. As outlined in Scheme 5, using the reaction between *para*-quinamine **1a** and 2-nitrobenzofuran **2a** as an example, under the promotion of an inorganic base, the in situ-generated intermediate **1a′** from **1a** undergoes an intermolecular aza-Michael reaction by attacking the C3-position of 2-nitrobenzofuran **2a**, which leads to the dearomatization of 2-nitrobenzofuran and the formation of intermediate **Int A**. Subsequently, an intramolecular Michael addition of intermediate **Int A** occurs and gives rise to the fusion of hydroindoline-5-one and 2,3-dihydrobenzofuran, thus producing the polycyclic benzofuro[3,2-*b*]indol-3-one **3aa**.

## 3. Materials and Methods

### 3.1. General Information

Reagents were purchased from commercial sources and were used as received unless mentioned otherwise. 2-nitrobenzofurans were prepared according to the procedure described by Ackermann and co-workers [58]. *Para*-quinamines were prepared according to the procedure described by Ackermann and co-workers [45,59]. Flash column chromatography was performed over silica gel (H, purchased from Qingdao Ocean Chemical Co., Ltd. Qingdao, China). Analytical thin-layer chromatography (TLC) was performed on silica gel HSGF254 glass plates (purchased from Yantai Xinuo Chemical Co., Ltd. Yantai, China) containing a 254 nm fluorescent indicator. Proton nuclear magnetic resonance (^1^H NMR) spectra were measured on a Bruker AVANCE NEO 400 MHz spectrometer (Billerica, MA, USA) in ambient temperature at 400 MHz. Proton chemical shifts are reported in parts per million (*δ* scale) and are referenced using tetramethylsilane (TMS) as an internal standard or residual protium in the NMR solvent (CDCl_3_: δ 7.26 (CHCl_3_) or DMSO-*d*_6_: *δ* 2.50 (CD_2_HSOCD_3_)). Data are reported as follows: chemical shift [multiplicity (s = singlet, d = doublet, t = triplet, q = quartet, m = multiplet, dd = doublet of doublets, td = triplet of doublets, brs = broad singlet), coupling constant(s) (Hz), integration]. Carbon-13 nuclear magnetic resonance (^13^C NMR) spectra were also measured on a Bruker AVANCE NEO 400 MHz spectrometer in ambient temperature of ^13^C at 101MHz. Carbon chemical shifts are reported in parts per million (*δ* scale), and referenced using the carbon resonances of the solvent (*δ* 77.16 (CDCl_3_) or *δ* 39.52 (DMSO-*d*_6_)). ^1^H NMR and ^13^C NMR spectra were recorded in CDCl_3_ or DMSO-*d*_6_. ^1^H NMR chemical shifts are reported in ppm employed as the internal standard (CDCl_3_ at 7.26 ppm, DMSO-*d*_6_ at 2.50 ppm). The melting points of products were recorded on a Büchi Melting Point B-545 and temperatures were not corrected. High-resolution mass spectra (HRMS) were recorded by an Agilent 6545 LC/Q-TOF mass spectrometer by using an electrospray ionization (ESI) source analyzed by quadrupole time-of-flight (Q-TOF). Mass error of HRMS data is maintained below 5 ppm.

### 3.2. General Experimental Procedures for Synthesis of Compounds ***3*** (Scheme 2 and Scheme 3)

*10a-Methyl-4b-nitro-10-tosyl-4,4a,4b,9b,10,10a-hexahydro-3H-benzofuro[3,2-b]indol-3-one* (**3aa**): white solid; 41.6 mg, 95% yield; m.p. 203.1–204.0 °C; ^1^H NMR (400 MHz, DMSO-*d*_6_) *δ* 7.96 (d, *J* = 8.3 Hz, 2H), 7.73 (d, *J* = 7.5 Hz, 1H), 7.49 (d, *J* = 8.2 Hz, 2H), 7.38 (m, 1H), 7.16 (m, 1H), 6.92 (d, *J* = 8.1 Hz, 1H), 6.58 (s, 1H), 6.38 (d, *J* = 11.7 Hz, 1H), 5.50 (d, *J* = 10.3 Hz, 1H), 3.59 (d, *J* = 6.0 Hz, 1H), 2.88 (dd, *J* = 18.1, 6.2 Hz, 1H), 2.58 (d, *J* = 18.1 Hz, 1H), 2.44 (s, 3H), 1.44 (s, 3H); ^13^C{^1^H} NMR (101 MHz, DMSO-*d*_6_) *δ* 193.3, 158.1, 148.3, 143.9, 140.1, 131.5, 130.1, 127.6, 127.3, 126.7, 125.1, 123.9, 119.8, 110.1, 71.0, 64.5, 52.6, 32.8, 22.2, 21.0; HRMS (ESI) *m*/*z* [M + H]^+^ Calcd for C_22_H_21_N_2_O_6_S: 441.1115; found 441.1116.

*10a-Ethyl-4b-nitro-10-tosyl-4,4a,4b,9b,10,10a-hexahydro-3H-benzofuro[3,2-b]indol-3-one* (**3ba**): white solid; 29.1 mg, 64% yield; m.p. 192.5–193.2 °C; ^1^H NMR (400 MHz, CDCl_3_) *δ* 7.93 (d, *J* = 7.6 Hz, 1H), 7.85 (d, *J* = 7.9 Hz, 2H), 7.38 (d, *J* = 7.9 Hz, 2H), 7.33 (m, 1H), 7.11 (m, 1H), 6.87 (d, *J* = 8.2 Hz, 1H), 6.56 (d, *J* = 10.5 Hz, 1H), 6.24 (s, 1H), 5.67 (d, *J* = 10.5 Hz, 1H), 3.55 (d, *J* = 6.2 Hz, 1H), 2.87 (d, *J* = 18.3 Hz, 1H), 2.57 (dd, *J* = 18.3, 6.4 Hz, 1H), 2.48 (s, 3H), 2.06 (m, 1H), 1.85 (m, 1H), 0.69 (t, *J* = 7.4 Hz, 3H); ^13^C{^1^H} NMR (101 MHz, CDCl_3_) δ 193.2, 158.6, 147.4, 144.4, 140.4, 131.8, 130.2, 129.2, 128.7, 127.1, 124.5, 124.3, 119.8, 110.5, 72.4, 68.4, 51.3, 34.2, 29.0, 21.8, 8.9; HRMS (ESI) *m*/*z*: [M + Na]^+^ Calcd for C_23_H_22_N_2_O_6_SNa: 477.1091, found 477.1064.

*10a-Butyl-4b-nitro-10-tosyl-4,4a,4b,9b,10,10a-hexahydro-3H-benzofuro[3,2-b]indol-3-one* (**3ca**): white solid; 41.6 mg, 86% yield; m.p. 182.8–183.6 °C; ^1^H NMR (400 MHz, CDCl_3_) *δ* 7.93 (d, *J* = 7.6 Hz, 1H), 7.85 (d, *J* = 7.9 Hz, 2H), 7.38 (d, *J* = 7.9 Hz, 2H), 7.33 (m, 1H), 7.11 (m, 1H), 6.87 (d, *J* = 8.2 Hz, 1H), 6.56 (d, *J* = 10.5 Hz, 1H), 6.24 (s, 1H), 5.67 (d, *J* = 10.5 Hz, 1H), 3.55 (d, *J* = 6.2 Hz, 1H), 2.87 (d, *J* = 18.3 Hz, 1H), 2.57 (dd, *J* = 18.3, 6.4 Hz, 1H), 2.48 (s, 3H), 2.06 (m, 1H), 1.85 (m, 1H), 0.69 (t, *J* = 7.4 Hz, 3H); ^13^C{^1^H} NMR (101 MHz, CDCl_3_) *δ* 193.2, 158.5, 147.6, 144.4, 140.3, 131.8, 130.1, 128.8, 128.7, 127.1, 124.5, 124.3, 119.7, 110.4, 72.3, 68.0, 51.5, 36.0, 34.1, 26.6, 22.9, 21.7, 13.6; HRMS (ESI) *m*/*z*: [M + H]^+^ Calcd for C_25_H_27_N_2_O_6_S: 483.1584, found 483.1587.

*10a-Cyclopropyl-4b-nitro-10-tosyl-4,4a,4b,9b,10,10a-hexahydro-3H-benzofuro[3,2-b]indol-3-one* (**3da**): white solid; 44.2 mg, 95% yield; m.p. 231.5–232.3 °C; ^1^H NMR (400 MHz, CDCl_3_) *δ* 7.92 (m, 3H), 7.34 (m, 3H), 7.12 (m, 1H), 6.86 (d, *J* = 8.1 Hz, 1H), 6.32 (s, 1H), 6.28 (d, *J* = 10.5 Hz, 1H), 5.67 (d, *J* = 10.5 Hz, 1H), 3.65 (d, *J* = 5.8 Hz, 1H), 2.82 (d, *J* = 18.1 Hz, 1H), 2.66 (dd, *J* = 18.2, 6.2 Hz, 1H), 2.46 (s, 3H), 0.58 (t, *J* = 6.9 Hz, 2H), 0.48 (m, 1H), 0.28 (m, 1H), 0.17–0.09 (m, 1H); ^13^C{^1^H} NMR (101 MHz, CDCl_3_) *δ* 193.3, 158.4, 144.3, 142.3, 140.5, 131.8, 131.6, 129.9, 128.4, 127.1, 124.7, 124.3, 119.6, 110.4, 72.5, 67.8, 55.3, 33.6, 21.7, 16.5, 3.4, 2.1; HRMS (ESI) *m*/*z*: [M + H]^+^ Calcd for C_24_H_23_N_2_O_6_S: 467.1271, found 467.1272.

*4b-Nitro-10-tosyl-10a-vinyl-4,4a,4b,9b,10,10a-hexahydro-3H-benzofuro[3,2-b]indol-3-one* (**3ea**): white solid; 29.0 mg, 64% yield; m.p. 194.9–195.7 °C; ^1^H NMR (400 MHz, CDCl_3_) *δ* 7.93 (d, *J* = 7.6 Hz, 1H), 7.83 (d, *J* = 7.9 Hz, 2H), 7.36 (m, 3H), 7.14 (m, 1H), 6.89 (d, *J* = 8.2 Hz, 1H), 6.55 (d, *J* = 10.4 Hz, 1H), 6.25 (s, 1H), 5.79 (d, *J* = 10.4 Hz, 1H), 5.52 (dd, *J* = 17.2, 10.4 Hz, 1H), 5.30 (d, *J* = 10.4 Hz, 1H), 5.20 (d, *J* = 17.3 Hz, 1H), 3.51 (d, *J* = 5.4 Hz, 1H), 2.76 (d, *J* = 17.8 Hz, 1H), 2.48 (s, 3H), 2.43 (dd, *J* = 17.9, 6.0 Hz, 1H); ^13^C{^1^H} NMR (101 MHz, CDCl_3_) *δ* 193.2, 158.5, 144.5, 144.3, 139.7, 134.3, 131.9, 130.5, 130.0, 128.5, 127.5, 124.4, 124.3, 121.3, 119.6, 110.6, 72.0, 68.1, 53.1, 32.3, 21.8; HRMS (ESI) *m*/*z*: [M + H]^+^ Calcd for C_23_H_21_N_2_O_6_S: 453.1115, found 453.1116.

*4b-Nitro-10a-phenyl-10-tosyl-4,4a,4b,9b,10,10a-hexahydro-3H-benzofuro[3,2-b]indol-3-one* (**3fa**): white solid; 17.7 mg, 35% yield; m.p. 245.3–245.8 °C; ^1^H NMR (400 MHz, CDCl_3_) *δ* 8.05 (d, *J* = 7.6 Hz, 1H), 7.37 (m, 1H), 7.28 (d, *J* = 10.9 Hz, 1H), 7.26–7.07 (m, 5H), 6.98 (m, 6H), 6.36 (s, 1H), 5.86 (d, *J* = 10.5 Hz, 1H), 4.08 (d, *J* = 4.4 Hz, 1H), 2.73 (d, *J* = 17.8 Hz, 1H), 2.40 (s, 3H), 2.19 (dd, *J* = 17.8, 5.7 Hz, 1H); ^13^C{^1^H} NMR (101 MHz, CDCl_3_) *δ* 193.2, 158.9, 146.1, 143.6, 138.9, 133.8, 131.9, 130.1, 129.4, 129.3, 129.0, 128.1, 127.2, 124.3, 124.3, 118.9, 110.4, 73.2, 68.5, 56.6, 32.6, 21.6; HRMS (ESI) *m*/*z*: [M + H]^+^ Calcd for C_27_H_23_N_2_O_6_S: 503.1271, found 503.1275.

*10a-Methyl-10-(methylsulfonyl)-4b-nitro-4,4a,4b,9b,10,10a-hexahydro-3H-benzofuro[3,2-b]indol-3-one* (**3ga**): white solid; 35.8 mg, 98% yield; m.p. 186.1–186.5 °C; ^1^H NMR (400 MHz, CDCl_3_) *δ* 7.75 (d, *J* = 7.6 Hz, 1H), 7.32 (m, 1H), 7.09 (m, 1H), 6.86 (d, *J* = 8.2 Hz, 1H), 6.47 (d, *J* = 10.4 Hz, 1H), 5.94 (s, 1H), 5.59 (d, *J* = 10.4 Hz, 1H), 3.61 (d, *J* = 5.7 Hz, 1H), 3.26 (s, 3H), 2.92 (d, *J* = 18.0 Hz, 1H), 2.71 (dd, *J* = 18.0, 6.1 Hz, 1H), 1.93 (s, 3H); ^13^C{^1^H} NMR (101 MHz, CDCl_3_) *δ* 192.5, 158.4, 148.3, 131.9, 128.1, 128.0, 124.3, 123.7, 119.8, 110.6, 71.3, 64.8, 54.1, 44.5, 33.1, 23.9; HRMS (ESI) *m*/*z*: [M + H]^+^ Calcd for C_16_H_17_N_2_O_6_S: 365.0802, found 365.0806.

*10-((2-Fluorophenyl)sulfonyl)-10a-methyl-4b-nitro-4,4a,4b,9b,10,10a-hexahydro-3H-benzofuro[3,2-b]indol-3-one* (**3ha**): white solid; 42.8 mg, 96% yield; m.p. 224.9–225.6 °C; ^1^H NMR (400 MHz, CDCl_3_) *δ* 8.00 (m, 1H), 7.88 (d, *J* = 7.6 Hz, 1H), 7.68 (m, 1H), 7.40–7.30 (m, 3H), 7.13 (m, 1H), 6.87 (d, *J* = 8.2 Hz, 1H), 6.61 (d, *J* = 10.4 Hz, 1H), 6.34 (s, 1H), 5.61 (d, *J* = 10.4 Hz, 1H), 3.57 (d, *J* = 5.8 Hz, 1H), 2.89 (d, *J* = 18.1 Hz, 1H), 2.60 (dd, *J* = 18.1, 6.2 Hz, 1H), 1.47 (s, 3H); ^13^C{^1^H} NMR (101 MHz, CDCl_3_) *δ* 192.7, 158.9 (d, *J_CF_* = 255.4 Hz), 158.6, 148.2, 135.8 (d, *J_CF_* = 8.7 Hz), 131.9, 130.9 (d, *J_CF_* = 13.5 Hz), 129.9, 128.2 (d, *J_CF_* = 29.3 Hz), 125.0 (d, *J_CF_* = 3.7 Hz), 124.3, 124.3, 119.6, 117.6 (d, *J_CF_* = 21.4 Hz), 110.5, 71.2 (d, *J_CF_* = 7.2 Hz), 64.6, 52.8, 32.9, 23.3; HRMS(ESI) *m*/*z*: [M + H]^+^ Calcd for C_21_H_18_FN_2_O_6_S:445.0864, found 445.0865.

*10-((4-(tert-Butyl)phenyl)sulfonyl)-10a-methyl-4b-nitro-4,4a,4b,9b,10,10a-hexahydro-3H-benzofuro[3,2-b]indol-3-one* (**3ia**): white solid; 37.3 mg, 77% yield; m.p. 239.7–240.5 °C; ^1^H NMR (400 MHz, CDCl_3_) *δ* 7.90 (m, 3H), 7.60 (d, *J* = 8.1 Hz, 2H), 7.34 (m, 1H), 7.13 (m, 1H), 6.87 (d, *J* = 8.2 Hz, 1H), 6.54 (d, *J* = 10.4 Hz, 1H), 6.27 (s, 1H), 5.56 (d, *J* = 10.4 Hz, 1H), 3.45 (d, *J* = 5.6 Hz, 1H), 2.86 (d, *J* = 18.1 Hz, 1H), 2.58 (dd, *J* = 18.1, 6.1 Hz, 1H), 1.50 (s, 3H), 1.38 (s, 9H); ^13^C{^1^H} NMR (101 MHz, CDCl_3_) δ 192.7, 158.4, 157.3, 148.6, 140.2, 131.8, 128.2, 127.8, 126.6, 126.6, 124.3, 124.3, 119.8, 110.5, 72.3, 64.5, 53.8, 35.4, 32.9, 31.2, 23.0; HRMS (ESI) *m*/*z*: [M + H]^+^ Calcd for C_25_H_27_N_2_O_6_S: 483.1584, found 483.1591.

*10a-Methyl-4b-nitro-10-((2,4,6-triisopropylphenyl)sulfonyl)-4,4a,4b,9b,10,10a-hexahydro-3H-benzofuro[3,2-b]indol-3-one* (**3ja**): white solid; 51.0 mg, 92% yield; m.p. 204.7–205.6 °C; ^1^H NMR (400 MHz, CDCl_3_) *δ* 7.91 (d, *J* = 7.6 Hz, 1H), 7.32 (m, 1H), 7.24 (s, 2H), 7.10 (m, 1H), 6.99 (d, *J* = 10.5 Hz, 1H), 6.83 (d, *J* = 8.2 Hz, 1H), 6.24 (s, 1H), 5.59 (d, *J* = 10.5 Hz, 1H), 4.16 (m, 2H), 3.44 (d, *J* = 5.5 Hz, 1H), 2.93 (dd, *J* = 15.9, 9.1 Hz, 2H), 2.61 (dd, *J* = 18.2, 6.0 Hz, 1H), 1.32 (d, *J* = 6.7 Hz, 12H), 1.28 (d, *J* = 6.9 Hz, 6H), 1.24 (s, 3H); ^13^C{^1^H} NMR (101 MHz, CDCl_3_) *δ* 193.0, 158.7, 154.3, 151.0, 147.5, 134.2, 131.7, 128.6, 128.5, 124.4, 124.1, 123.7, 119.2, 110.3, 71.1, 64.1, 53.1, 34.2, 32.8, 29.6, 25.2, 24.4, 23.9, 23.6, 23.5; HRMS (ESI) *m*/*z*: [M + H]^+^ Calcd for C_30_H_37_N_2_O_6_S: 553.2367, found 553.2369.

*10a-Methyl-10-(naphthalen-2-ylsulfonyl)-4b-nitro-4,4a,4b,9b,10,10a-hexahydro-3H-benzofuro[3,2-b]indol-3-one* (**3ka**): white solid; 45.4 mg, 95% yield; m.p. 167.9–168.5 °C; ^1^H NMR (400 MHz, CDCl_3_) *δ* 8.57 (s, 1H), 8.07 (d, *J* = 8.7 Hz, 1H), 8.02 (d, *J* = 7.8 Hz, 1H), 7.95 (m, 3H), 7.69 (m, 2H), 7.36 (m, 1H), 7.16 (m, 1H), 6.89 (d, *J* = 8.2 Hz, 1H), 6.58 (d, *J* = 10.4 Hz, 1H), 6.38 (s, 1H), 5.57 (d, *J* = 10.4 Hz, 1H), 3.43 (d, *J* = 5.7 Hz, 1H), 2.86 (d, *J* = 18.1 Hz, 1H), 2.55 (dd, *J* = 18.1, 6.1 Hz, 1H), 1.48 (s, 3H); ^13^C{^1^H} NMR (101 MHz, CDCl_3_) *δ* 192.6, 158.5, 148.5, 140.0, 135.1, 132.3, 132.0, 130.2, 129.5, 129.5, 128.3, 128.3, 128.2, 128.1, 127.9, 124.5, 124.2, 121.7, 119.8, 110.6, 72.6, 64.6, 54.0, 32.9, 23.1; HRMS (ESI) *m*/*z*: [M + H]^+^ Calcd for C_25_H_21_N_2_O_6_S: 477.1115, found 477.1125.

*10a-Methyl-4b-nitro-10-(thiophen-2-ylsulfonyl)-4,4a,4b,9b,10,10a-hexahydro-3H-benzofuro[3,2-b]indol-3-one* (**3la**): white solid; 30.8 mg, 71% yield; m.p. 190.4–191.1 °C; ^1^H NMR (400 MHz, CDCl_3_) *δ* 7.83 (d, *J* = 7.6 Hz, 1H), 7.76 (d, *J* = 2.6 Hz, 1H), 7.70 (d, *J* = 4.9 Hz, 1H), 7.35 (m, 1H), 7.19–7.08 (m, 2H), 6.88 (d, *J* = 8.2 Hz, 1H), 6.45 (d, *J* = 10.3 Hz, 1H), 6.16 (s, 1H), 5.58 (d, *J* = 10.3 Hz, 1H), 3.51 (d, *J* = 5.7 Hz, 1H), 2.87 (d, *J* = 18.1 Hz, 1H), 2.63 (dd, *J* = 18.1, 6.2 Hz, 1H), 1.71 (s, 3H); ^13^C NMR (101 MHz, CDCl_3_) *δ* 192.5, 158.4, 148.3, 143.6, 132.8, 132.7, 132.0, 128.1, 127.8, 127.6, 124.3, 123.9, 119.7, 110.6, 71.6, 65.3, 53.4, 32.9, 22.7; HRMS (ESI) *m*/*z*: [M + H]^+^ Calcd for C_19_H_17_N_2_O_6_S_2_: 433.0523, found 433.0524.

*9-Fluoro-10a-methyl-4b-nitro-10-tosyl-4,4a,4b,9b,10,10a-hexahydro-3H-benzofuro[3,2-b]indol-3-one* (**3ab**): white solid; 35.0 mg, 76% yield; m.p. 207.8–208.7 °C; ^1^H NMR (400 MHz, DMSO-*d*_6_) *δ* 7.92 (d, *J* = 8.2 Hz, 2H), 7.50 (d, *J* = 8.1 Hz, 2H), 7.43 (m, 1H), 6.96 (m, 1H), 6.82 (s, 1H), 6.76 (d, *J* = 8.2 Hz, 1H), 6.31 (d, *J* = 10.4 Hz, 1H), 5.45 (d, *J* = 10.3 Hz, 1H), 3.46 (d, *J* = 5.8 Hz, 1H), 2.88 (dd, *J* = 18.2, 6.1 Hz, 1H), 2.56 (d, *J* = 18.2 Hz, 1H), 2.44 (s, 3H), 1.52 (s, 3H); ^13^C{^1^H} NMR (101 MHz, DMSO-*d*_6_) *δ* 193.1, 159.8 (d, *J_CF_* = 7.5 Hz), 159.1 (d, *J_CF_* = 255.2 Hz), 148.7, 144.1, 139.5, 133.6 (d, *J_CF_* = 8.9 Hz), 130.1, 127.4, 127.1, 120.5, 112.1 (d, *J_CF_* = 20.4 Hz), 111.1 (d, *J_CF_* = 20.6 Hz), 106.4 (d, *J_CF_* = 3.7 Hz), 69.3, 65.1, 53.1, 32.9, 22.1, 21.1; HRMS (ESI) *m*/*z*: [M + H]^+^ Calcd for C_22_H_20_FN_2_O_6_S: 459.1021, found 459.1027.

*8,10a-Dimethyl-4b-nitro-10-tosyl-4,4a,4b,9b,10,10a-hexahydro-3H-benzofuro[3,2-b]indol-3-one* (**3ac**): white solid; 42.2 mg, 93% yield; m.p. 227.7–228.6 °C; ^1^H NMR (400 MHz, DMSO-*d*_6_) *δ* 7.96 (d, *J* = 8.3 Hz, 2H), 7.53 (s, 1H), 7.49 (d, *J* = 8.2 Hz, 2H), 7.18 (d, *J* = 8.4 Hz, 1H), 6.80 (d, *J* = 8.3 Hz, 1H), 6.52 (s, 1H), 6.40 (d, *J* = 10.3 Hz, 1H), 5.51 (d, *J* = 10.3 Hz, 1H), 3.57 (d, *J* = 5.9 Hz, 1H), 2.87 (dd, *J* = 18.1, 6.1 Hz, 1H), 2.57 (d, *J* = 18.0 Hz, 1H), 2.44 (s, 3H), 2.30 (s, 3H), 1.45 (s, 3H); ^13^C{^1^H} NMR (101 MHz, DMSO-*d*_6_) *δ* 193.3, 156.2, 148.3, 143.9, 140.2, 132.9, 131.9, 130.1, 127.7, 127.3, 126.7, 125.0, 120.0, 109.7, 71.1, 64.5, 52.6, 32.8, 22.2, 21.0, 20.6; HRMS (ESI) *m*/*z*: [M + H]^+^ Calcd for C_23_H_23_N_2_O_6_S: 455.1271, found 455.1270.

*8-(tert-Butyl)-10a-methyl-4b-nitro-10-tosyl-4,4a,4b,9b,10,10a-hexahydro-3H-benzofuro[3,2-b]indol-3-one* (**3ad**): white solid; 44.8 mg, 90% yield; m.p. 228.9–229.5 °C; ^1^H NMR (400 MHz, CDCl_3_) *δ* 7.93–7.83 (m, 3H), 7.37 (m, 3H), 6.79 (d, *J* = 8.6 Hz, 1H), 6.48 (d, *J* = 10.4 Hz, 1H), 6.22 (s, 1H), 5.58 (d, *J* = 10.4 Hz, 1H), 3.42 (d, *J* = 5.5 Hz, 1H), 2.84 (d, *J* = 18.1 Hz, 1H), 2.57 (dd, *J* = 18.1, 6.1 Hz, 1H), 2.47 (s, 3H), 1.52 (s, 3H), 1.32 (s, 9H); ^13^C{^1^H} NMR (101 MHz, CDCl_3_) *δ* 192.7, 156.3, 148.5, 147.7, 144.4, 140.4, 130.2, 128.9, 127.8, 126.9, 125.0, 123.9, 120.3, 109.8, 72.4, 64.6, 53.8, 34.8, 32.9, 31.7, 23.2, 21.7; HRMS (ESI) *m*/*z*: [M + Na]^+^ Calcd for C_26_H_28_N_2_O_6_SNa: 519.1560, found 519.1568.

*8-Methoxy-10a-methyl-4b-nitro-10-tosyl-4,4a,4b,9b,10,10a-hexahydro-3H-benzofuro[3,2-b]indol-3-one* (**3ae**): white solid; 43.4 mg, 92% yield; m.p. 223.7–224.3 °C; ^1^H NMR (400 MHz, DMSO-*d*_6_) *δ* 7.96 (d, *J* = 8.2 Hz, 2H), 7.49 (d, *J* = 8.1 Hz, 2H), 7.26 (d, *J* = 2.6 Hz, 1H), 6.95 (m, 1H), 6.85 (d, *J* = 8.9 Hz, 1H), 6.53 (s, 1H), 6.43 (d, *J* = 10.4 Hz, 1H), 5.53 (d, *J* = 10.3 Hz, 1H), 3.73 (s, 3H), 3.56 (d, *J* = 5.8 Hz, 1H), 2.87 (dd, *J* = 18.1, 6.1 Hz, 1H), 2.56 (d, *J* = 18.0 Hz, 1H), 2.44 (s, 3H), 1.44 (s, 3H); ^13^C{^1^H} NMR (101 MHz, DMSO-*d*_6_) *δ* 193.7, 156.1, 152.5, 148.7, 144.4, 140.5, 130.5, 127.8, 127.1, 126.4, 120.7, 117.1, 113.1, 111.0, 71.7, 65.1, 56.1, 53.1, 33.3, 22.7, 21.5; HRMS (ESI) *m*/*z*: [M + H]^+^ Calcd for C_23_H_23_N_2_O_7_S: 471.1220, found 471.1219.

*8-Fluoro-10a-methyl-4b-nitro-10-tosyl-4,4a,4b,9b,10,10a-hexahydro-3H-benzofuro[3,2-b]indol-3-one* (**3af**): white solid; 39.8 mg, 87% yield; m.p. 209.2–210.0 °C; ^1^H NMR (400 MHz, CDCl_3_) *δ* 7.96 (d, *J* = 8.3 Hz, 2H), 7.50 (d, *J* = 8.2 Hz, 2H), 7.44 (m, 1H), 7.25 (m, 1H), 6.98 (m, 1H), 6.60 (s, 1H), 6.46 (m, 1H), 5.55 (d, *J* = 10.3 Hz, 1H), 3.59 (d, *J* = 6.0 Hz, 1H), 2.89 (dd, *J* = 18.1, 6.2 Hz, 1H), 2.58 (d, *J* = 18.1 Hz, 1H), 2.44 (s, 3H), 1.45 (s, 3H); ^13^C NMR (101 MHz, DMSO-*d*_6_) *δ* 193.2, 158.2 (d, *J_CF_* = 238.8 Hz), 154.3 (d, *J_CF_* = 1.6 Hz), 148.2, 144.1, 139.9, 130.1, 127.5, 126.8 (d, *J* = 9.0 Hz), 126.7, 120.4, 118.3 (d, *J_CF_* = 24.7 Hz), 114.1 (d, *J_CF_* = 26.2 Hz), 111.4 (d, *J_CF_* = 8.6 Hz), 70.8 (d, *J_CF_* = 2.0 Hz), 64.8, 52.7, 32.8, 22.2, 21.0; HRMS (ESI) *m*/*z*: [M + H]^+^ Calcd for C_22_H_20_FN_2_O_6_S: 459.1021, found 459.1012.

*8-Chloro-10a-methyl-4b-nitro-10-tosyl-4,4a,4b,9b,10,10a-hexahydro-3H-benzofuro[3,2-b]indol-3-one* (**3ag**): white solid; 39.5 mg, 83% yield; m.p. 231.2–231.8 °C; ^1^H NMR (400 MHz, DMSO-*d*_6_) *δ* 7.96 (d, *J* = 8.3 Hz, 2H), 7.67 (d, *J* = 2.2 Hz, 1H), 7.50 (d, *J* = 8.2 Hz, 2H), 7.45 (m, 1H), 7.00 (d, *J* = 8.7 Hz, 1H), 6.46 (m, 1H), 5.56 (d, *J* = 10.3 Hz, 1H), 3.59 (d, *J* = 6.0 Hz, 1H), 2.90 (dd, *J* = 18.1, 6.2 Hz, 1H), 2.58 (d, *J* = 18.0 Hz, 1H), 2.44 (s, 3H), 1.46 (s, 3H); ^13^C{^1^H} NMR (101 MHz, DMSO-*d*_6_) *δ* 193.2, 156.9, 148.2, 144.1, 139.8, 131.4, 130.1, 127.5, 127.4, 127.2, 126.7, 120.1, 111.9, 70.6, 64.8, 52.7, 32.7, 22.1, 21.1; HRMS (ESI) *m*/*z*: [M + H]^+^ Calcd for C_22_H_20_ClN_2_O_6_S: 475.0725, found 475.0733.

*8-Bromo-10a-methyl-4b-nitro-10-tosyl-4,4a,4b,9b,10,10a-hexahydro-3H-benzofuro[3,2-b]indol-3-one* (**3ah**): white solid; 43.7 mg, 84% yield; m.p. 214.6–215.1 °C; ^1^H NMR (400 MHz, DMSO-*d*_6_) *δ* 7.95 (d, *J* = 8.3 Hz, 2H), 7.80 (d, *J* = 2.0 Hz, 1H), 7.57 (m, 1H), 7.50 (d, *J* = 8.2 Hz, 2H), 6.95 (d, *J* = 8.7 Hz, 1H), 6.61 (s, 1H), 6.46 (d, *J* = 10.3 Hz, 1H), 5.56 (d, *J* = 10.3 Hz, 1H), 3.59 (d, *J* = 6.0 Hz, 1H), 2.89 (dd, *J* = 18.1, 6.2 Hz, 1H), 2.58 (d, *J* = 18.1 Hz, 1H), 2.44 (s, 3H), 1.46 (s, 3H); ^13^C NMR (101 MHz, DMSO-*d*_6_) *δ* 193.2, 157.4, 148.2, 144.1, 139.8, 134.2, 130.1, 130.0, 127.9, 127.5, 126.7, 120.0, 115.0, 112.4, 70.5, 64.8, 52.6, 32.7, 22.1, 21.1; HRMS (ESI) *m*/*z*: [M + H]^+^ Calcd for C_22_H_20_BrN_2_O_6_S: 519.0220, found 519.0187.

*10a-Methyl-4b,8-dinitro-10-tosyl-4,4a,4b,9b,10,10a-hexahydro-3H-benzofuro[3,2-b]indol-3-one* (**3ai**): white solid; 29.7 mg, 61% yield; m.p. 242.3–243.0 °C; ^1^H NMR (400 MHz, CDCl_3_) *δ* 8.78 (s, 1H), 8.31 (d, *J* = 8.9 Hz, 1H), 7.87 (d, *J* = 7.8 Hz, 2H), 7.41 (d, *J* = 7.8 Hz, 2H), 7.01 (d, *J* = 8.9 Hz, 1H), 6.61 (d, *J* = 10.3 Hz, 1H), 6.26 (s, 1H), 5.61 (d, *J* = 10.3 Hz, 1H), 3.47 (d, *J* = 5.7 Hz, 1H), 2.88 (d, *J* = 18.1 Hz, 1H), 2.63 (dd, *J* = 18.1, 6.0 Hz, 1H), 2.49 (s, 3H), 1.54 (s, 3H); ^13^C{^1^H} NMR (101 MHz, CDCl_3_) *δ* 192.1, 162.5, 148.4, 145.0, 144.9, 139.7, 130.4, 128.6, 128.1, 126.9, 126.1, 124.9, 120.6, 111.1, 71.0, 64.9, 53.6, 32.8, 23.1, 21.7; HRMS (ESI) *m*/*z*: [M + Na]^+^ Calcd for C_22_H_19_N_3_O_8_SNa: 508.0785, found 508.0792.

*7,10a-Dimethyl-4b-nitro-10-tosyl-4,4a,4b,9b,10,10a-hexahydro-3H-benzofuro[3,2-b]indol-3-one* (**3aj**): white solid; 42.4 mg, 93% yield; m.p. 238.0–238.6 °C; ^1^H NMR (400 MHz, DMSO-*d*_6_) *δ* 7.95 (d, *J* = 8.2 Hz, 2H), 7.60 (d, *J* = 7.8 Hz, 1H), 7.49 (d, *J* = 8.2 Hz, 2H), 6.97 (d, *J* = 7.8 Hz, 1H), 6.75 (s, 1H), 6.50 (s, 1H), 6.40 (d, *J* = 10.3 Hz, 1H), 5.53 (d, *J* = 10.3 Hz, 1H), 3.58 (d, *J* = 5.9 Hz, 1H), 2.88 (dd, *J* = 18.1, 6.2 Hz, 1H), 2.57 (d, *J* = 18.0 Hz, 1H), 2.44 (s, 3H), 2.31 (s, 3H), 1.43 (s, 3H); ^13^C{^1^H} NMR (101 MHz, DMSO-*d*_6_) *δ* 193.8, 158.9, 148.7, 144.3, 142.3, 140.6, 130.5, 127.8, 127.7, 127.1, 125.1, 122.6, 120.5, 110.9, 71.3, 64.8, 52.9, 33.2, 22.7, 21.5, 21.5; HRMS (ESI) *m*/*z*: [M + H]^+^ Calcd for C_23_H_23_N_2_O_6_S: 455.1271, found 455.1270.

*7-Methoxy-10a-methyl-4b-nitro-10-tosyl-4,4a,4b,9b,10,10a-hexahydro-3H-benzofuro[3,2-b]indol-3-one* (**3ak**): white solid; 43.1 mg, 92% yield; m.p. 229.6–230.2 °C; ^1^H NMR (400 MHz, DMSO-*d*_6_) *δ* 7.95 (d, *J* = 8.2 Hz, 2H), 7.60 (d, *J* = 8.5 Hz, 1H), 7.48 (d, *J* = 8.2 Hz, 2H), 6.72 (m, 1H), 6.52 (d, *J* = 2.1 Hz, 1H), 6.46 (s, 1H), 6.43 (d, *J* = 10.3 Hz, 1H), 5.58 (d, *J* = 10.3 Hz, 1H), 3.75 (s, 3H), 3.58 (d, *J* = 6.0 Hz, 1H), 2.88 (dd, *J* = 18.2, 6.2 Hz, 1H), 2.57 (d, *J* = 18.1 Hz, 1H), 2.43 (s, 3H), 1.43 (s, 3H); ^13^C{^1^H} NMR (101 MHz, DMSO-*d*_6_) *δ* 193.7, 162.7, 160.0, 148.8, 144.3, 140.6, 130.5, 128.5, 127.7, 127.1, 120.9, 117.3, 110.6, 96.4, 71.1, 64.7, 56.2, 52.9, 33.2, 22.7, 21.5; HRMS (ESI) *m*/*z*: [M + H]^+^ Calcd for C_23_H_23_N_2_O_7_S: 471.1220, found 471.1222.

*7-Fluoro-10a-methyl-4b-nitro-10-tosyl-4,4a,4b,9b,10,10a-hexahydro-3H-benzofuro[3,2-b]indol-3-one* (**3al**): white solid; 38.1 mg, 83% yield; m.p. 223.0–223.6 °C; ^1^H NMR (400 MHz, DMSO-*d*_6_) *δ* 7.95 (d, *J* = 8.2 Hz, 2H), 7.74 (m, 1H), 7.49 (d, *J* = 8.2 Hz, 2H), 7.01 (m, 1H), 6.95 (m, 1H), 6.57 (s, 1H), 6.47–6.38 (m, 1H), 5.58 (d, *J* = 10.3 Hz, 1H), 3.60 (d, *J* = 5.9 Hz, 1H), 2.90 (dd, *J* = 18.2, 6.2 Hz, 1H), 2.58 (d, *J* = 18.1 Hz, 1H), 2.44 (s, 3H), 1.45 (s, 3H); ^13^C{^1^H} NMR (101 MHz, DMSO-*d*_6_) *δ* 193.17, 163.99 (d, *J_CF_* = 246.7 Hz), 159.10 (d, *J_CF_* = 13.7 Hz), 148.33, 144.00, 140.01, 130.08, 128.80 (d, *J_CF_* = 10.6 Hz), 127.4, 126.7, 121.5 (d, *J_CF_* = 2.6 Hz), 120.5, 110.9 (d, *J_CF_* = 22.9 Hz), 98.7 (d, *J_CF_* = 27.6 Hz), 70.3, 64.5, 52.6, 32.7, 22.2, 21.0; HRMS (ESI) *m*/*z*: [M + H]^+^ Calcd for C_22_H_20_FN_2_O_6_S: 459.1021, found 459.1013.

*7-Chloro-10a-methyl-4b-nitro-10-tosyl-4,4a,4b,9b,10,10a-hexahydro-3H-benzofuro[3,2-b]indol-3-one* (**3am**): white solid; 39.3 mg, 83% yield; m.p. 253.1–253.5 °C; ^1^H NMR (400 MHz, DMSO-*d*_6_) *δ* 7.95 (d, *J* = 8.3 Hz, 2H), 7.72 (d, *J* = 8.2 Hz, 1H), 7.50 (d, *J* = 8.2 Hz, 2H), 7.25 (m, 1H), 7.15 (d, *J* = 1.6 Hz, 1H), 6.59 (s, 1H), 6.42 (m, 1H), 5.58 (d, *J* = 10.3 Hz, 1H), 3.59 (d, *J* = 6.0 Hz, 1H), 2.90 (dd, *J* = 18.2, 6.2 Hz, 1H), 2.57 (d, *J* = 18.1 Hz, 1H), 2.44 (s, 3H), 1.44 (s, 3H); ^13^C{^1^H} NMR (101 MHz, DMSO-*d*_6_) *δ* 193.2, 158.8, 148.3, 144.0, 139.9, 135.6, 130.1, 128.7, 127.5, 126.7, 124.5, 124.2, 120.2, 110.6, 70.3, 64.6, 52.6, 32.7, 22.1, 21.0; HRMS (ESI) *m*/*z*: [M + H]^+^ Calcd for C_22_H_20_ClN_2_O_6_S: 475.0725, found 475.0731.

*7-Bromo-10a-methyl-4b-nitro-10-tosyl-4,4a,4b,9b,10,10a-hexahydro-3H-benzofuro[3,2-b]indol-3-one* (**3an**): white solid; 43.5 mg, 84% yield; m.p. 253.4–254.1 °C; ^1^H NMR (400 MHz, DMSO-*d*_6_) *δ* 7.95 (d, *J* = 8.3 Hz, 2H), 7.66 (d, *J* = 8.2 Hz, 1H), 7.50 (d, *J* = 8.2 Hz, 2H), 7.39 (m, 1H), 7.26 (d, *J* = 1.4 Hz, 1H), 6.57 (s, 1H), 6.43 (d, *J* = 10.3 Hz, 1H), 5.58 (d, *J* = 10.3 Hz, 1H), 3.59 (d, *J* = 6.0 Hz, 1H), 2.90 (dd, *J* = 18.2, 6.2 Hz, 1H), 2.58 (d, *J* = 18.0 Hz, 1H), 2.44 (s, 3H), 1.44 (s, 3H); ^13^C{^1^H} NMR (101 MHz, DMSO-*d*_6_) *δ* 193.2, 158.8, 148.3, 144.0, 139.9, 130.1, 129.1, 127.5, 127.1, 126.7, 124.9, 123.8, 120.1, 113.4, 70.4, 64.6, 52.6, 32.7, 22.1, 21.1; HRMS (ESI) *m*/*z*: [M + H]^+^ Calcd for C_22_H_20_BrN_2_O_6_S: 519.0220, found 519.0242.

*6,10a-Dimethyl-4b-nitro-10-tosyl-4,4a,4b,9b,10,10a-hexahydro-3H-benzofuro[3,2-b]indol-3-one* (**3ao**): white solid; 43.3 mg, 95% yield; m.p. 215.6–216.2 °C; ^1^H NMR (400 MHz, DMSO-*d*_6_) *δ* 7.95 (d, *J* = 8.2 Hz, 2H), 7.54 (d, *J* = 7.5 Hz, 1H), 7.48 (s, 2H), 7.21 (d, *J* = 7.4 Hz, 1H), 7.04 (m, 1H), 6.57 (s, 1H), 6.34 (d, *J* = 9.0 Hz, 1H), 5.50 (d, *J* = 10.3 Hz, 1H), 3.57 (d, *J* = 5.8 Hz, 1H), 2.89 (dd, *J* = 17.9, 6.0 Hz, 1H), 2.62 (d, *J* = 17.8 Hz, 1H), 2.44 (s, 3H), 2.06 (s, 3H), 1.43 (s, 3H); ^13^C{^1^H} NMR (101 MHz, DMSO-*d_6_*) *δ* 193.7, 156.6, 148.2, 143.9, 140.2, 132.2, 130.0, 127.3, 126.7, 124.8, 124.5, 123.7, 120.1, 119.4, 71.4, 64.4, 52.8, 33.0, 22.1, 21.0, 14.1; HRMS (ESI) *m*/*z*: [M + Na]^+^ Calcd for C_23_H_22_N_2_O_6_SNa: 477.1091, found 477.1094.

*6-(tert-Butyl)-10a-methyl-4b-nitro-10-tosyl-4,4a,4b,9b,10,10a-hexahydro-3H-benzofuro[3,2-b]indol-3-one* (**3ap**): white solid; 43.8 mg, 88% yield; m.p. 210.6–211.5 °C; ^1^H NMR (400 MHz, CDCl_3_) δ 7.81 (d, *J* = 7.9 Hz, 2H), 7.70 (d, *J* = 7.5 Hz, 1H), 7.31 (d, *J* = 7.9 Hz, 2H), 7.23 (d, *J* = 7.7 Hz, 1H), 7.01 (m, 1H), 6.52 (d, *J* = 10.3 Hz, 1H), 6.10 (s, 1H), 5.54 (d, *J* = 10.3 Hz, 1H), 3.39 (d, *J* = 6.5 Hz, 1H), 2.81 (d, *J* = 18.8 Hz, 1H), 2.53 (dd, *J* = 18.8, 6.7 Hz, 1H), 2.40 (s, 3H), 1.44 (s, 3H), 1.24 (s, 9H); ^13^C{^1^H} NMR (101 MHz, CDCl_3_) *δ* 192.3, 156.2, 149.3, 144.4, 140.5, 134.9, 130.3, 128.8, 128.0, 126.9, 125.9, 125.0, 124.4, 120.0, 71.9, 64.5, 53.3, 34.3, 32.3, 29.9, 23.5, 21.7; HRMS (ESI) *m*/*z*: [M + Na]^+^ Calcd for C_26_H_28_N_2_O_6_SNa: 519.1560, found 519.1567.

*6-Methoxy-10a-methyl-4b-nitro-10-tosyl-4,4a,4b,9b,10,10a-hexahydro-3H-benzofuro[3,2-b]indol-3-one* (**3aq**): white solid; 44.2 mg, 94% yield; m.p. 232.4–233.0 °C; ^1^H NMR (400 MHz, DMSO-*d*_6_) *δ* 7.95 (d, *J* = 8.1 Hz, 2H), 7.49 (d, *J* = 8.1 Hz, 2H), 7.31 (d, *J* = 6.8 Hz, 1H), 7.09 (d, *J* = 6.8 Hz, 2H), 6.57 (s, 1H), 6.36 (d, *J* = 10.4 Hz, 1H), 5.54 (d, *J* = 10.3 Hz, 1H), 3.77 (s, 3H), 3.57 (d, *J* = 5.9 Hz, 1H), 2.86 (dd, *J* = 18.3, 6.2 Hz, 1H), 2.59 (d, *J* = 18.2 Hz, 1H), 2.44 (s, 3H), 1.43 (s, 3H); ^13^C{^1^H} NMR (101 MHz, DMSO-*d*_6_) *δ* 192.9, 148.1, 146.6, 144.0, 143.9, 140.1, 130.1, 127.3, 126.7, 126.3, 124.6, 120.0, 118.9, 115.1, 71.4, 64.5, 56.4, 52.4, 32.5, 22.3, 21.0; HRMS (ESI) *m*/*z*: [M + H]^+^ Calcd for C_23_H_23_N_2_O_7_S:471.1220, found 471.1221.

*6-Ethoxy-10a-methyl-4b-nitro-10-tosyl-4,4a,4b,9b,10,10a-hexahydro-3H-benzofuro[3,2-b]indol-3-one* (**3ar**): white solid; 41.5 mg, 86% yield; m.p. 218.9–219.4 °C; ^1^H NMR (400 MHz, DMSO-*d*_6_) *δ* 7.95 (d, *J* = 8.2 Hz, 2H), 7.49 (d, *J* = 8.2 Hz, 2H), 7.31 (m, 1H), 7.11–7.00 (m, 2H), 6.57 (s, 1H), 6.36 (d, *J* = 10.3 Hz, 1H), 5.52 (d, *J* = 10.3 Hz, 1H), 4.05 (m, 2H), 3.57 (d, *J* = 5.9 Hz, 1H), 2.85 (dd, *J* = 18.2, 6.2 Hz, 1H), 2.63 (d, *J* = 18.2 Hz, 1H), 2.44 (s, 3H), 1.43 (s, 3H), 1.27 (t, *J* = 7.0 Hz, 3H); ^13^C{^1^H} NMR (101 MHz, DMSO-*d*_6_) *δ* 192.9, 148.0, 147.1, 143.9, 143.0, 140.2, 130.0, 127.3, 126.7, 126.4, 124.6, 119.9, 118.9, 116.5, 71.4, 64.8, 64.4, 52.5, 32.5, 22.3, 21.0, 14.6; HRMS (ESI) *m*/*z*: [M + H]^+^ Calcd for C_24_H_25_N_2_O_7_S: 485.1377, found 485.1372.

*6-Fluoro-10a-methyl-4b-nitro-10-tosyl-4,4a,4b,9b,10,10a-hexahydro-3H-benzofuro[3,2-b]indol-3-one* (**3as**): white solid; 45.0 mg, 98% yield; m.p. 214.3–214.7 °C; ^1^H NMR (400 MHz, DMSO-*d*_6_) *δ* 7.96 (d, *J* = 8.2 Hz, 2H), 7.56 (d, *J* = 7.6 Hz, 1H), 7.50 (d, *J* = 8.2 Hz, 2H), 7.42–7.30 (m, 1H), 7.18 (m, 1H), 6.69 (s, 1H), 6.40 (m, 1H), 5.58 (d, *J* = 10.3 Hz, 1H), 3.62 (d, *J* = 5.9 Hz, 1H), 2.90 (dd, *J* = 18.2, 6.2 Hz, 1H), 2.64 (d, *J* = 18.1 Hz, 1H), 2.44 (s, 3H), 1.45 (s, 3H); ^13^C{^1^H} NMR (101 MHz, DMSO-*d*_6_) *δ* 193.1, 148.2, 145.8 (d, *J_CF_* = 263.4 Hz), 144.6 (d, *J_CF_* = 5.1 Hz), 144.1, 139.9, 130.1, 128.8, 127.4, 126.7, 125.1 (d, *J_CF_* = 5.4 Hz), 123.2 (d, *J_CF_* = 3.6 Hz), 120.4, 118.2 (d, *J_CF_* = 15.9 Hz), 71.1 (d, *J_CF_* = 2.1 Hz), 64.7, 52.7, 32.7, 22.2, 21.1; HRMS (ESI) *m*/*z*: [M + H]^+^ Calcd for C_22_H_20_FN_2_O_6_S: 459.1021, found 459.1024.

*6-Chloro-10a-methyl-4b-nitro-10-tosyl-4,4a,4b,9b,10,10a-hexahydro-3H-benzofuro[3,2-b]indol-3-one* (**3at**): white solid; 34.9 mg, 74% yield; m.p. 223.0–223.8 °C; ^1^H NMR (400 MHz, DMSO-*d*_6_) *δ* 7.95 (d, *J* = 8.2 Hz, 2H), 7.69 (d, *J* = 7.6 Hz, 1H), 7.57–7.39 (m, 3H), 7.19 (m, 1H), 6.70 (s, 1H), 6.41–6.32 (m, 1H), 5.56 (d, *J* = 10.3 Hz, 1H), 3.61 (d, *J* = 5.9 Hz, 1H), 2.89 (dd, *J* = 18.2, 6.2 Hz, 1H), 2.65 (d, *J* = 18.2 Hz, 1H), 2.44 (s, 3H), 1.45 (s, 3H); ^13^C{^1^H} NMR (101 MHz, DMSO-*d*_6_) *δ* 192.8, 153.8, 148.0, 144.1, 139.9, 131.2, 130.1, 127.5, 127.3, 126.7, 126.2, 125.3, 119.6, 114.4, 71.4, 64.6, 52.5, 32.6, 22.1, 21.0; HRMS (ESI) *m*/*z*: [M + H]^+^ Calcd for C_22_H_20_ClN_2_O_6_S: 475.0725, found 475.0726.

*6-Bromo-10a-methyl-4b-nitro-10-tosyl-4,4a,4b,9b,10,10a-hexahydro-3H-benzofuro[3,2-b]indol-3-one* (**3au**): white solid; 42.7 mg, 82% yield; m.p. 218.6–219.0 °C; ^1^H NMR (400 MHz, DMSO-*d*_6_) *δ* 7.95 (d, *J* = 8.1 Hz, 2H), 7.71 (d, *J* = 7.6 Hz, 1H), 7.61 (d, *J* = 8.0 Hz, 1H), 7.50 (d, *J* = 8.1 Hz, 2H), 7.12 (m, 1H), 6.70 (s, 1H), 6.37 (d, *J* = 10.3 Hz, 1H), 5.55 (d, *J* = 10.3 Hz, 1H), 3.59 (d, *J* = 5.8 Hz, 1H), 2.88 (dd, *J* = 18.3, 6.2 Hz, 1H), 2.63 (d, *J* = 18.2 Hz, 1H), 2.44 (s, 3H), 1.44 (s, 3H); ^13^C{^1^H} NMR (101 MHz, DMSO-*d*_6_) *δ* 192.7, 155.2, 148.0, 144.1, 139.9, 134.1, 130.1, 127.6, 126.9, 126.8, 126.7, 125.6, 119.3, 102.1, 71.6, 64.6, 52.5, 32.5, 22.2, 21.1; HRMS (ESI) *m*/*z*: [M + Na]^+^ Calcd for C_22_H_19_BrN_2_O_6_SNa: 541.0039, found 541.0041.

*6,8-Di-tert-butyl-10a-methyl-4b-nitro-10-tosyl-4,4a,4b,9b,10,10a-hexahydro-3H-benzofuro[3,2-b]indol-3-one* (**3av**): white solid; 45.4 mg, 82% yield; m.p. 194.4–195.2 °C; ^1^H NMR (400 MHz, CDCl_3_) *δ* 7.88 (d, *J* = 7.9 Hz, 2H), 7.77 (s, 1H), 7.38 (d, *J* = 7.9 Hz, 2H), 7.32 (s, 1H), 6.54 (d, *J* = 10.3 Hz, 1H), 6.16 (s, 1H), 5.63 (d, *J* = 10.3 Hz, 1H), 3.46 (d, *J* = 6.3 Hz, 1H), 2.87 (d, *J* = 18.7 Hz, 1H), 2.61 (dd, *J* = 18.8, 6.6 Hz, 1H), 2.47 (s, 3H), 1.55 (s, 3H), 1.32 (s, 9H), 1.31 (s, 9H); ^13^C{^1^H} NMR (101 MHz, CDCl_3_) *δ* 192.5, 154.0, 149.2, 147.4, 144.3, 140.5, 133.8, 130.2, 128.0, 126.9, 125.9, 124.5, 122.6, 120.3, 72.0, 64.6, 53.2, 35.0, 34.4, 32.3, 31.7, 29.9, 23.7, 21.7; HRMS (ESI) *m*/*z*: [M + H]^+^ Calcd for C_30_H_37_N_2_O_6_S: 553.2367, found 553.2361.

*8-Bromo-6-methoxy-10a-methyl-4b-nitro-10-tosyl-4,4a,4b,9b,10,10a-hexahydro-3H-benzofuro[3,2-b]indol-3-one* (**3aw**): white solid; 47.4 mg, 86% yield; m.p. 224.8–225.2 °C; ^1^H NMR (400 MHz, CDCl_3_) *δ* 7.85 (d, *J* = 8.0 Hz, 2H), 7.61 (s, 1H), 7.39 (d, *J* = 7.9 Hz, 2H), 7.03 (s, 1H), 6.57 (d, *J* = 10.4 Hz, 1H), 6.21 (s, 1H), 5.63 (d, *J* = 10.4 Hz, 1H), 3.84 (s, 3H), 3.40 (d, *J* = 5.7 Hz, 1H), 2.90 (d, *J* = 18.2 Hz, 1H), 2.57 (dd, *J* = 18.2, 6.2 Hz, 1H), 2.48 (s, 3H), 1.50 (s, 3H); ^13^C{^1^H} NMR (101 MHz, CDCl_3_) *δ* 192.3, 148.4, 146.2, 145.0, 144.6, 140.1, 130.3, 127.9, 127.1, 126.9, 122.2, 120.1, 118.7, 116.8, 72.2, 64.8, 57.1, 53.7, 32.7, 23.0, 21.7; HRMS (ESI) *m*/*z*: [M + Na]^+^ Calcd for C_23_H_21_BrN_2_O_7_SNa: 571.0145, found 571.0153.

*11a-Methyl-7a-nitro-12-tosyl-7a,7b,8,11a,12,12a-hexahydro-9H-naphtho[1′,2′:4,5]furo[3,2-b]indol-9-one* (**3ax**): white solid; 42.3 mg, 86% yield; m.p. 264.7–265.2 °C; ^1^H NMR (400 MHz, DMSO-*d*_6_) *δ* 8.79 (d, *J* = 8.5 Hz, 1H), 8.04 (d, *J* = 9.0 Hz, 1H), 8.00 (d, *J* = 8.2 Hz, 1H), 7.97 (d, *J* = 8.3 Hz, 2H), 7.66 (m, 1H), 7.50 (m, 3H), 7.24 (s, 1H), 7.13 (d, *J* = 8.9 Hz, 1H), 6.02 (d, *J* = 11.7 Hz, 1H), 5.30 (d, *J* = 10.3 Hz, 1H), 3.45 (d, *J* = 5.4 Hz, 1H), 2.91 (dd, *J* = 18.3, 5.9 Hz, 1H), 2.66 (d, *J* = 18.2 Hz, 1H), 2.44 (s, 3H), 1.57 (s, 3H); ^13^C{^1^H} NMR (101 MHz, DMSO-*d*_6_) *δ* 193.2, 156.6, 149.1, 144.5, 138.5, 133.7, 130.5, 130.2, 130.0, 129.2, 128.1, 127.6, 127.1, 124.7, 123.2, 121.2, 115.8, 111.2, 71.7, 65.8, 52.8, 32.8, 22.0, 21.1; HRMS (ESI) *m*/*z*: [M + H]^+^ Calcd for C_26_H_23_N_2_O_6_S: 491.1271, found 491.1262.

*10a-Methyl-4b-nitro-5,10-ditosyl-4a,4b,5,9b,10,10a-hexahydroindolo[3,2-b]indol-3(4H)-one* (**3ay**): white solid; 35.6 mg, 60% yield; m.p. 206.5–207.4 °C; ^1^H NMR (400 MHz, CDCl_3_) *δ* 7.77 (m, 3H), 7.63 (d, *J* = 7.8 Hz, 2H), 7.38 (d, *J* = 8.2 Hz, 1H), 7.35–7.26 (m, 5H), 7.12 (m, 1H), 6.42 (d, *J* = 10.4 Hz, 1H), 6.21 (s, 1H), 5.45 (d, *J* = 10.4 Hz, 1H), 4.13 (d, *J* = 5.1 Hz, 1H), 3.57 (d, *J* = 18.7 Hz, 1H), 2.70 (dd, *J* = 18.8, 6.3 Hz, 1H), 2.46 (s, 3H), 2.40 (s, 3H), 1.56 (s, 3H); ^13^C{^1^H} NMR (101 MHz, CDCl_3_) *δ* 193.0, 149.8, 145.7, 144.6, 141.9, 139.5, 134.6, 131.0, 130.3, 129.6, 128.7, 127.8, 127.4, 126.7, 126.6, 125.3, 113.5, 112.1, 75.8, 65.6, 51.7, 34.0, 23.3, 21.8, 21.7; HRMS (ESI) *m*/*z*: [M + H]^+^ Calcd for C_29_H_28_N_3_O_7_S_2_: 594.1363, found 594.1366.

### 3.3. Scale-Up Experiment for Preparation of Compound ***3aa***

To an ordinary vial charged with a magnetic stirring bar, **1a** (2.080 g, 7.5 mmol), **2a** (0.816 g, 5.0 mmol), K_2_CO_3_ (0.691 g), and CH_3_CN (100 mL) were added. Then, the mixture was stirred at 65 °C for 72 h. Product **3aa** was isolated by flash chromatography on silica gel as a white solid (2.019 g, 92% yield).

### 3.4. Procedure for Synthesis of Compound ***4***

In a reactor, MeOH (5.0 mL) was added to substrate **3aa** (0.10 mmol) and the mixture was stirred at 0 °C for 10 min. Then, NaBH_4_ (0.15 mmol) was added in one portion. The mixture was stirred at the same temperature for 15 min, then at 25 °C for 2 h. Upon reaction completion, saturated NH_4_Cl (5.0 mL) was added and the mixture was extracted with DCM. The organic phase was dried over anhydrous Mg_2_SO_4_, filtered, and concentrated to afford the crude product. The crude product was purified by flash chromatography with PE/EA (*v*/*v* = 5:1) to provide the desired compound **4** at 95% yield (42.1 mg) as a white solid.

*10a-Methyl-4b-nitro-10-tosyl-4,4a,4b,9b,10,10a-hexahydro-3H-benzofuro[3,2-b]indol-3-ol* (**4**): white solid; 42.1 mg, 95% yield; m.p. 187.5–188.1 °C; ^1^H NMR (400 MHz, CDCl_3_) *δ* 7.91 (d, *J* = 7.6 Hz, 1H), 7.82 (d, *J* = 7.8 Hz, 2H), 7.35 (m, 3H), 7.14 (m, 1H), 6.95 (d, *J* = 8.2 Hz, 1H), 6.24 (s, 1H), 5.60 (d, *J* = 10.4 Hz, 1H), 5.46 (d, *J* = 10.4 Hz, 1H), 4.01 (s, 1H), 3.07 (d, *J* = 5.6 Hz, 2H), 2.44 (s, 3H), 2.35 (m, 1H), 2.15 (d, *J* = 16.6 Hz, 1H), 1.32 (s, 3H); ^13^C{^1^H} NMR (101 MHz, CDCl_3_) *δ* 157.6, 144.0, 140.6, 131.6, 131.4, 130.0, 129.5, 128.6, 126.8, 125.0, 124.4, 120.3, 110.1, 72.0, 65.2, 59.7, 50.1, 26.9, 24.3, 21.6; HRMS (ESI) *m*/*z*: [M + Na]^+^ Calcd for C_22_H_22_N_2_O_6_SNa: 465.1091, found 465.1100.

### 3.5. Procedure for Synthesis of Product ***5***

To a stirred solution of substrate **3aa** (0.10 mmol) and NaOH (0.3 mmol) in MeOH (1.0 mL) and THF (1.0 mL), H_2_O_2_ solution (30% wt, 5.0 equiv) was added. The mixture was stirred at 30 °C for 24 h. Upon reaction completion, saturated Na_2_S_2_O_3_ (3.0 mL) was added and the mixture was extracted with EA. The organic phase was dried over anhydrous Mg_2_SO_4_, filtered, and concentrated to afford the crude product. The crude product was purified by flash chromatography with PE/EA (*v*/*v* = 5:1) to provide the desired compound **5** at 98% yield (44.8 mg) as a white solid.

*9a-Methyl-3b-nitro-9-tosyl-1a,3,3a,3b,8b,9,9a,9b-octahydro-2H-benzofuro[3,2-b]oxireno[2,3-g]indol-2-one* (**5**): white solid; 44.8 mg, 98% yield; m.p. 200.4–200.9 °C; ^1^H NMR (400 MHz, DMSO-*d*_6_) *δ* 7.98 (d, *J* = 7.9 Hz, 2H), 7.80 (d, *J* = 7.5 Hz, 1H), 7.50 (d, *J* = 7.9 Hz, 2H), 7.46 (m, 1H), 7.23 (m, 1H), 7.03 (d, *J* = 8.1 Hz, 1H), 6.65 (s, 1H), 3.44 (s, 1H), 3.22 (s, 1H), 2.87 (s, 1H), 2.77 (dd, *J* = 17.2, 4.5 Hz, 1H), 2.45 (s, 3H), 2.37 (d, *J* = 17.1 Hz, 1H), 1.47 (s, 3H); ^13^C{^1^H} NMR (101 MHz, DMSO-*d*_6_) *δ* 200.3, 156.9, 144.1, 139.9, 131.7, 130.1, 127.8, 126.8, 125.4, 124.3, 119.5, 110.3, 71.7, 62.6, 61.6, 55.6, 53.4, 30.6, 21.1, 19.9; HRMS (ESI) *m*/*z*: [M + Na]^+^ Calcd for C_22_H_20_N_2_O_7_SNa: 479.0883, found 479.0880.

### 3.6. Procedure for Synthesis of Product ***6***

To a stirred solution of substrate **3aa** (0.20 mmol) in EtOH (10 mL), Pd/C (10% w.t.) was added. The mixture was stirred at 25 °C for 5 days under hydrogen atmosphere (1 atm). Upon reaction completion, the mixture was filtered then washed with DCM (10 mL). The filtrate was concentrated to afford the crude product, which was purified by flash chromatography with PE/EA (*v*/*v* = 5:1) to provide the desired compound **6** at 44% yield (39.1 mg) as a white solid.

*10a-Methyl-4b-nitro-10-tosyl-1,2,4,4a,4b,9b,10,10a-octahydro-3H-benzofuro[3,2-b]indol-3-one* (**6**): white solid; 39.1 mg, 44% yield; m.p. 184.3–185.1 °C; ^1^H NMR (400 MHz, CDCl_3_) *δ* 7.90 (d, *J* = 7.5 Hz, 1H), 7.79 (d, *J* = 7.6 Hz, 2H), 7.36 (m, 3H), 7.16 (m, 1H), 7.05 (d, *J* = 8.1 Hz, 1H), 6.11 (s, 1H), 3.41 (d, *J* = 7.5 Hz, 1H), 2.67 (d, *J* = 16.8 Hz, 1H), 2.45 (m, 4H), 2.26–2.10 (m, 2H), 2.00 (d, *J* = 13.9 Hz, 1H), 1.80 (m, 1H), 1.62 (s, 3H); ^13^C{^1^H} NMR (101 MHz, CDCl_3_) *δ* 204.8, 158.1, 144.1, 140.4, 131.9, 130.1, 128.6, 126.9, 126.6, 124.5, 120.7, 111.3, 71.8, 66.2, 54.5, 36.5, 35.6, 34.4, 23.1, 21.7; HRMS (ESI) *m*/*z*: [M + Na]^+^ Calcd for C_22_H_22_N_2_O_6_SNa: 465.1091, found 465.1095.

## 4. Conclusions

In summary, we have developed an efficient dearomative (3 + 2) cycloaddition reaction of *para*-quinamines and 2-nitrobenzofurans. With the developed protocol, a series of structurally diverse benzofuro[3,2-*b*]indol-3-one derivatives were smoothly obtained in good to excellent yields (up to 98%) with outstanding diastereoselectivities (all cases > 20:1 *dr*). In addition, the scale-up synthesis and the versatile transformations of the product also demonstrated the potential synthetic application of this dearomative (3 + 2) cycloaddition reaction. Importantly, this approach represents the first example of the nitrogen nucleophile-triggered (3 + 2) cycloaddition reaction of 2-nitrobenzofurans. Further exploration of the dearomative cycloaddition reaction of electron-deficient heteroarenes for synthesizing polyheterocyclic compounds with structural diversity is ongoing in our laboratory.

## Figures and Tables

**Table 1:** Optimization of reaction conditions [a].

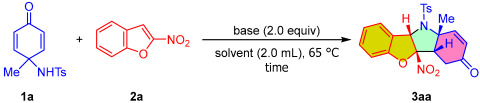					
Entry	Base	Solvent	Time (day)	*dr* [b]	Yield (%) [c]
1	*^t^*BuOK	CH_3_CN	2	>20:1	51
2	K_3_PO_4_	CH_3_CN	2	>20:1	75
3	KOH	CH_3_CN	2	>20:1	51
4	K_2_CO_3_	CH_3_CN	2	>20:1	84
5	DBU	CH_3_CN	5	>20:1	9
6	DIPEA	CH_3_CN	5	-	n.r.
7	Na_2_CO_3_	CH_3_CN	7	>20:1	52
8	Cs_2_CO_3_	CH_3_CN	5	>20:1	52
9	K_2_CO_3_	DCM	7	>20:1	58
10	K_2_CO_3_	THF	7	>20:1	48
11	K_2_CO_3_	MTBE	7	>20:1	49
12	K_2_CO_3_	toluene	7	>20:1	14
13	K_2_CO_3_	EtOH	3	>20:1	6
14	K_2_CO_3_	acetone	3	>20:1	71
15	K_2_CO_3_	EA	7	>20:1	58
16 [d]	K_2_CO_3_	CH_3_CN	2	>20:1	88
17 [e]	K_2_CO_3_	CH_3_CN	2	>20:1	95
18 [e,f]	K_2_CO_3_	CH_3_CN	2	>20:1	84
19 [e,f,g]	K_2_CO_3_	CH_3_CN	2	>20:1	95
20 [h]	NaH	CH_3_CN	8 h	>20:1	75
21 [i]	LiHMDS	THF	12 h	>20:1	48

[a] Unless otherwise noted, the reactions were carried out with **1a** (0.10 mmol), **2a** (0.12 mmol), and base (2.0 equiv) in 2.0 mL of solvent at 65 °C for the specified reaction time. [b] Determined by ^1^H NMR analysis. [c] Isolated yields. [d] The reactions were carried out with **1a** (0.12 mmol) and **2a** (0.10 mmol). [e] The reactions were carried out with **1a** (0.15 mmol) and **2a** (0.10 mmol). [f] The reaction was carried out at 80 °C; [g] 1.0 equiv of K_2_CO_3_ was used. n.r. = no reaction. [h] The reaction was carried out with **1a** (0.15 mmol), **2a** (0.10 mmol), and NaH (1.0 equiv, 60% dispersion in paraffin liquid) in CH_3_CN (2.0 mL) at 0 °C for 8 h. [i] The reaction was carried out with **1a** (0.15 mmol), **2a** (0.10 mmol), and LiHMDS (1.0 equiv, 1 M in THF) in THF (1.0 mL) at −40 °C for 10 h. LiHMDS = lithium bis(trimethylsilyl)amide.

## Data Availability

All the required data are reported in the manuscript and Supplementary Materials.

## Supplementary Materials

The following supporting information can be downloaded at: https://www.mdpi.com/article/10.3390/molecules29051163/s1, X-ray data for product **3ac**; copies of ^1^H, ^13^C NMR spectra. References [60,61,62] are cited in the Supplementary Materials.
