# Supplementary material for: Synthesis of Benzofuro[3,2-b]indol-3-one Derivatives via Dearomative (3 + 2) Cycloaddition of 2-Nitrobenzofurans and para-Quinamines
Source: Molecules. 2024 Mar 5;29(5):1163. doi: 10.3390/molecules29051163 (PMC10934979; doi:10.3390/molecules29051163)

checkCIF/PLATON report

Structure factors have been supplied for datablock(s) 29230101

THIS REPORT IS FOR GUIDANCE ONLY. IF USED AS PART OF A REVIEW PROCEDURE FOR PUBLICATION, IT SHOULD NOT REPLACE THE EXPERTISE OF AN EXPERIENCED CRYSTALLOGRAPHIC REFEREE.

No syntax errors found. CIF dictionary Interpreting this report

Datablock: 29230101

Bond precision:	C-C = 0.0031 A		Wavelength=1.54184
Cell:	a=12.1191(4)	b=12.9353(5)	c=14.2184(5)
	alpha=90	beta=91.613(3)	gamma=90
Temperature:	293 K		

	Calculated	Reported
Volume	2228.05(14)	2228.06(13)
Space group	P 21/c	P 1 21/c 1
Hall group	-P 2ybc	-P 2ybc
Moiety formula	C23 H22 N2 O6 S	C23 H22 N2 O6 S
Sum formula	C23 H22 N2 O6 S	C23 H22 N2 O6 S
Mr	454.49	454.48
Dx,g cm-3	1.355	1.355
Z	4	4
Mu (mm-1)	1.656	1.656
F000	952.0	952.0
F000'	956.30	
h,k,lmax	14,15,16	14,15,16
Nref	3978	3979
Tmin,Tmax	0.793,0.876	0.702,1.000
Tmin'	0.793	

Correction method= # Reported T Limits: Tmin=0.702 Tmax=1.000
AbsCorr = MULTI-SCAN

Data completeness= 1.000 Theta(max)= 67.071

R(reflections)= 0.0435(3310) wR2(reflections)= 0.1277(3979)

S = 1.041 Npar= 292

```
The following ALERTS were generated. Each ALERT has the format
       test-name_ALERT_alert-type_alert-level.
Click on the hyperlinks for more details of the test.
```

Alert level G

```
PLAT199_ALERT_1_G Reported _cell_measurement_temperature ..... (K)        293 Check
PLAT200_ALERT_1_G Reported   _diffrn_ambient_temperature ..... (K)        293 Check
PLAT398_ALERT_2_G Deviating  C-O-C   Angle From 120 for O3       .      107.9 Degree
PLAT909_ALERT_3_G Percentage of I>2sig(I) Data at Theta(Max) Still        76% Note
PLAT978_ALERT_2_G Number C-C Bonds with Positive Residual Density.          6 Info
```

```
   0 ALERT level A = Most likely a serious problem - resolve or explain
   0 ALERT level B = A potentially serious problem, consider carefully
   0 ALERT level C = Check. Ensure it is not caused by an omission or oversight
   5 ALERT level G = General information/check it is not something unexpected

   2 ALERT type 1 CIF construction/syntax error, inconsistent or missing data
   2 ALERT type 2 Indicator that the structure model may be wrong or deficient
   1 ALERT type 3 Indicator that the structure quality may be low
   0 ALERT type 4 Improvement, methodology, query or suggestion
   0 ALERT type 5 Informative message, check
```

It is advisable to attempt to resolve as many as possible of the alerts in all categories. Often the minor alerts point to easily fixed oversights, errors and omissions in your CIF or refinement strategy, so attention to these fine details can be worthwhile. In order to resolve some of the more serious problems it may be necessary to carry out additional measurements or structure refinements. However, the purpose of your study may justify the reported deviations and the more serious of these should normally be commented upon in the discussion or experimental section of a paper or in the "special_details" fields of the CIF. checkCIF was carefully designed to identify outliers and unusual parameters, but every test has its limitations and alerts that are not important in a particular case may appear. Conversely, the absence of alerts does not guarantee there are no aspects of the results needing attention. It is up to the individual to critically assess their own results and, if necessary, seek expert advice.

Publication of your CIF in IUCr journals

A basic structural check has been run on your CIF. These basic checks will be run on all CIFs submitted for publication in IUCr journals (*Acta Crystallographica*, *Journal of Applied Crystallography*, *Journal of Synchrotron Radiation*); however, if you intend to submit to *Acta Crystallographica Section C* or *E* or *IUCrData*, you should make sure that full publication checks are run on the final version of your CIF prior to submission.

Publication of your CIF in other journals

Please refer to the *Notes for Authors* of the relevant journal for any special instructions relating to CIF submission.

PLATON version of 06/07/2023; check.def file version of 30/06/2023

Datablock 29230101 - ellipsoid plot

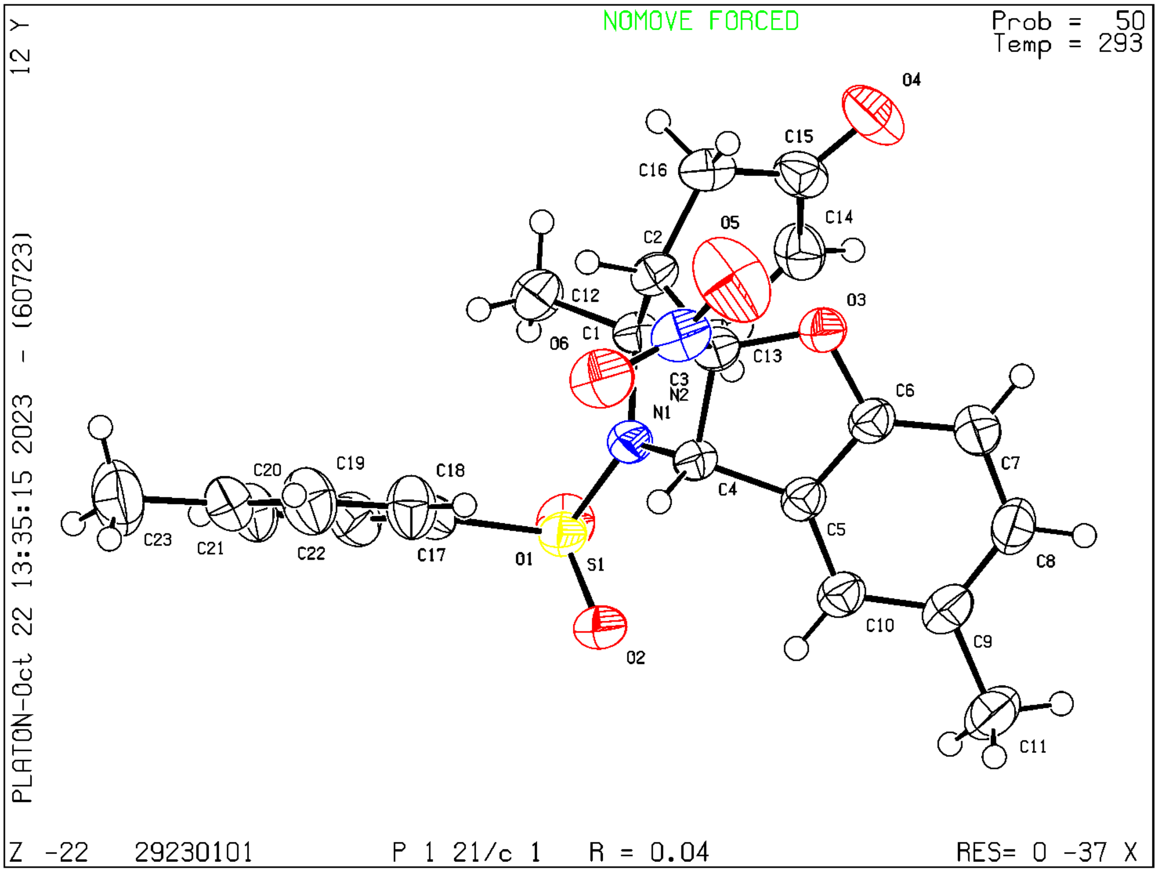

Supplement: Supplementary file 1 [file molecules-29-01163-s001.zip › 3ac-checkcif.pdf]
