# Supplementary material for: Synthesis of Benzofuro[3,2-b]indol-3-one Derivatives via Dearomative (3 + 2) Cycloaddition of 2-Nitrobenzofurans and para-Quinamines
Source: Molecules. 2024 Mar 5;29(5):1163. doi: 10.3390/molecules29051163 (PMC10934979; doi:10.3390/molecules29051163)

Supporting Information

Synthesis of Benzofuro[3,2-*b*]indol-3-one Derivatives via Dearomative (3 + 2) Cycloaddition of 2-Nitrobenzofurans and *para*-Quinamines

Wei-Cheng Yuan,[1,2,*] Hai-Ying Zeng,[1,2] Yan-Ping Zhang,[1] Jian-Qiang Zhao,[1] Yong You,[1] Jun-Qing Yin,[1] Ming-Qiang Zhou[2,*] and Zhen-Hua Wang[1,*]

[1]Innovation Research Center of Chiral Drugs, Institute for Advanced Study, Chengdu University, Chengdu 610106, China

[2]China National Engineering Research Center of Chiral Drugs, Chengdu Institute of Organic Chemistry, Chinese Academy of Sciences, Chengdu 610041, China

yuanwc@cioc.ac.cn

screenfilm@foxmail.com

wangzhenhua@cdu.edu.cn

Table of Contents

1. Crystal data and structure refinement S1
2. Copies of ^{1}H, ^{13}C NMR spectra for compounds **3**, **4**, **5**, and **6**....................S3

1. Crystal data and structure refinement

Single crystals of compound **3ac** were prepared through dissolving the sample in mixture solvent of EtOH/DCM (1/1) at room temperature and crystalizing by slow evaporation of solvent. A suitable crystal was selected for structure determination on a ‘Oxford Gemini E' diffractometer. The crystal was kept at 293 K during data collection. Using Olex2[1], the structure was solved with the ShelXT[2] structure solution program using Intrinsic Phasing and refined with the ShelXL[3] refinement package using Least Squares minimization.

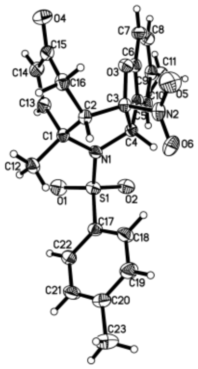

≡

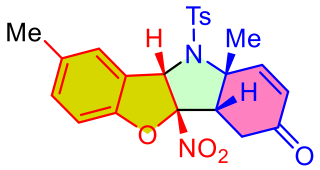

3ac

CCDC 2302663

ORTEP of **3ac** (at the 50% probability level)

Identification code	**3ac**
Empirical formula	$C_{23}H_{22}N_2O_6S$
Formula weight	454.48
Temperature/K	293(2)
Crystal system	monoclinic
Space group	$P2_1/c$
a/Å	12.1191(4)
b/Å	12.9353(5)
c/Å	14.2184(5)
α/°	90
β/°	91.613(3)
γ/°	90
Volume/Å^3	2228.06(13)
Z	4
ρ_{calc}g/cm^3	1.355
μ/mm^{-1}	1.656
F(000)	952.0
Crystal size/mm^3	0.14 × 0.12 × 0.08
Radiation	CuKα (λ = 1.54184)
2Θ range for data collection/°	7.298 to 134.142
Index ranges	$-14 \le h \le 14, -15 \le k \le 15, -16 \le l \le 16$

Reflections collected	20024
Independent reflections	3979 [R_{int} = 0.0558, R_{sigma} = 0.0363]
Data/restraints/parameters	3979/0/292
Goodness-of-fit on F^2	1.041
Final R indexes [I>=2σ (I)]	R_1 = 0.0435, wR_2 = 0.1165
Final R indexes [all data]	R_1 = 0.0538, wR_2 = 0.1277
Largest diff. peak/hole / e $Å^{-3}$	0.26/-0.27

1. Dolomanov, O. V.; Bourhis, L. J.; Gildea, R. J, Howard, J. A. K; Puschmann, H. *J. Appl. Cryst.*, **2009**, *42*, 339-341.
2. Sheldrick, G. M. *Acta Cryst.* **2015**, *A71*, 3-8.
3. Sheldrick, G. M. *Acta Cryst.* **2015**, *C71*, 3-8.

2. Copies of ^{1}H, ^{13}C NMR spectra for compounds 3, 4, 5, and 6

^{1}H NMR (400 MHz, DMSO-d_6) of **3aa**

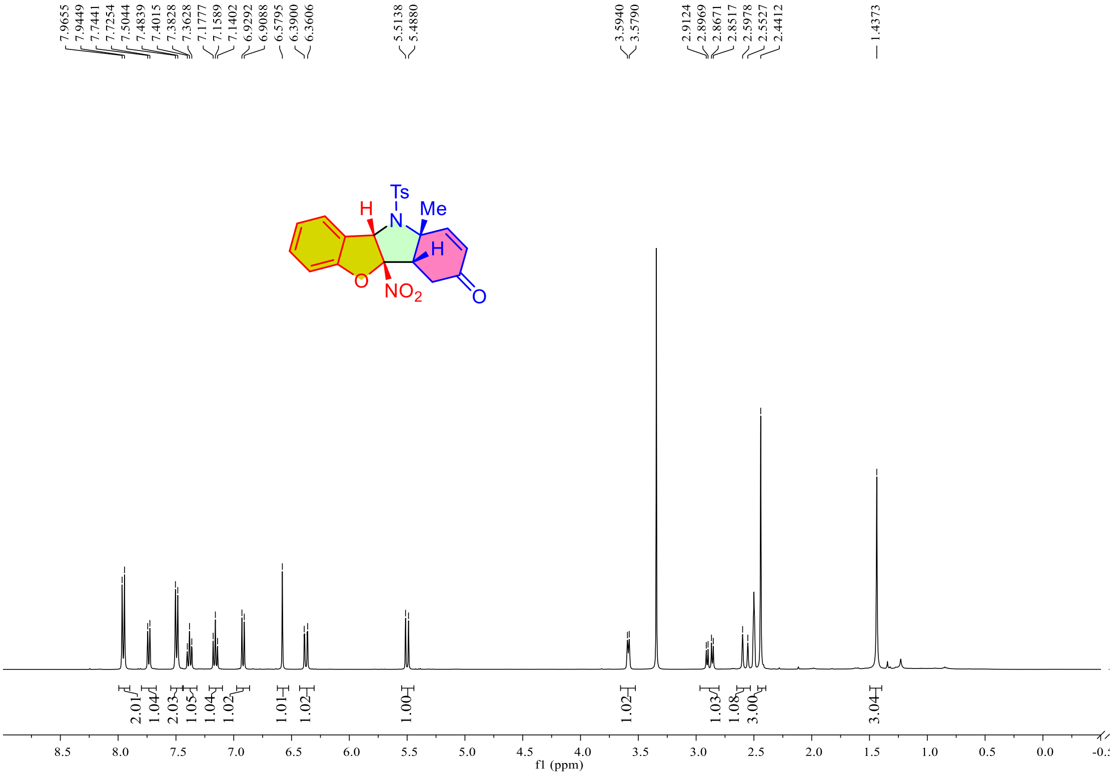

^{13}C NMR (101 MHz, DMSO-d_6) of **3aa**

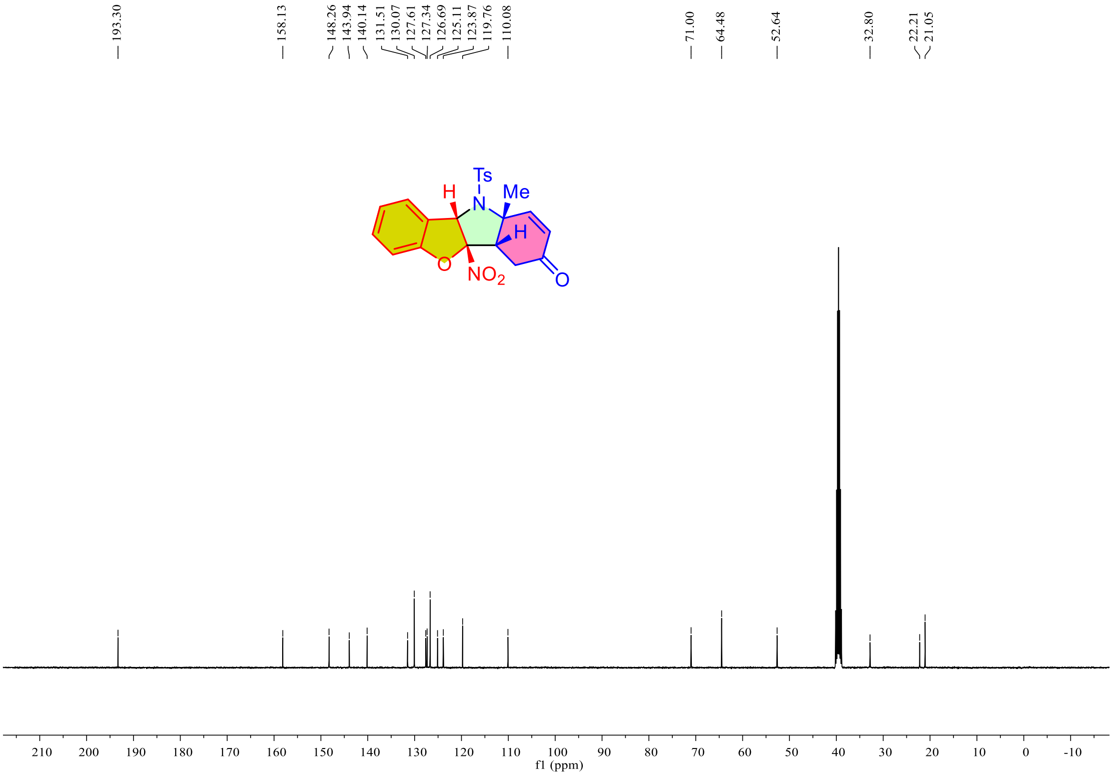

^{1}H NMR (400 MHz, $CDCl_3$) of **3ba**

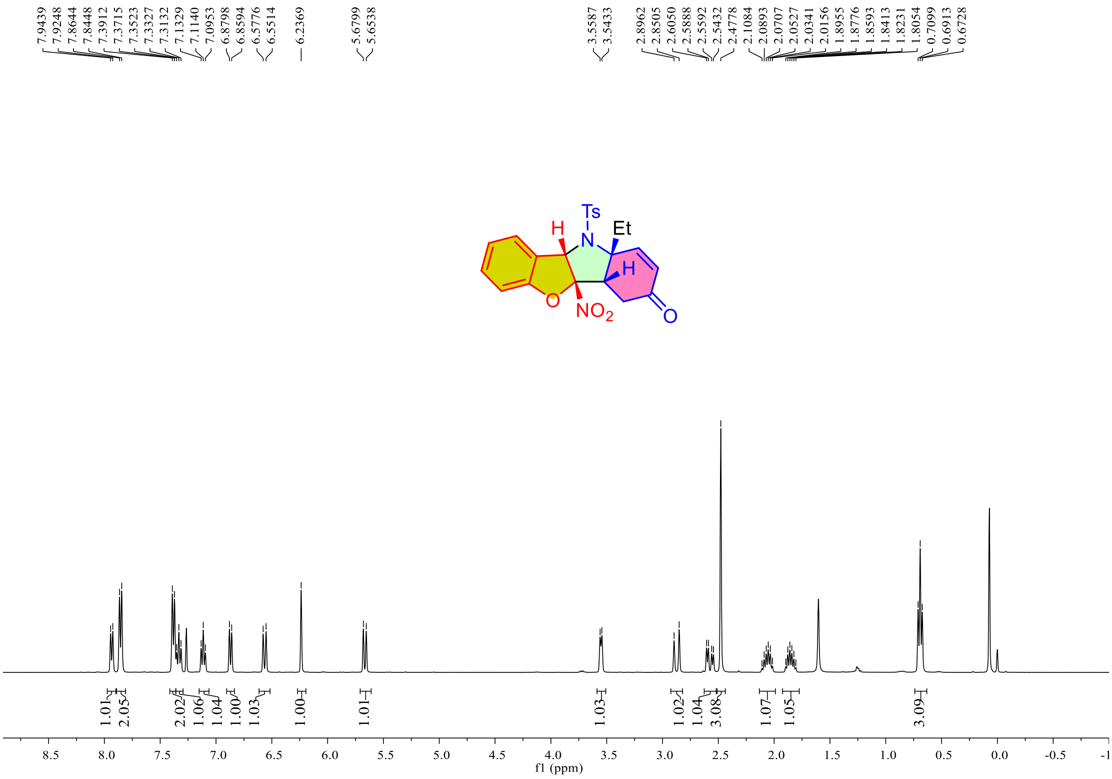

^{13}C{^{1}H} NMR (101 MHz, $CDCl_3$) of **3ba**

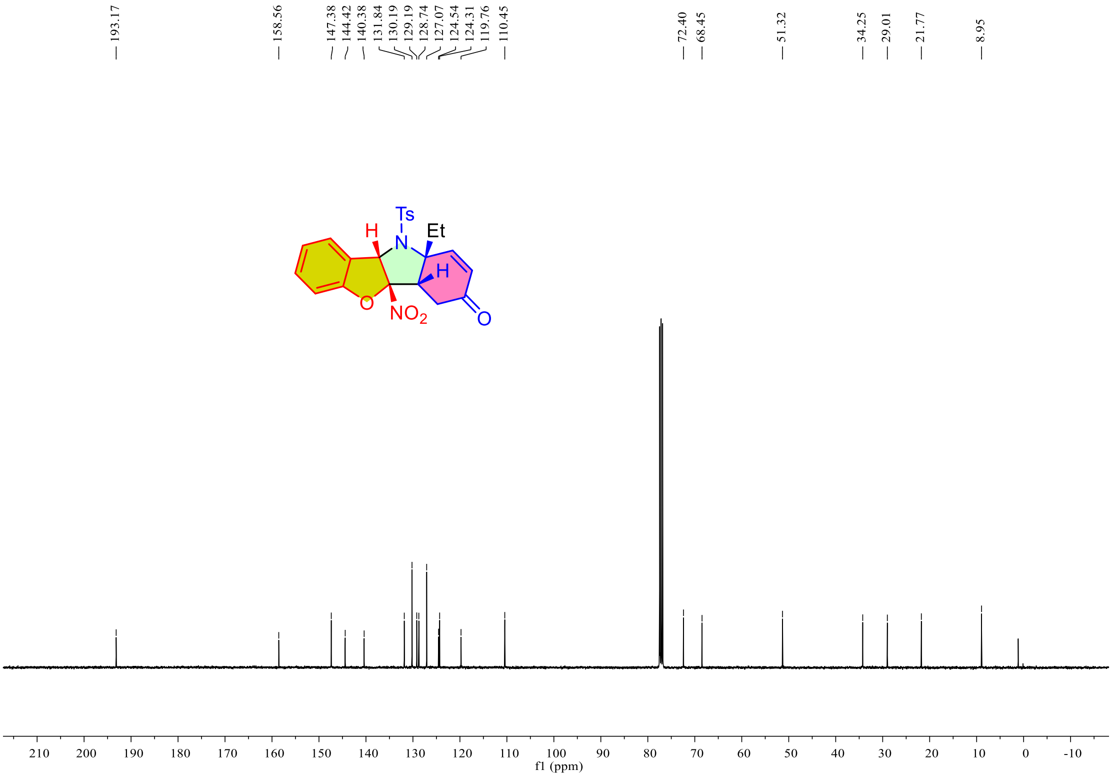

^{1}H NMR (400 MHz, $CDCl_3$) of **3ca**

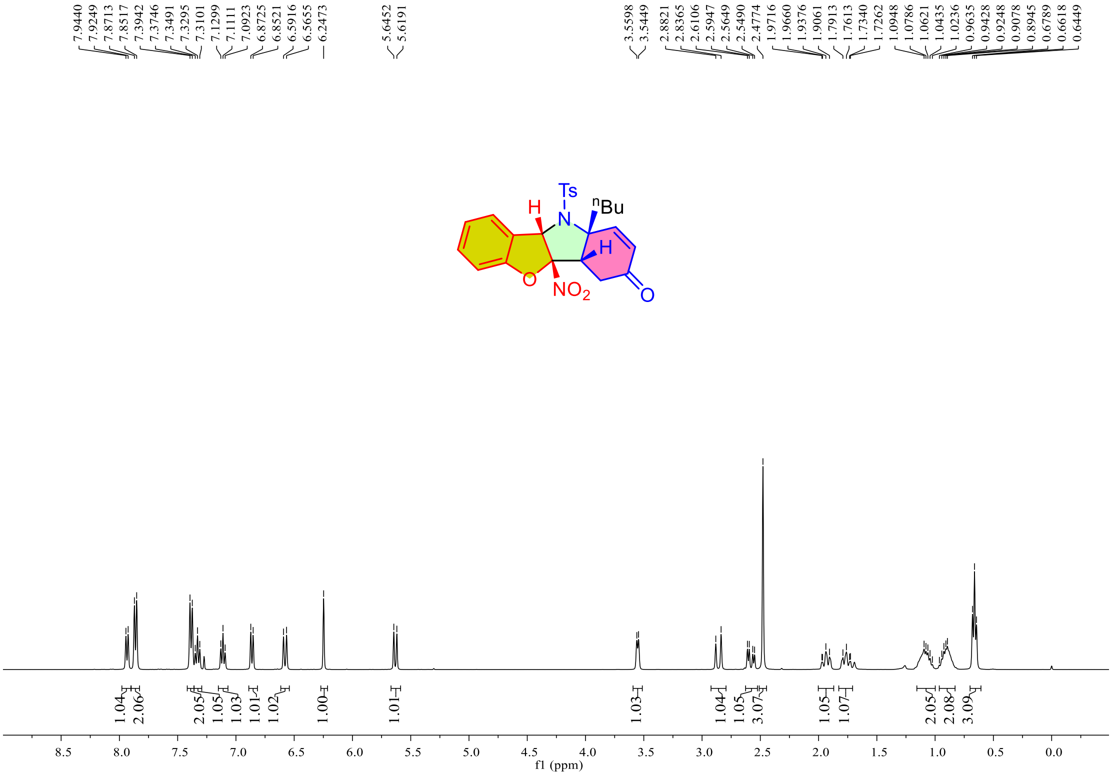

$^{13}C\{^1H\}$ NMR (101 MHz, $CDCl_3$) of **3ca**

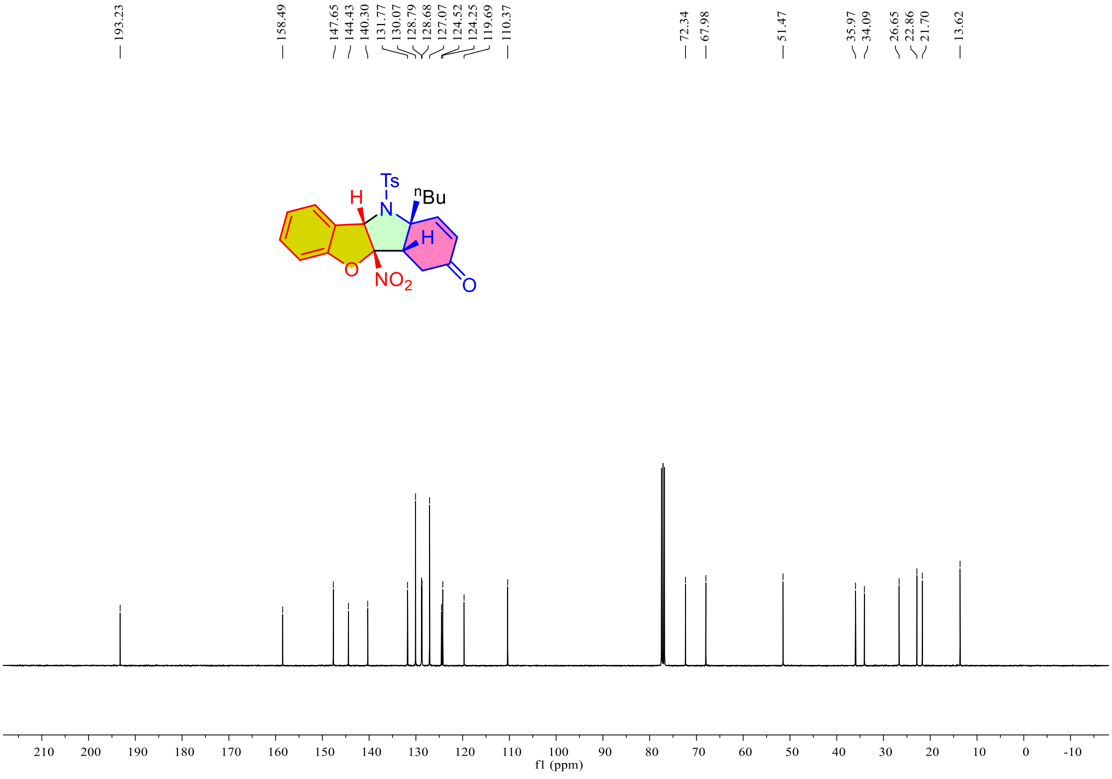

^{1}H NMR (400 MHz, $CDCl_3$) of **3da**

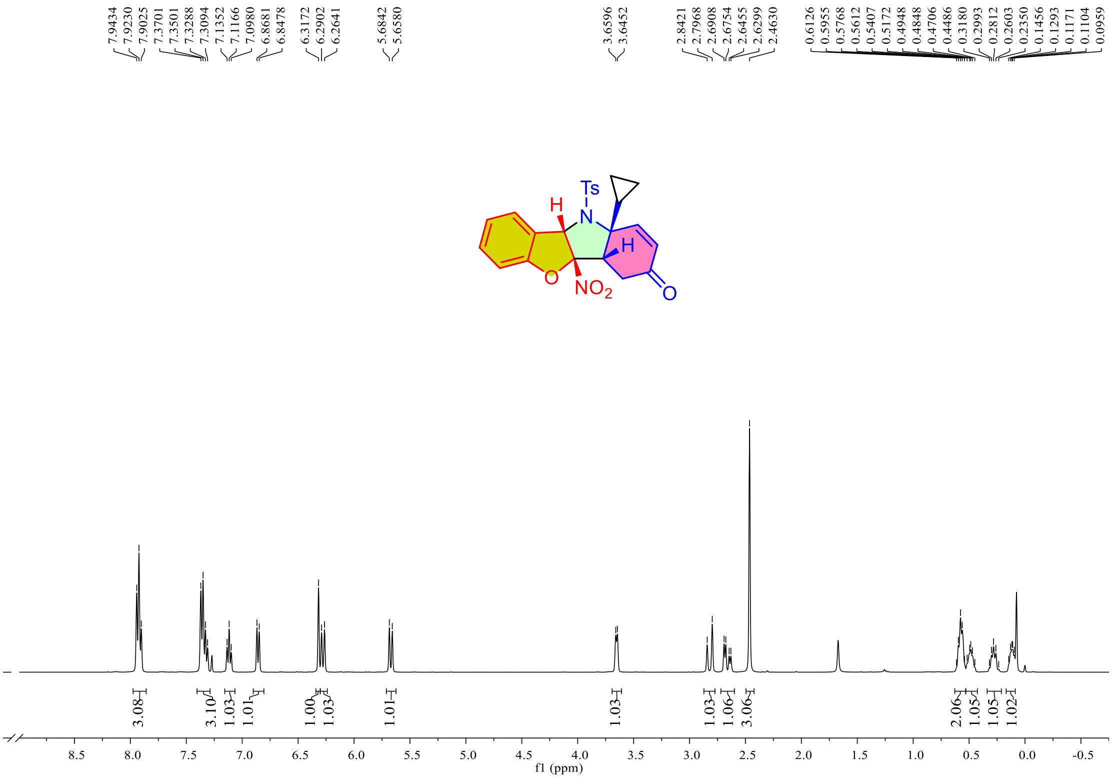

$^{13}C\{^{1}H\}$ NMR (101 MHz, $CDCl_3$) of **3da**

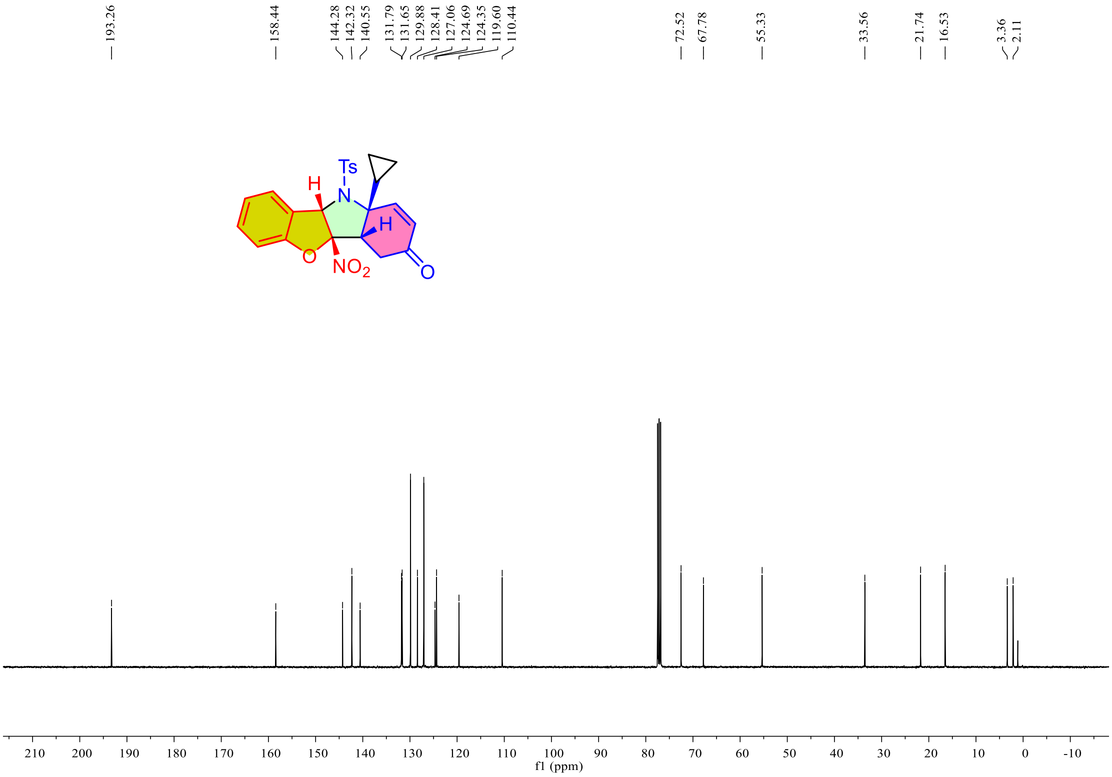

^{1}H NMR (400 MHz, $CDCl_3$) of **3ea**

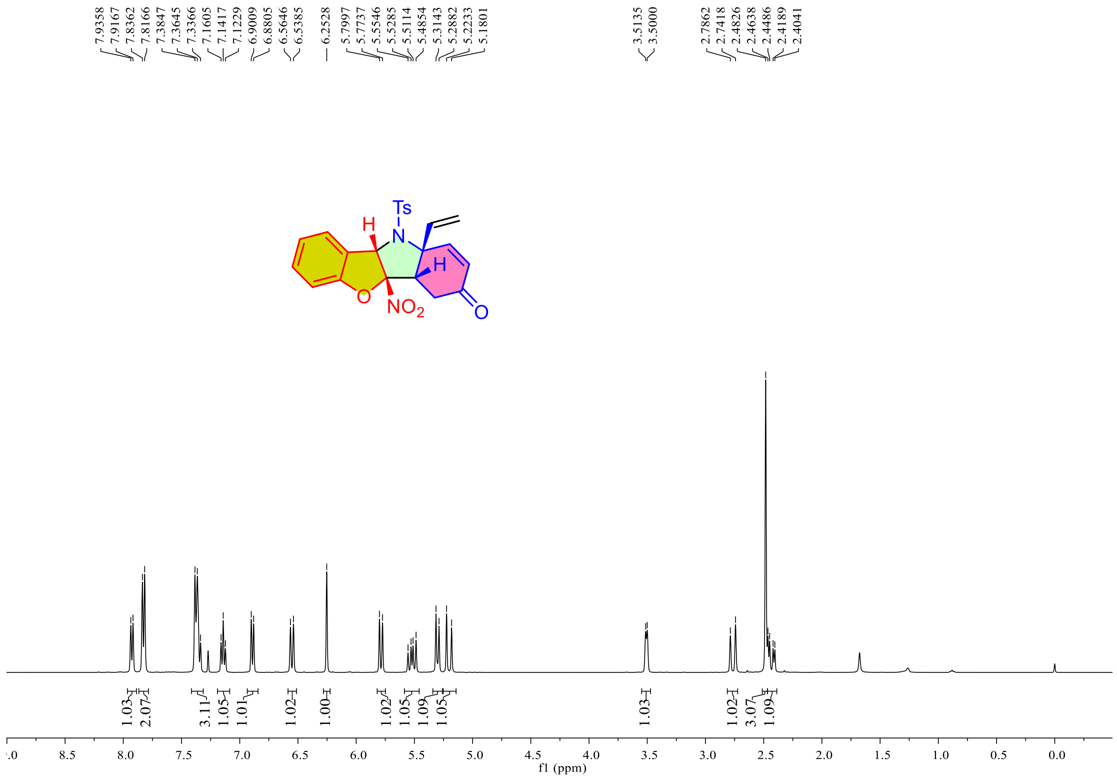

$^{13}C\{^{1}H\}$ NMR (101 MHz, $CDCl_3$) of **3ea**

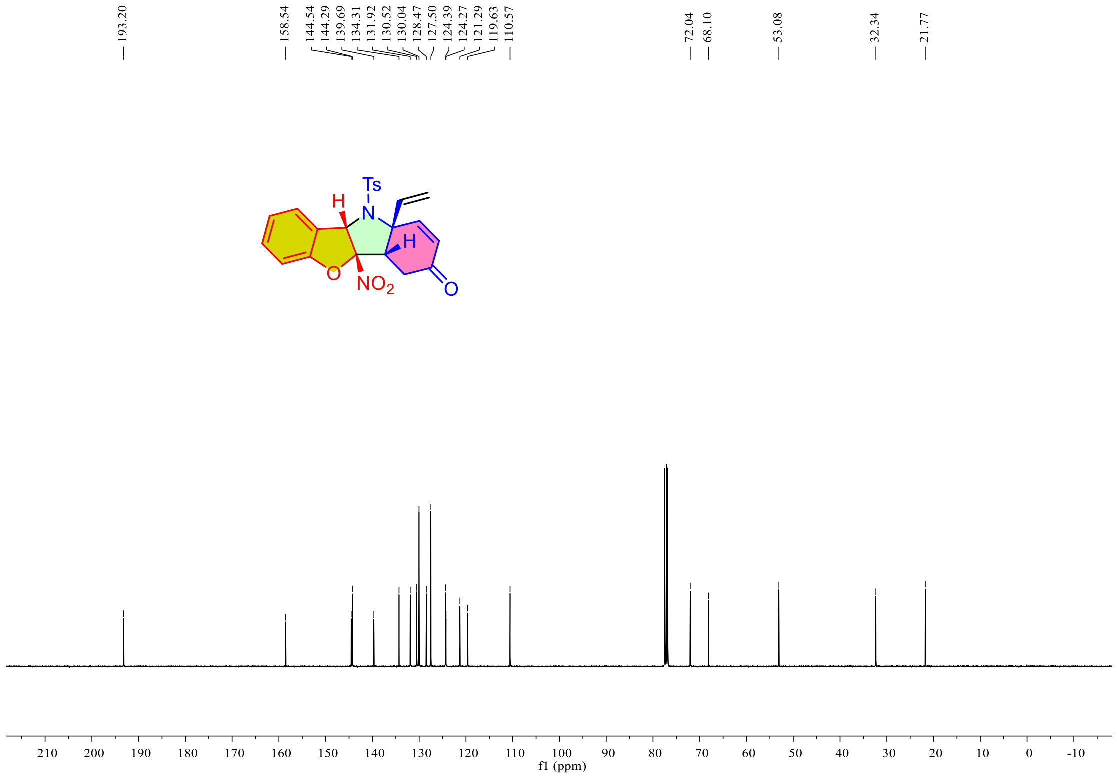

^{1}H NMR (400 MHz, $CDCl_3$) of **3fa**

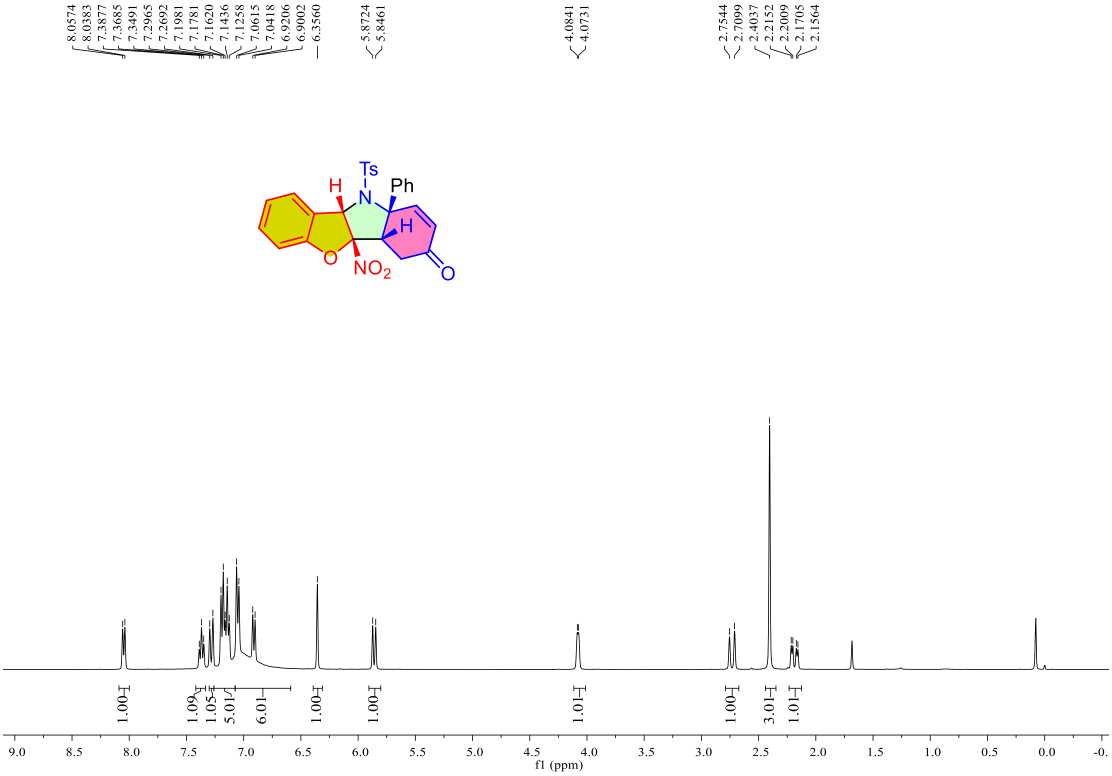

^{13}C{^{1}H} NMR (101 MHz, $CDCl_3$) of **3fa**

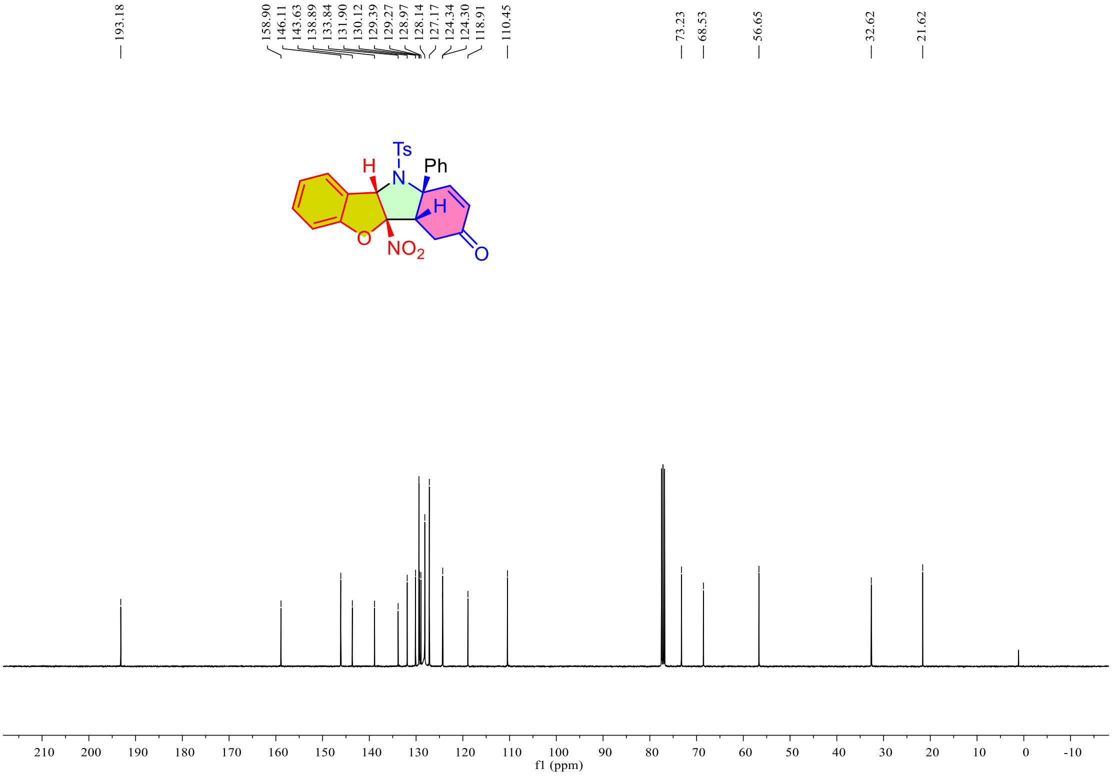

^{1}H NMR (400 MHz, $CDCl_3$) of **3ga**

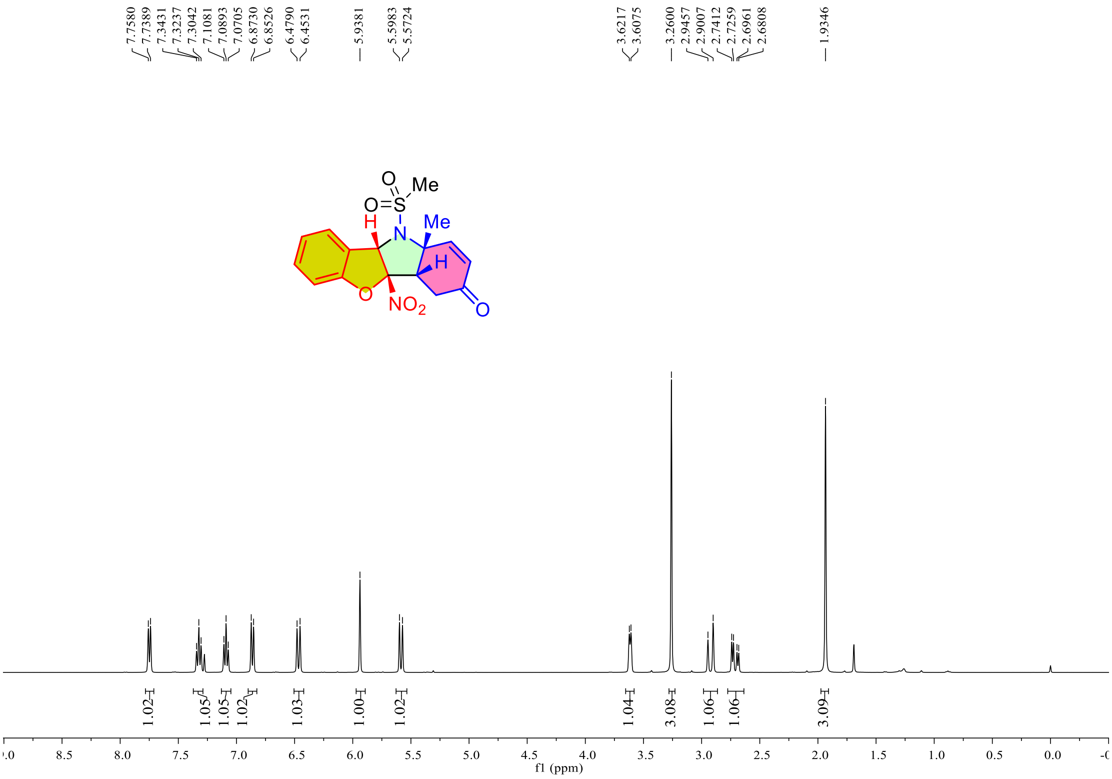

$^{13}C\{^{1}H\}$ NMR (101 MHz, $CDCl_3$) of **3ga**

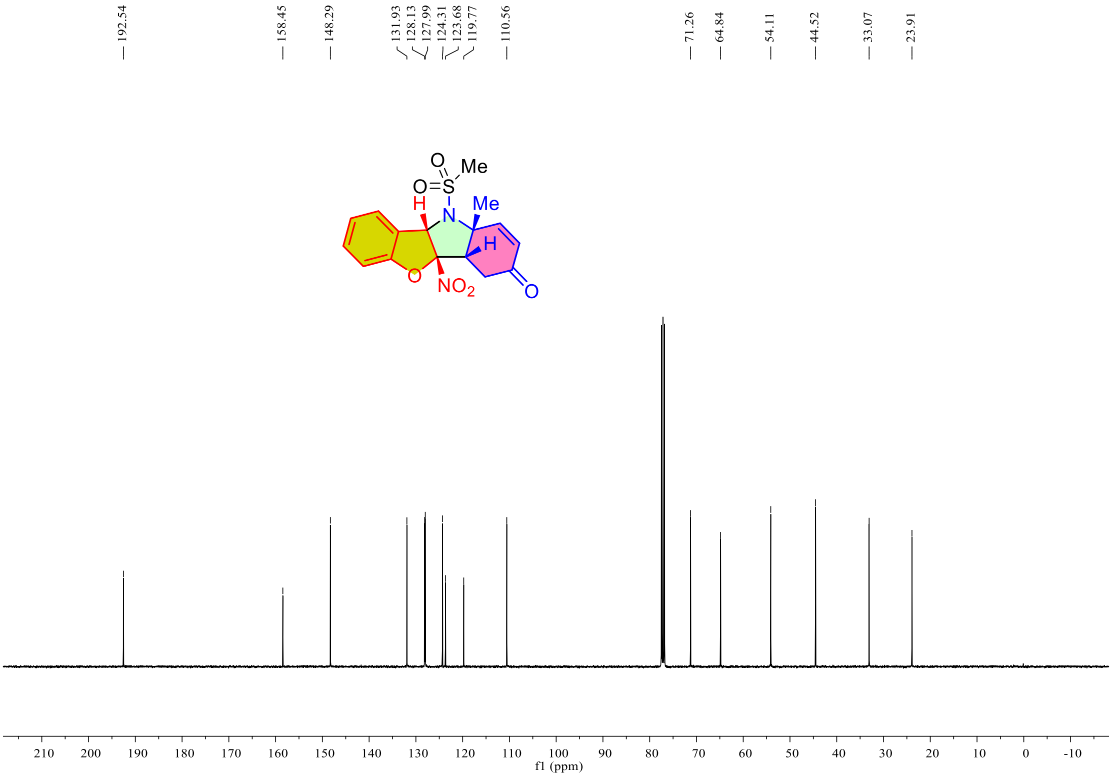

^{1}H NMR (400 MHz, $CDCl_3$) of **3ha**

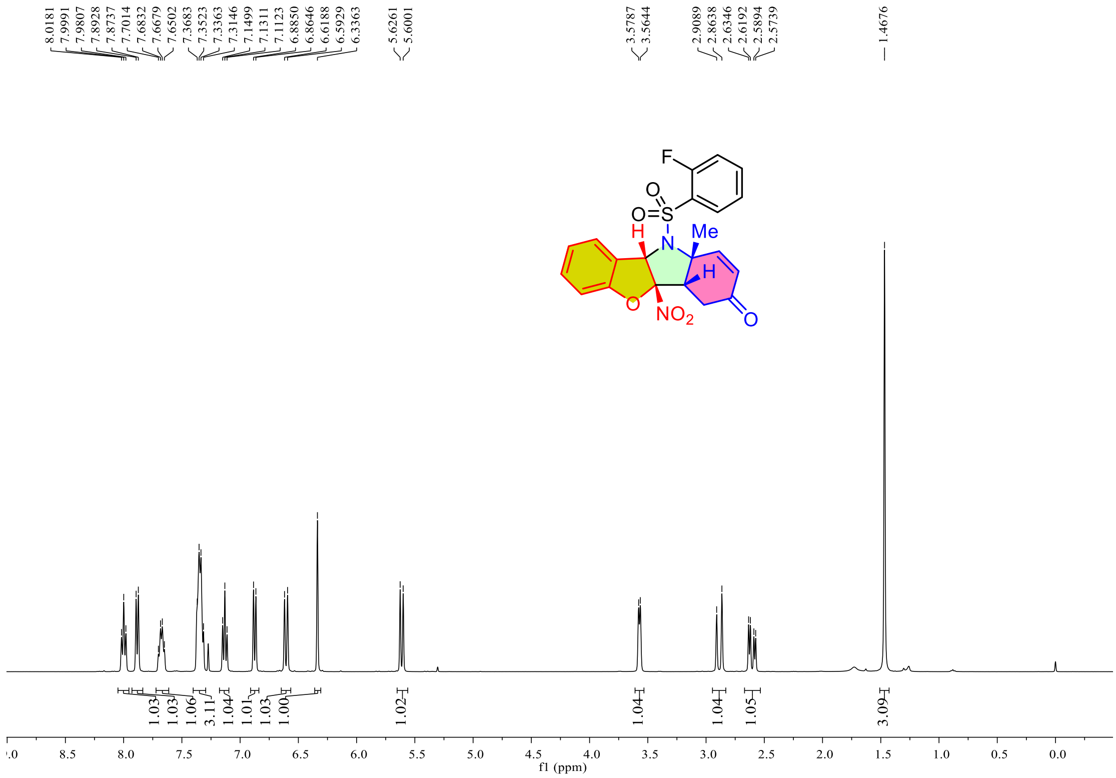

$^{13}C\{^{1}H\}$ NMR (101 MHz, $CDCl_3$) of **3ha**

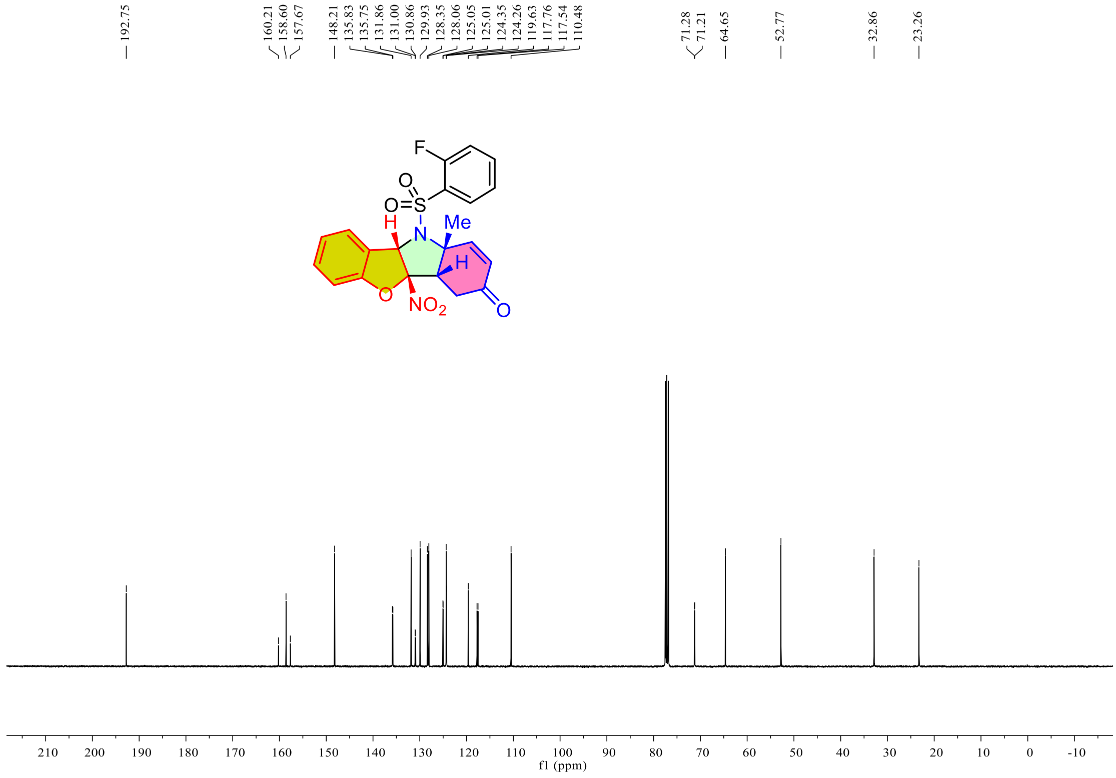

^{1}H NMR (400 MHz, $CDCl_3$) of **3ia**

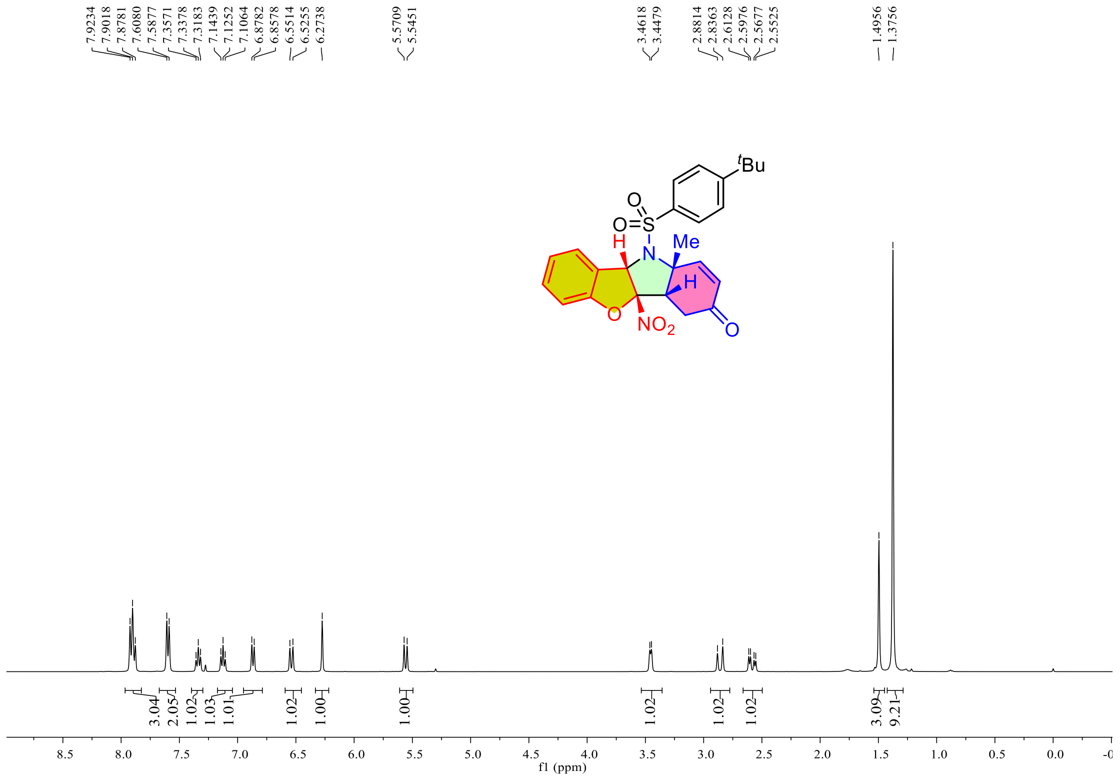

$^{13}C\{^{1}H\}$ NMR (101 MHz, $CDCl_3$) of **3ia**

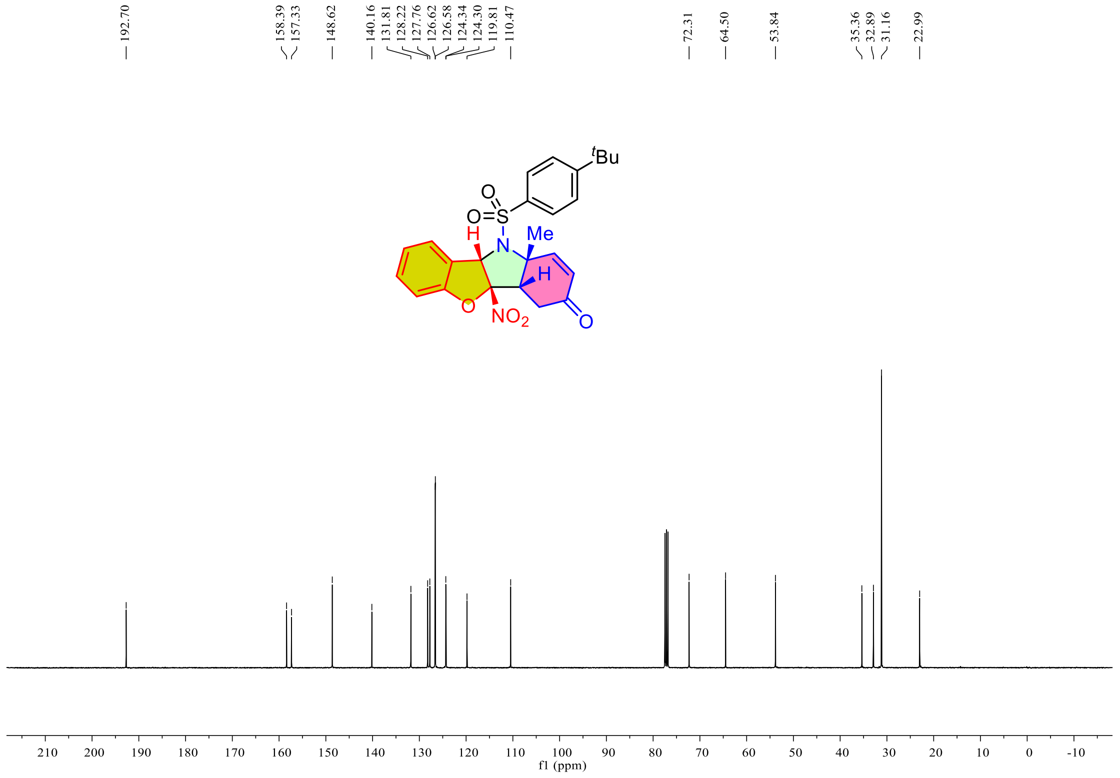

^{1}H NMR (400 MHz, $CDCl_3$) of **3ja**

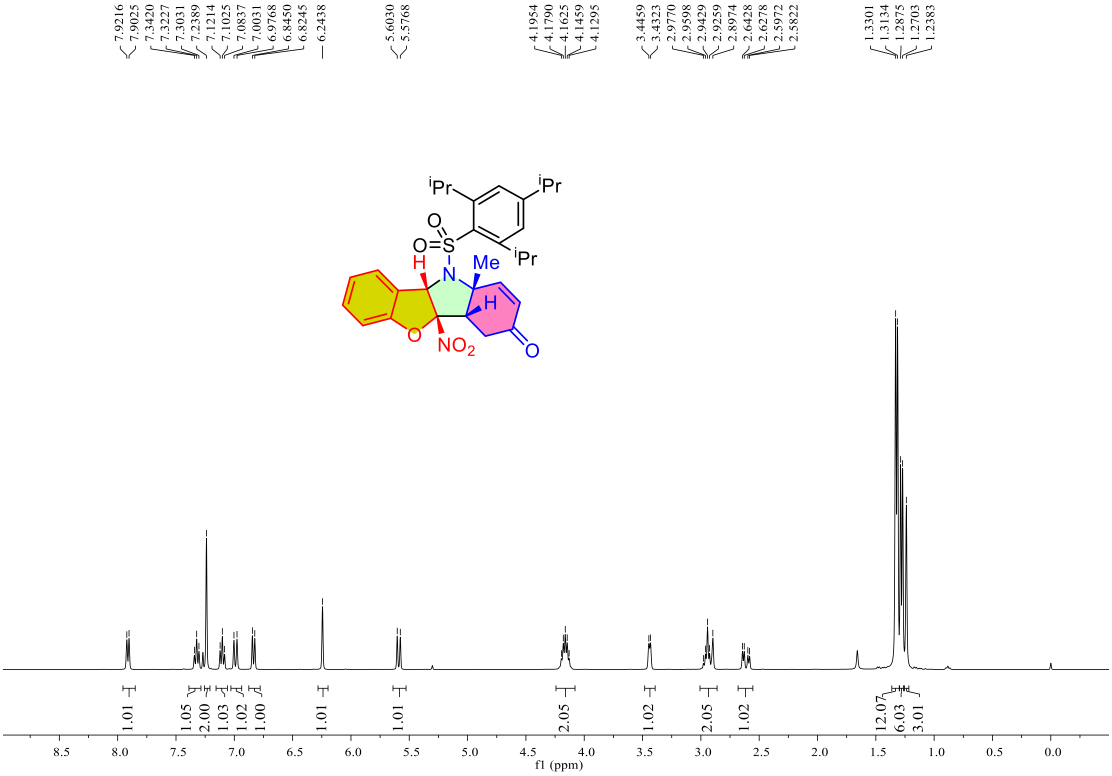

$^{13}C\{^{1}H\}$ NMR (101 MHz, $CDCl_3$) of **3ja**

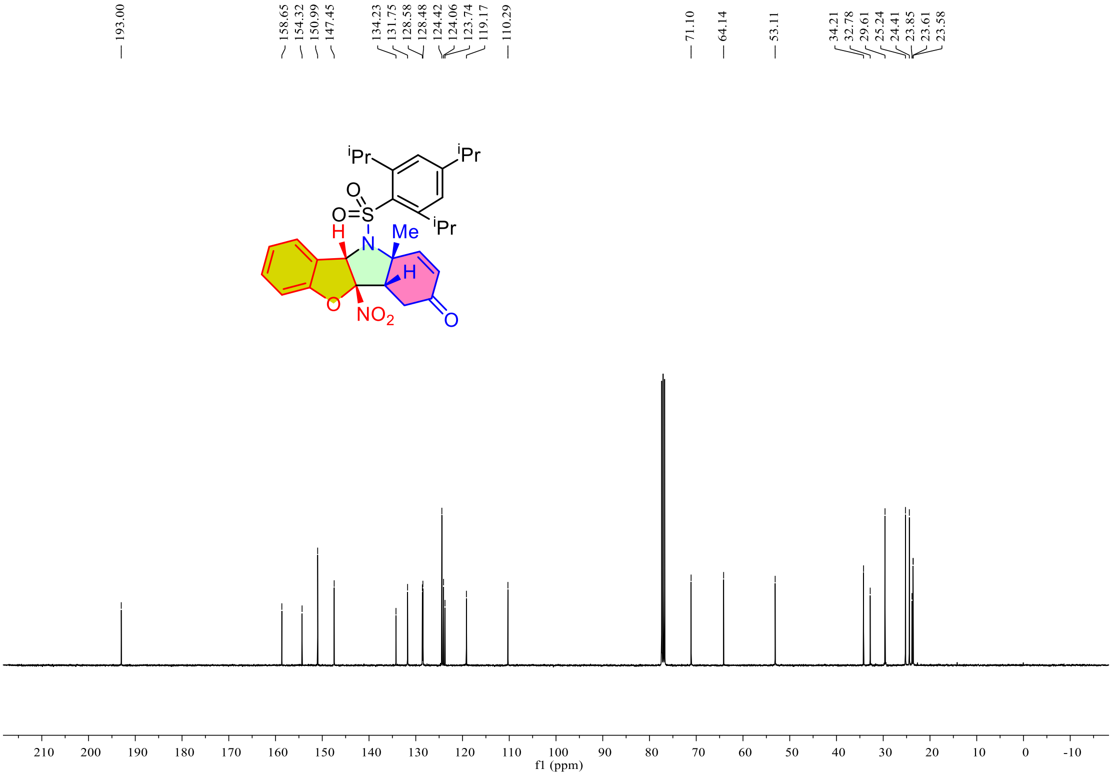

^{1}H NMR (400 MHz, $CDCl_3$) of **3ka**

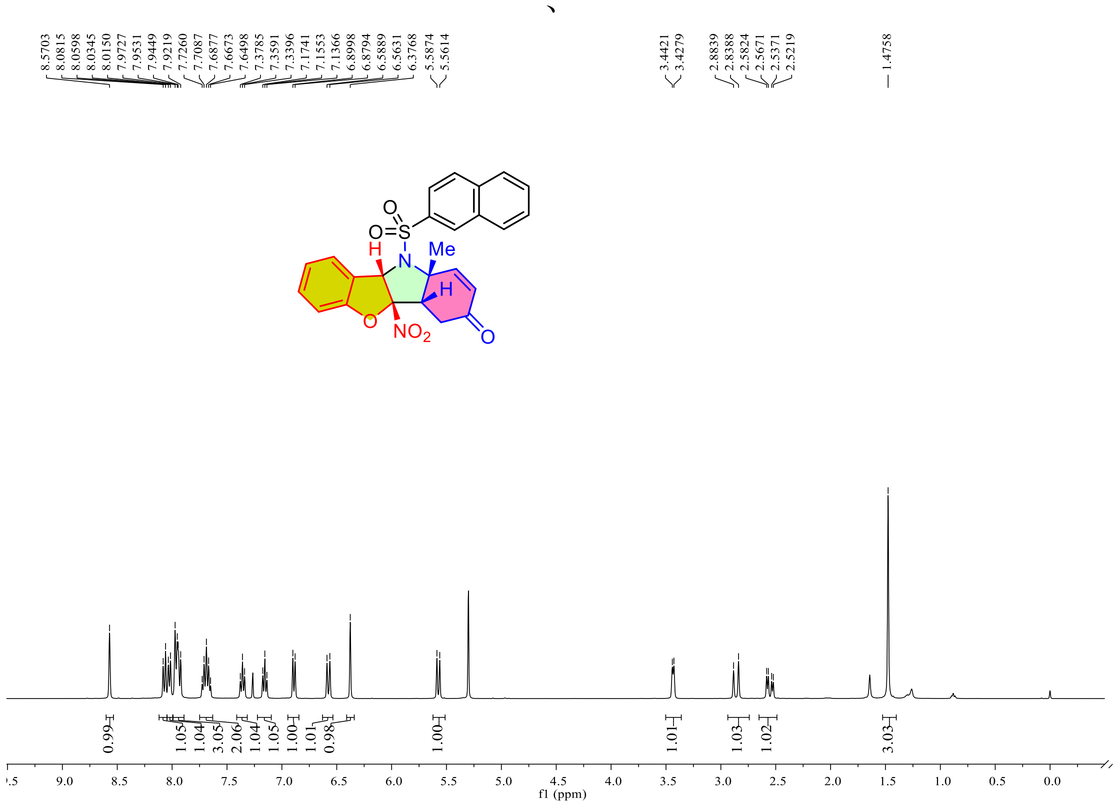

$^{13}C\{^{1}H\}$ NMR (101 MHz, $CDCl_3$) of **3ka**

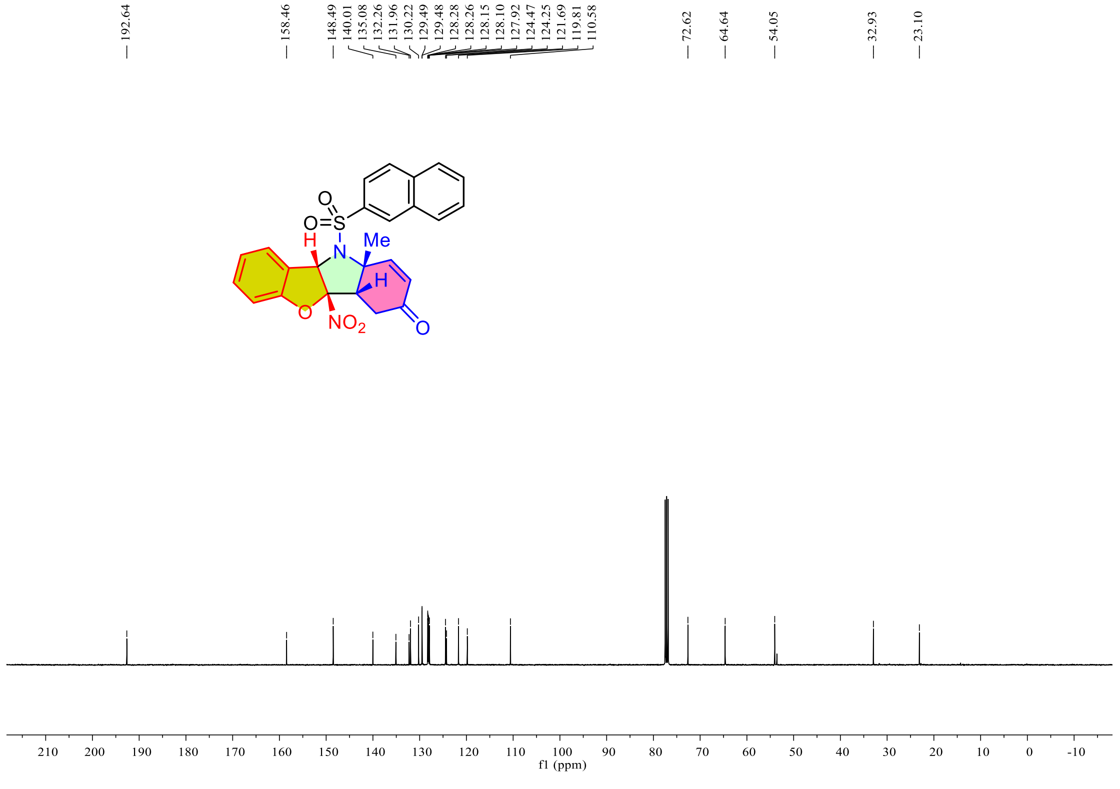

^1H NMR (400 MHz, $CDCl_3$) of **3la**

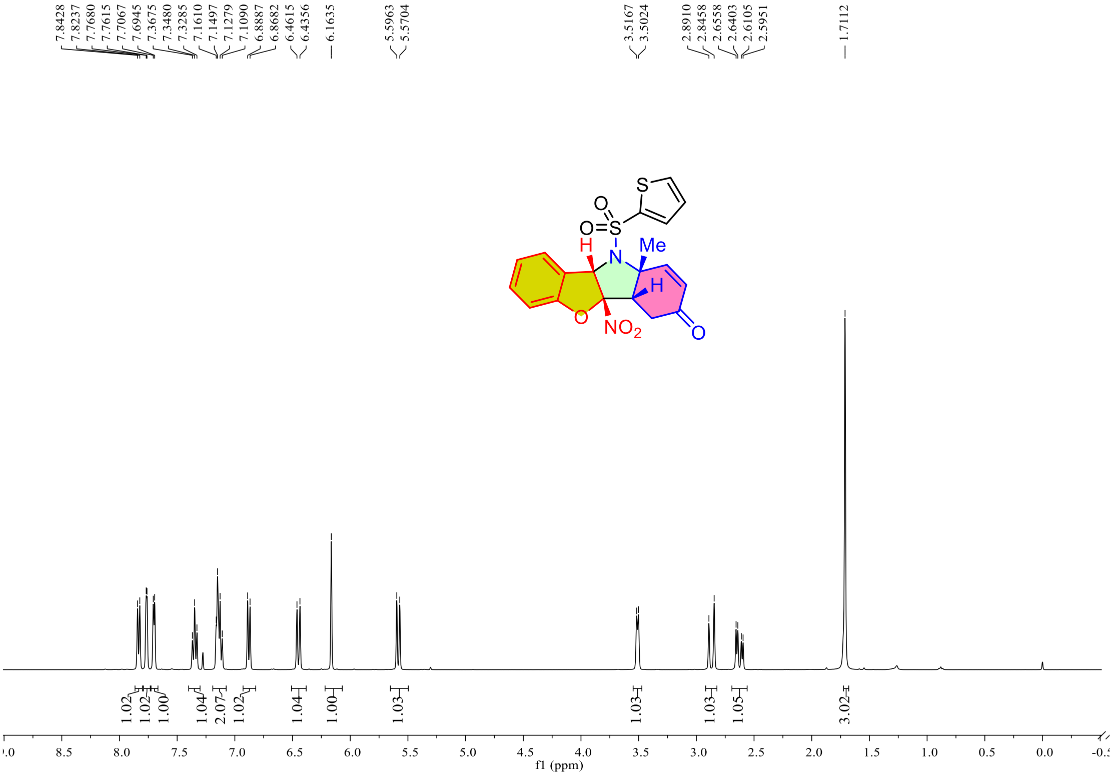

$^{13}C\{^1H\}$ NMR (101 MHz, $CDCl_3$) of **3la**

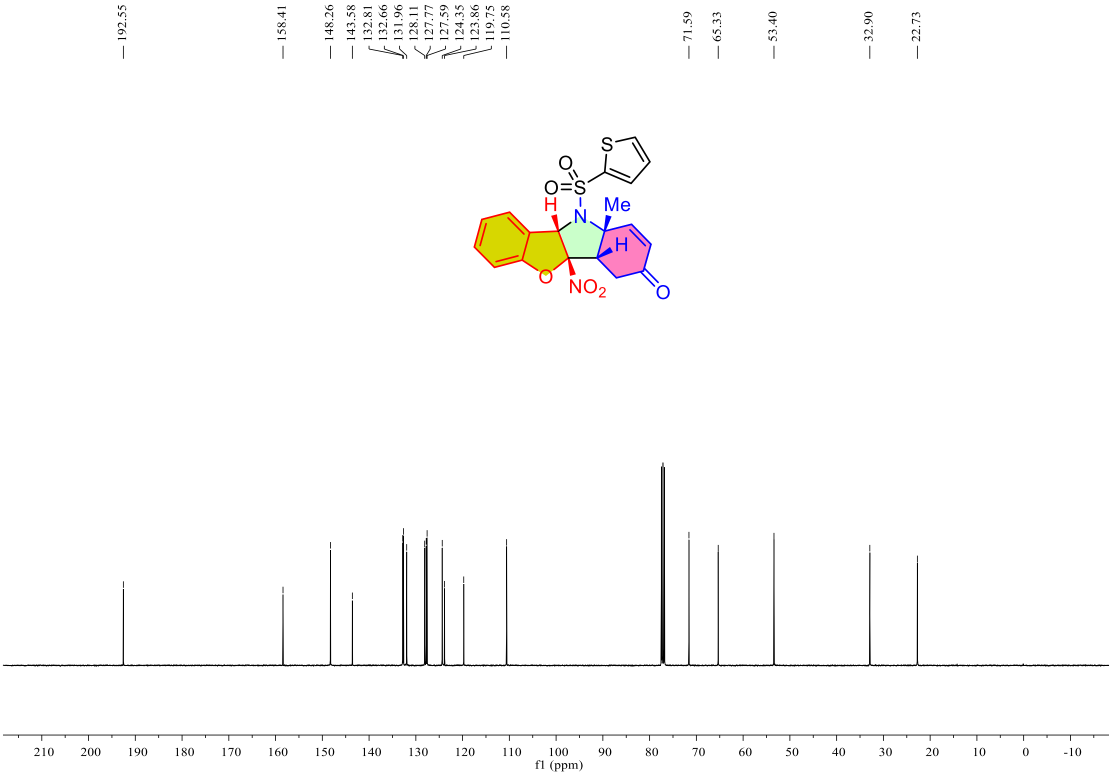

^{1}H NMR (400 MHz, DMSO-d_6) of **3ab**

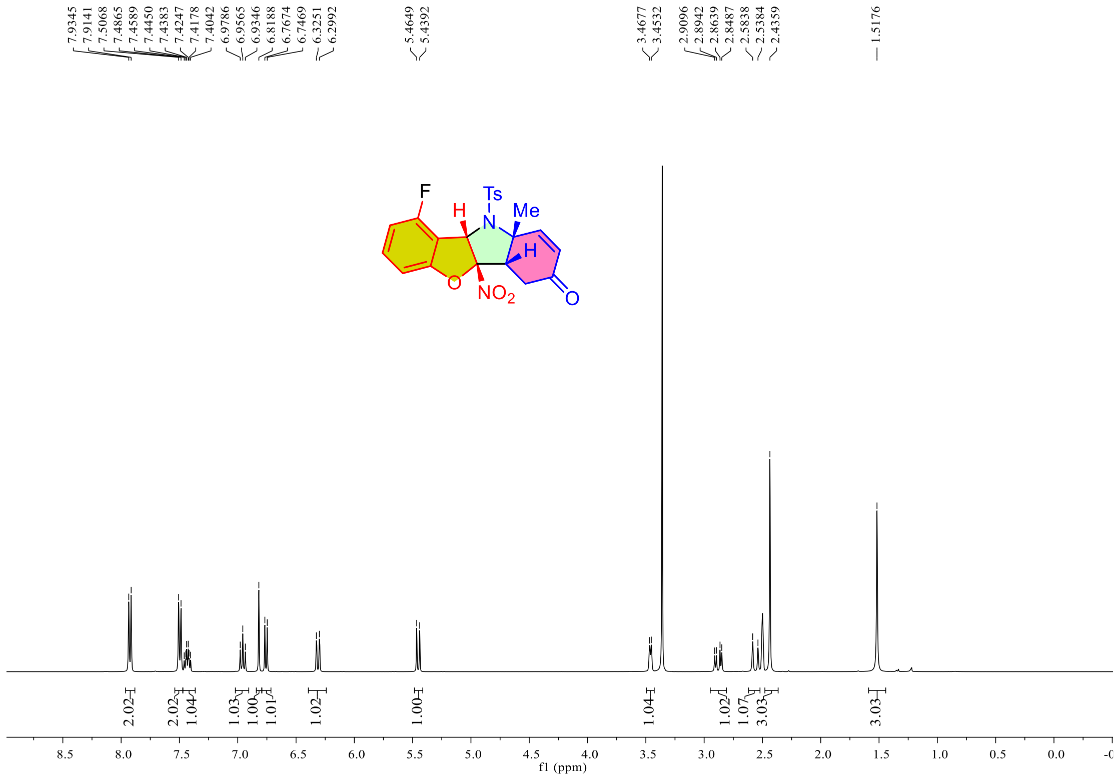

^{13}C{^{1}H} NMR (101 MHz, DMSO-d_6) of **3ab**

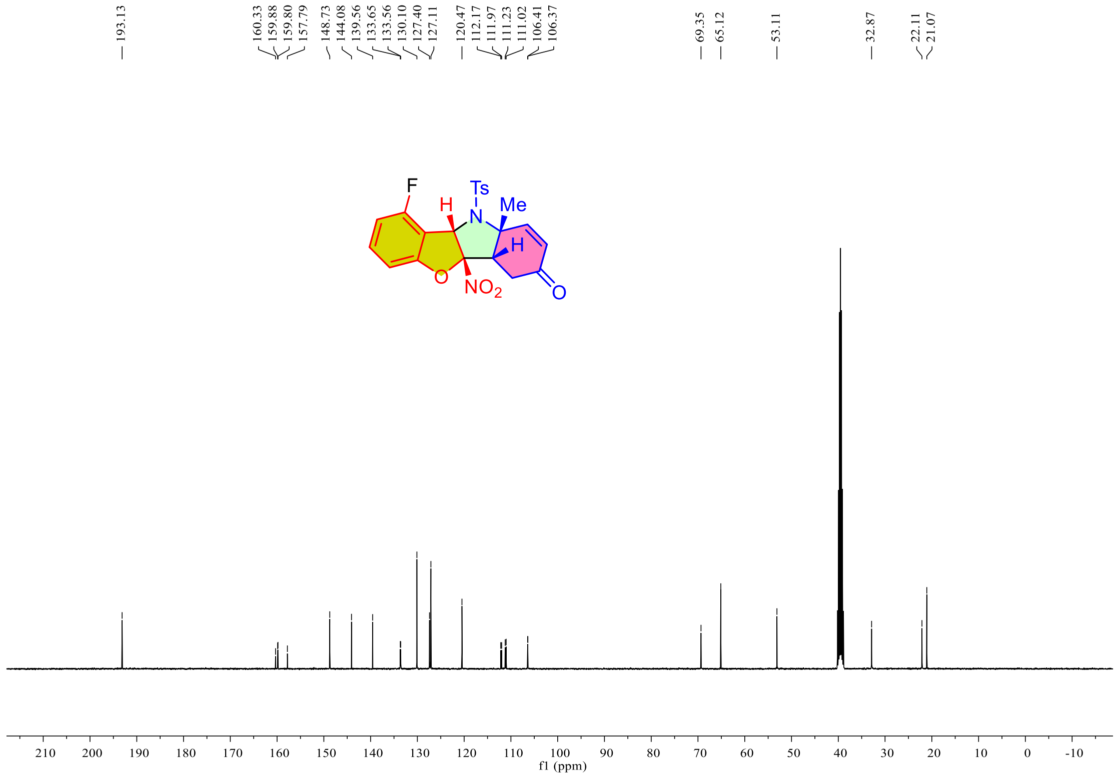

^{1}H NMR (400 MHz, $CDCl_3$) of **3ac**

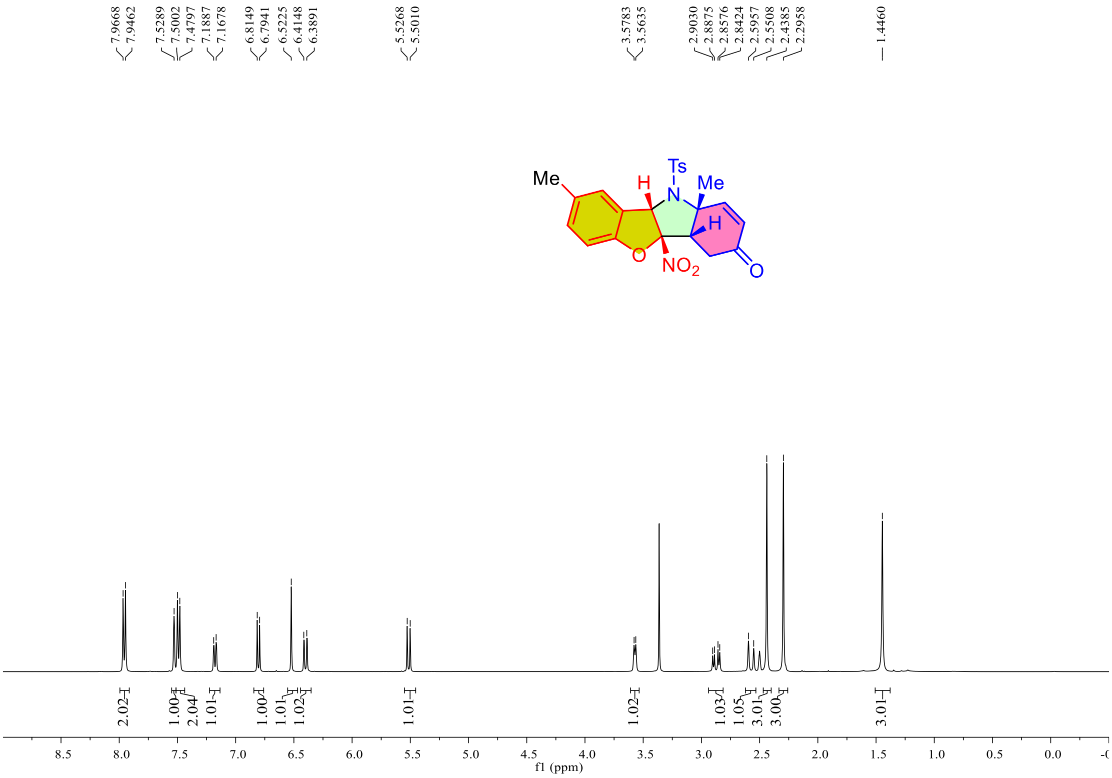

$^{13}C\{^1H\}$ NMR (101 MHz, $CDCl_3$) of **3ac**

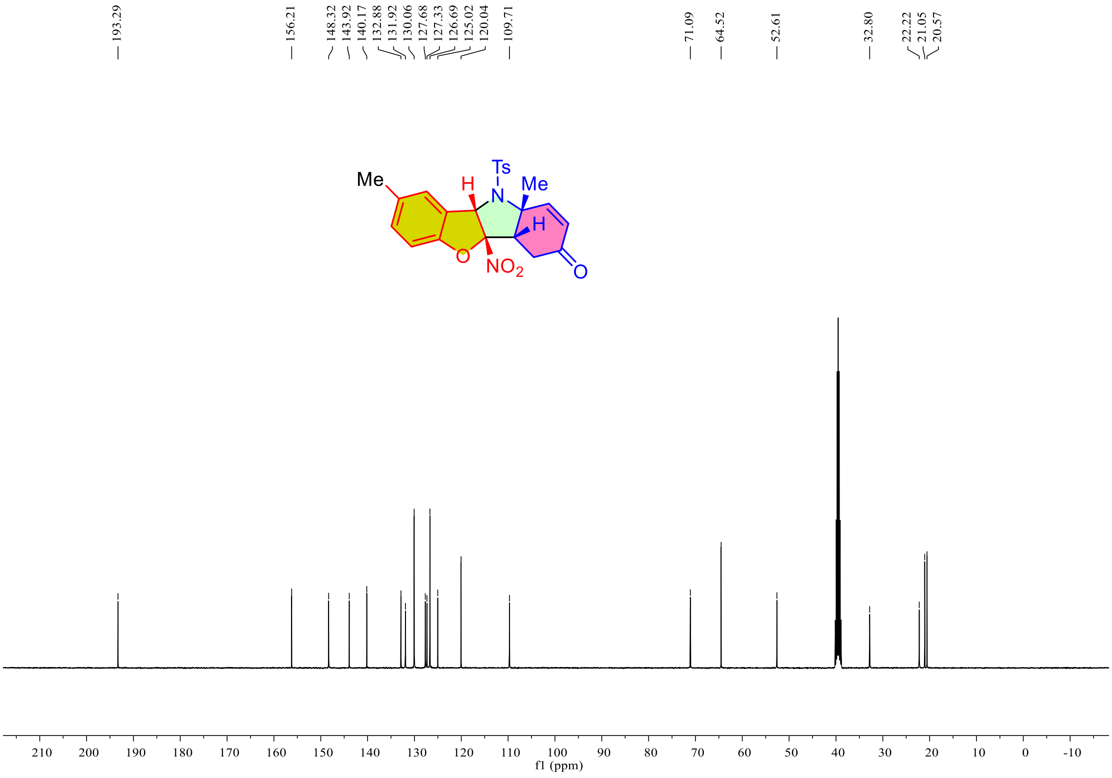

^{1}H NMR (400 MHz, CDCl$_3$) of **3ad**

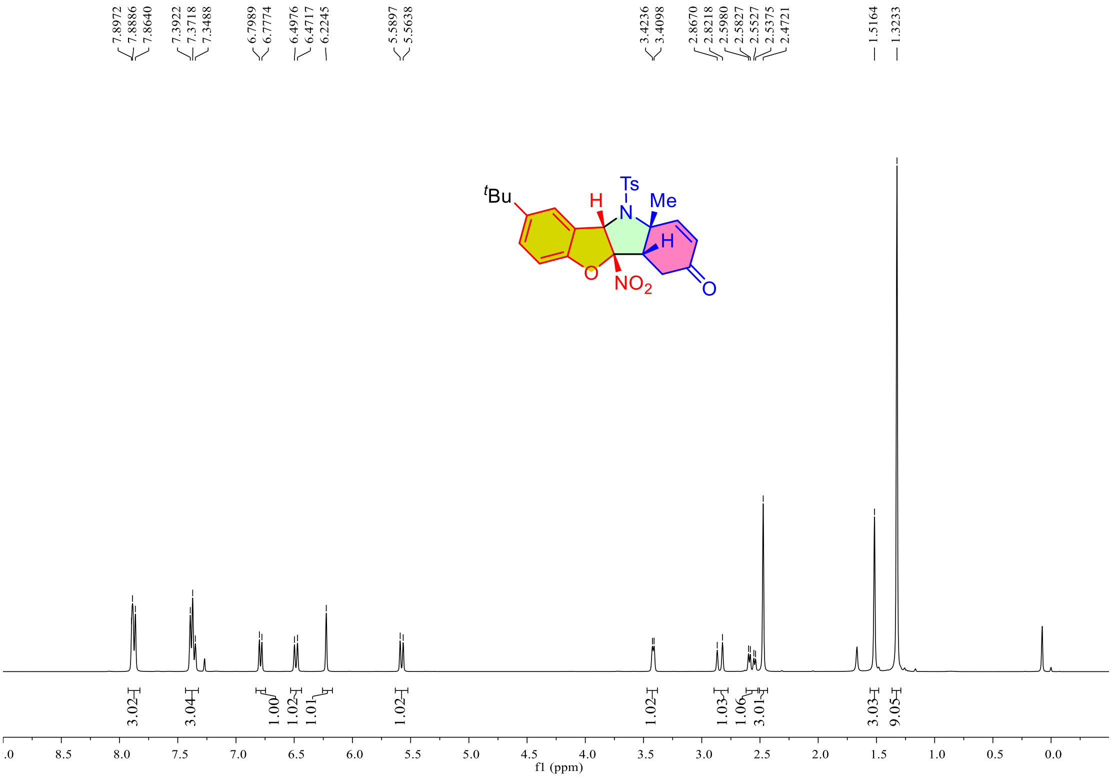

^{13}C{^{1}H} NMR (101 MHz, CDCl$_3$) of **3ad**

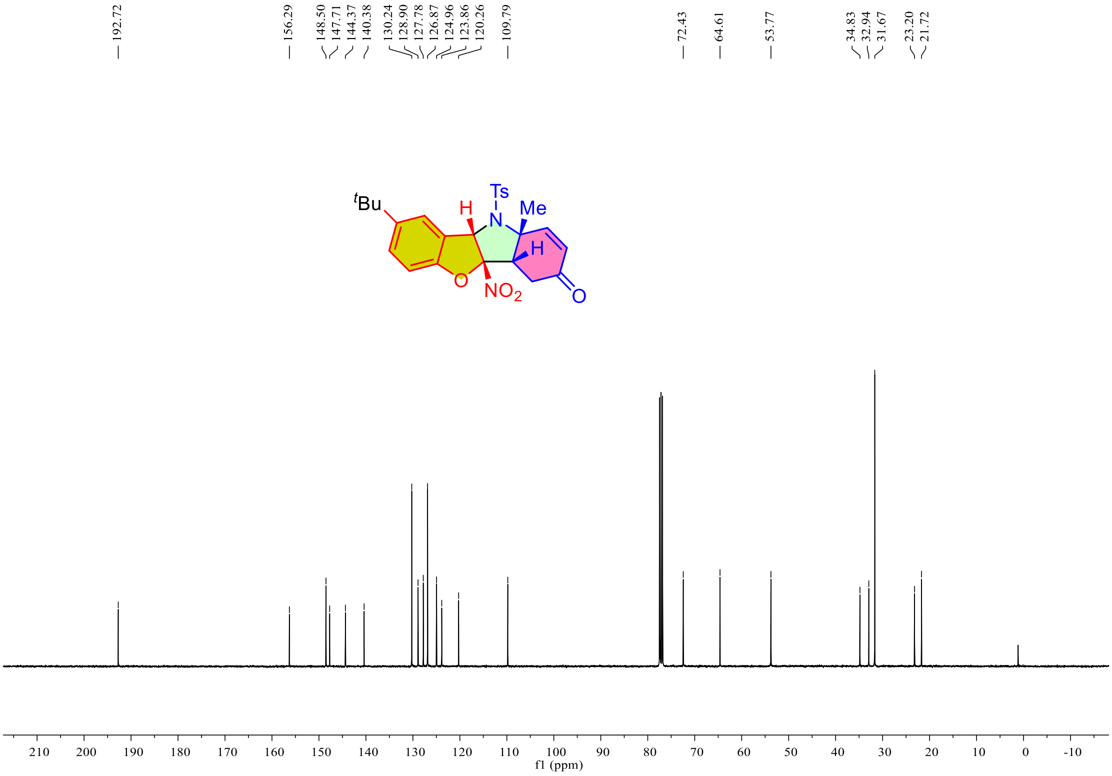

^{1}H NMR (400 MHz, CDCl$_3$) of **3ae**

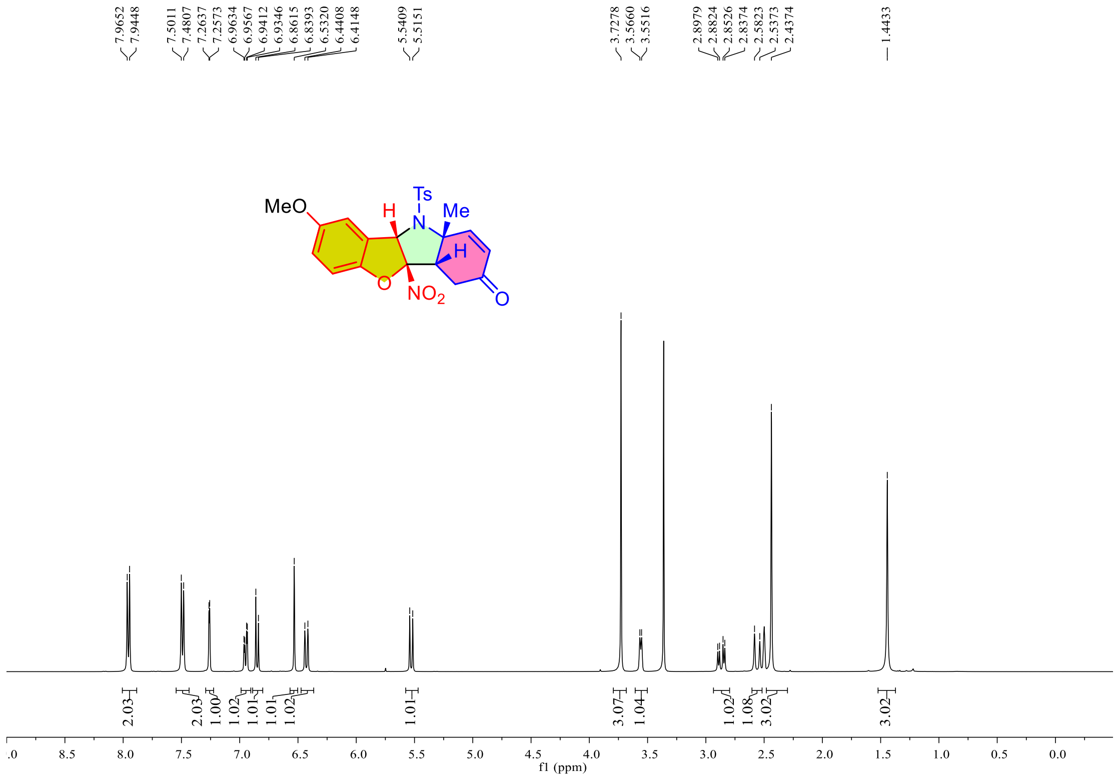

^{13}C{^{1}H} NMR (101 MHz, CDCl$_3$) of **3ae**

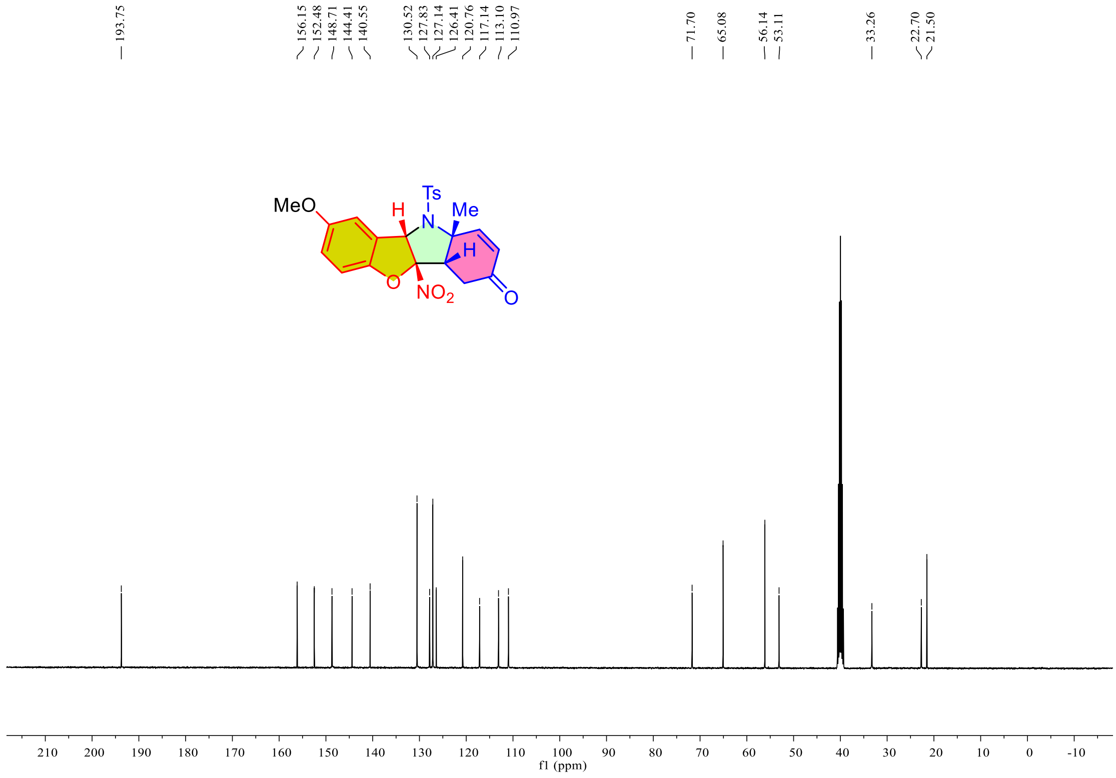

^{1}H NMR (400 MHz, DMSO-d_6) of **3af**

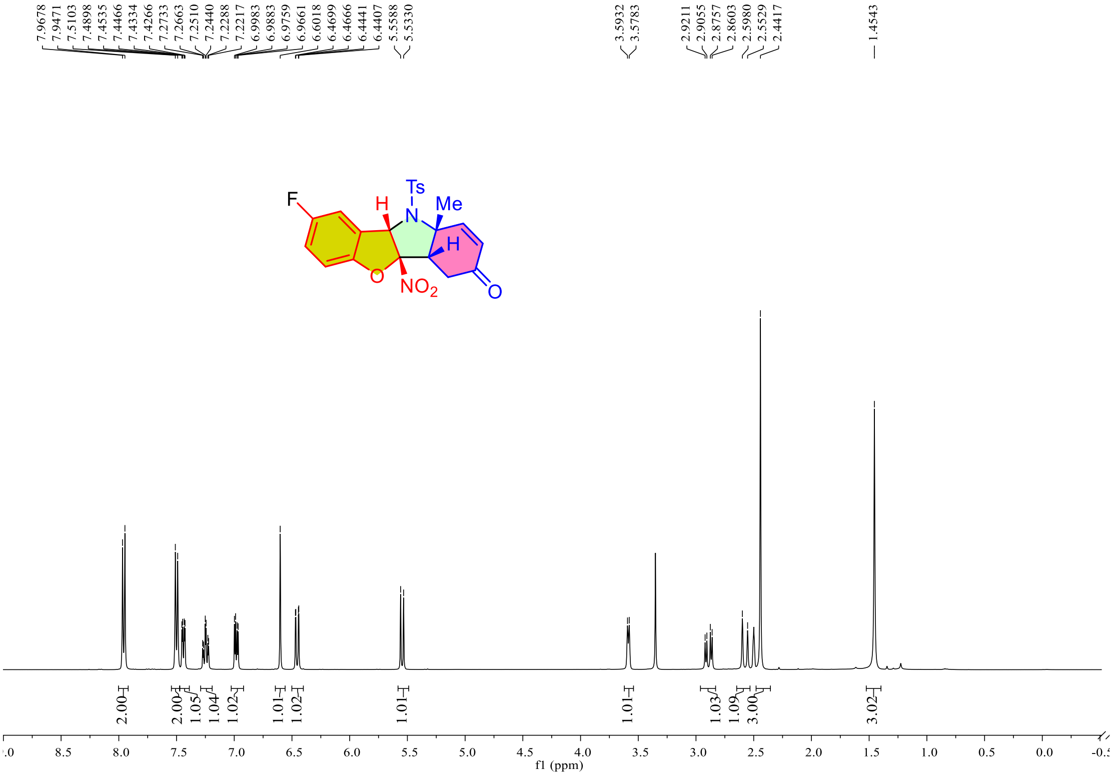

^{13}C{^{1}H} NMR (101 MHz, DMSO-d_6) of **3af**

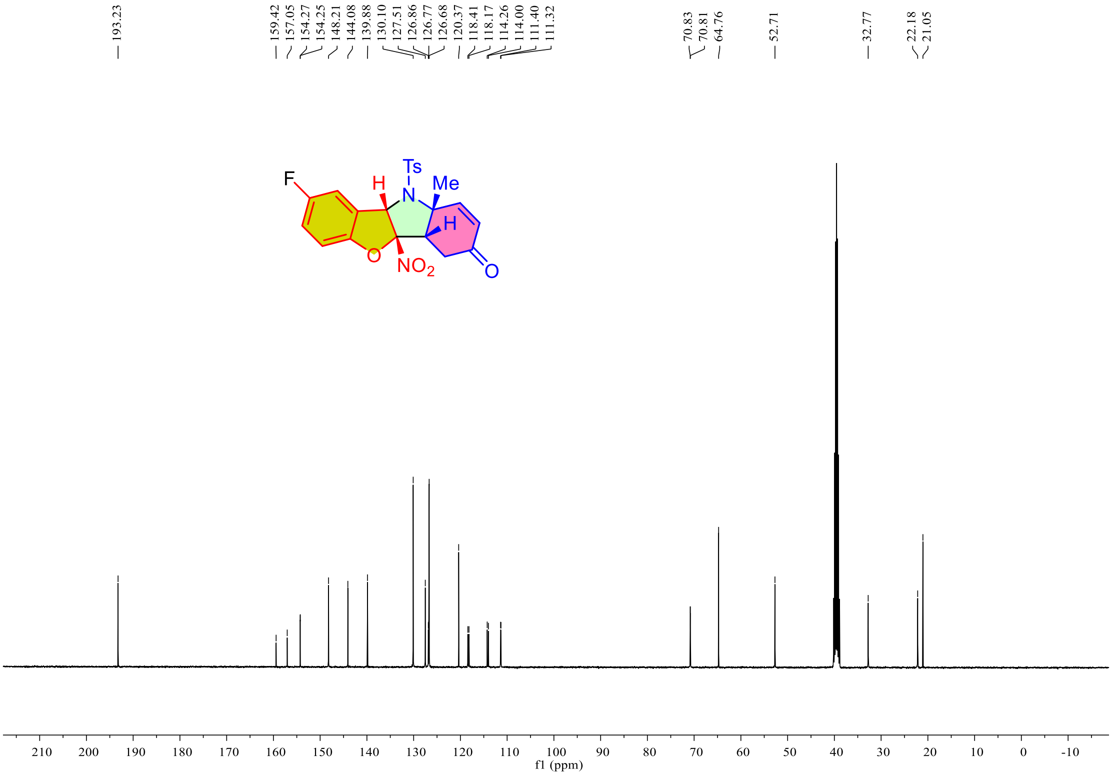

^{1}H NMR (400 MHz, $CDCl_3$) of **3ag**

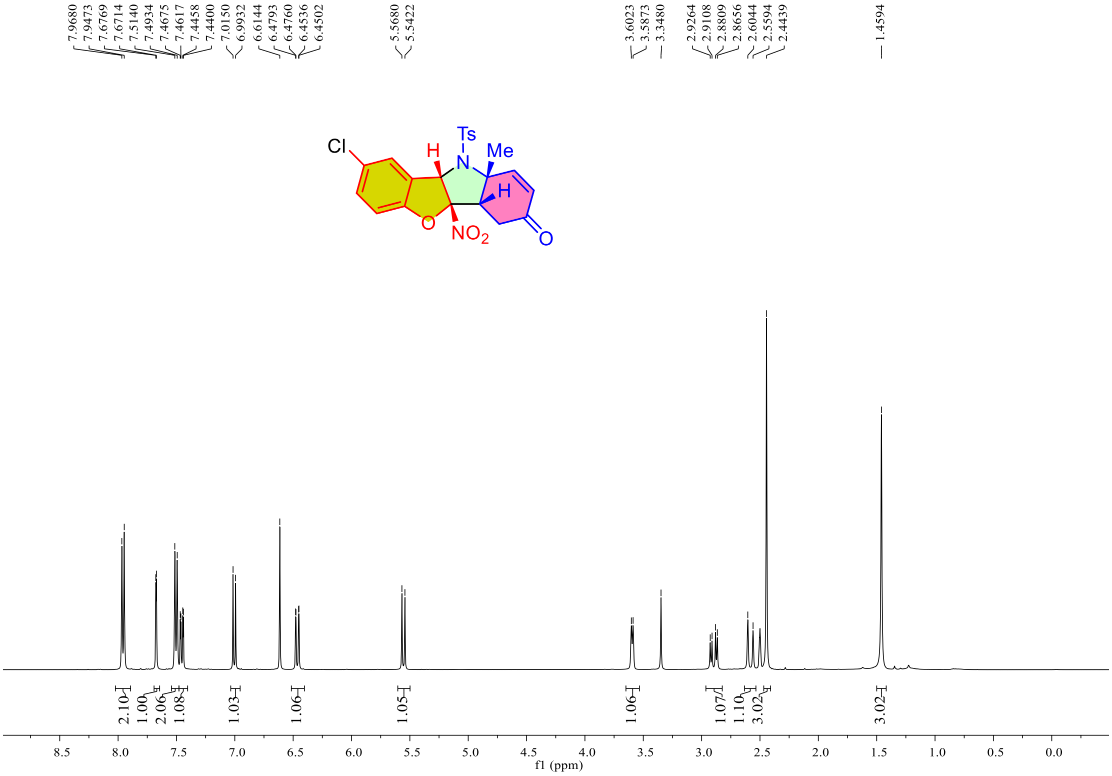

$^{13}C\{^{1}H\}$ NMR (101 MHz, $CDCl_3$) of **3ag**

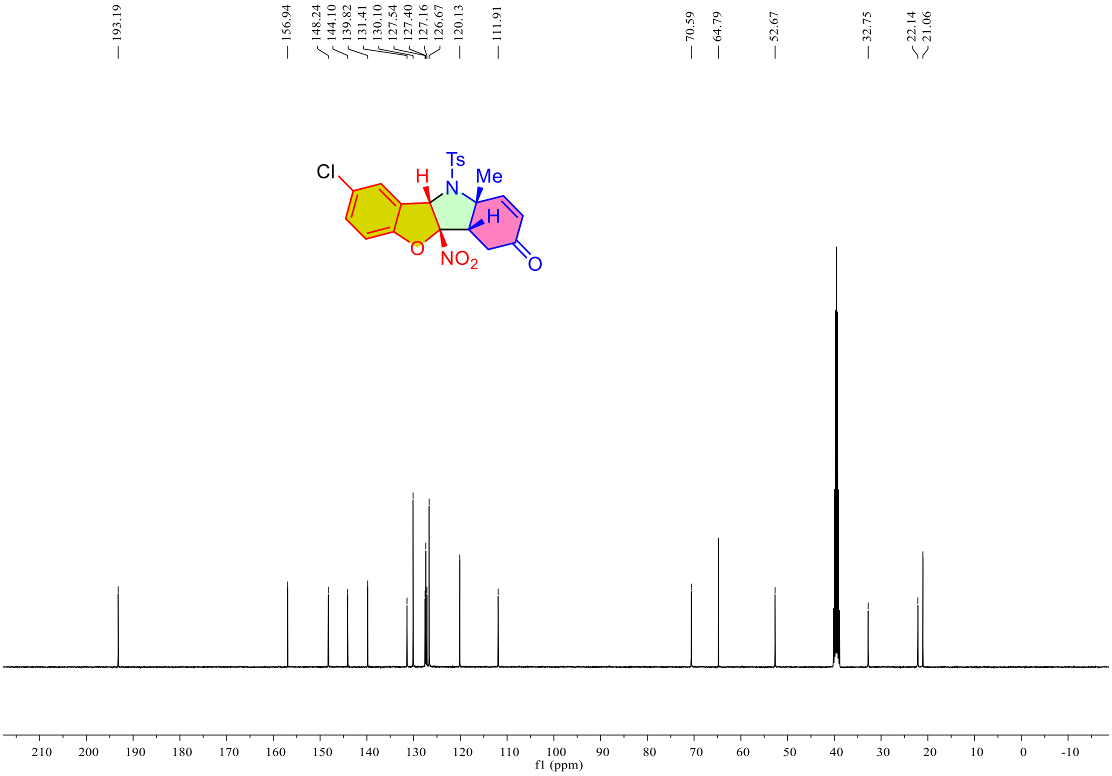

^{1}H NMR (400 MHz, DMSO-d_6) of **3ah**

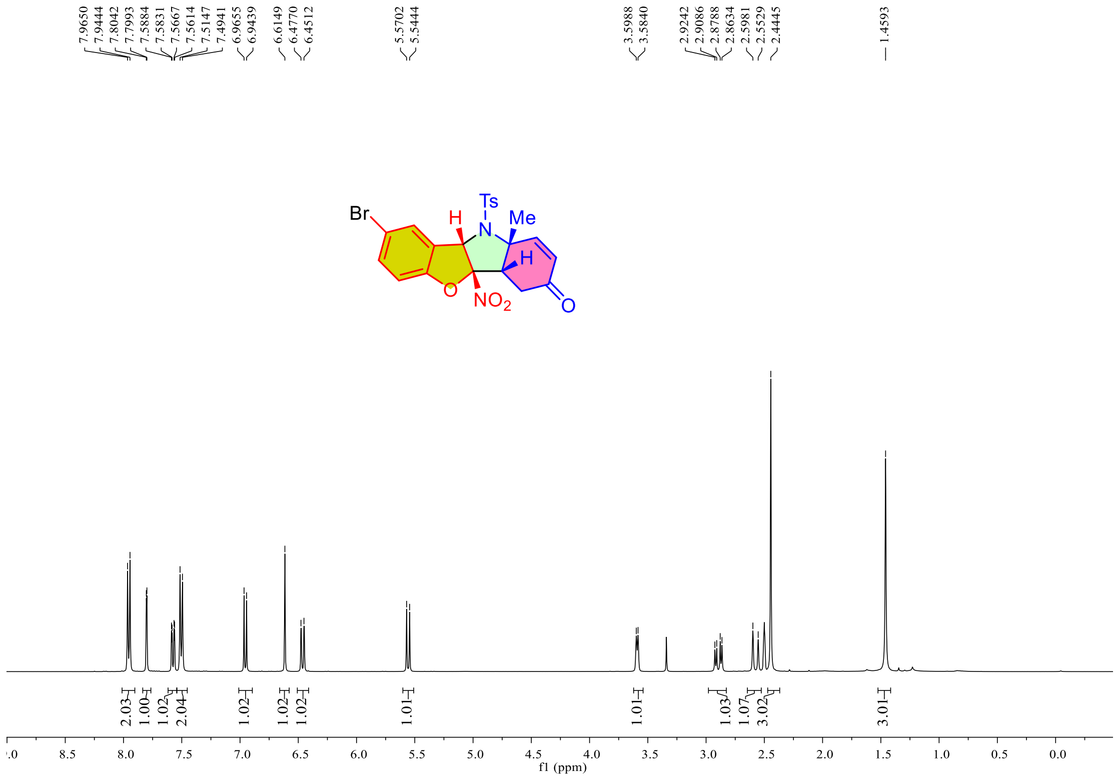

$^{13}C\{^{1}H\}$ NMR (101 MHz, DMSO-d_6) of **3ah**

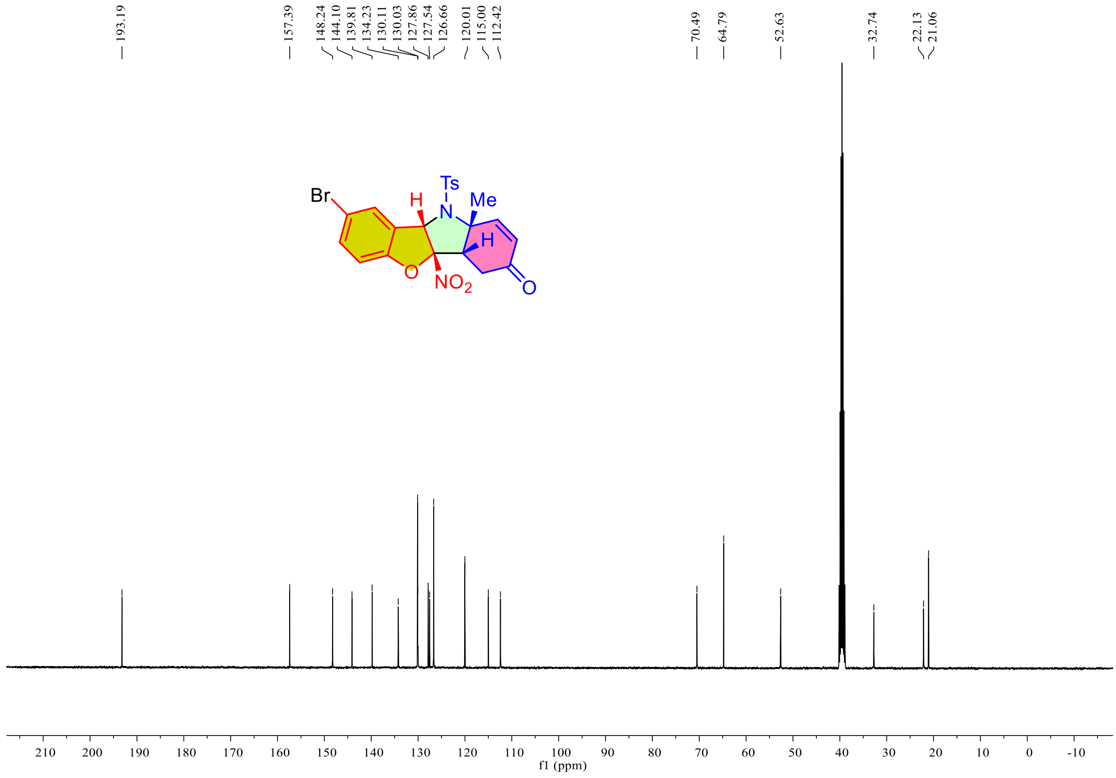

^{1}H NMR (400 MHz, $CDCl_3$) of **3ai**

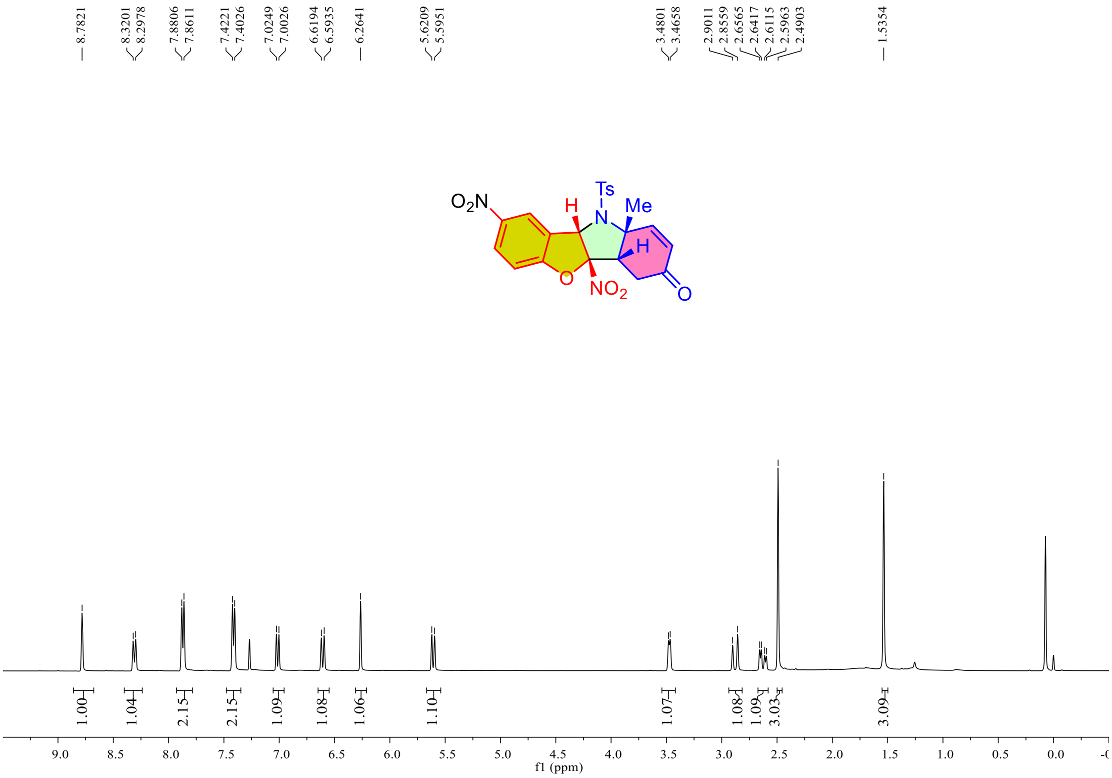

^{13}C{^{1}H} NMR (101 MHz, $CDCl_3$) of **3ai**

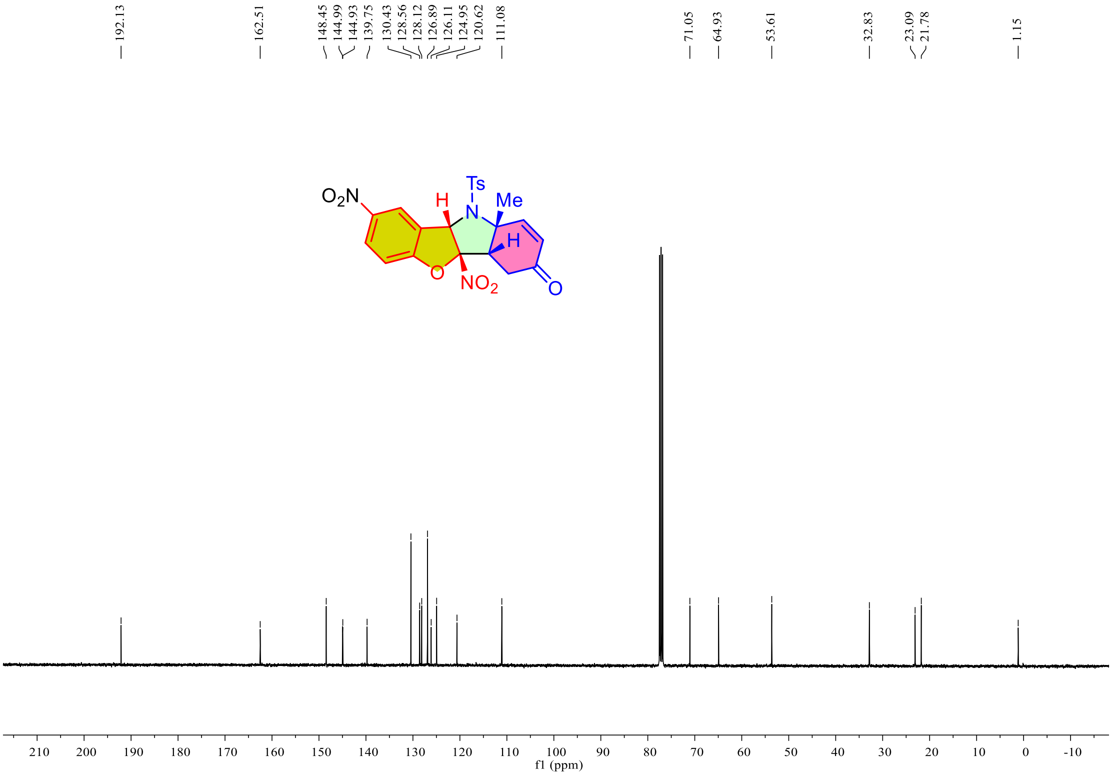

^{1}H NMR (400 MHz, DMSO-d_6) of **3aj**

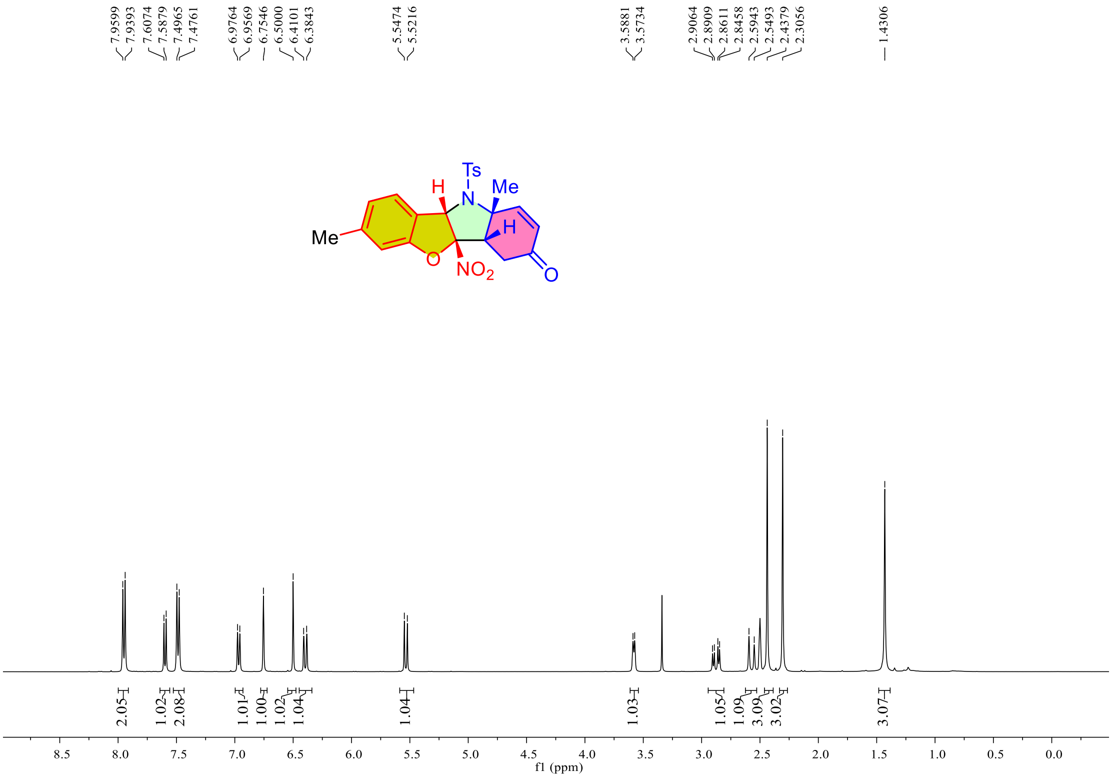

^{13}C{^{1}H} NMR (101 MHz, DMSO-d_6) of **3aj**

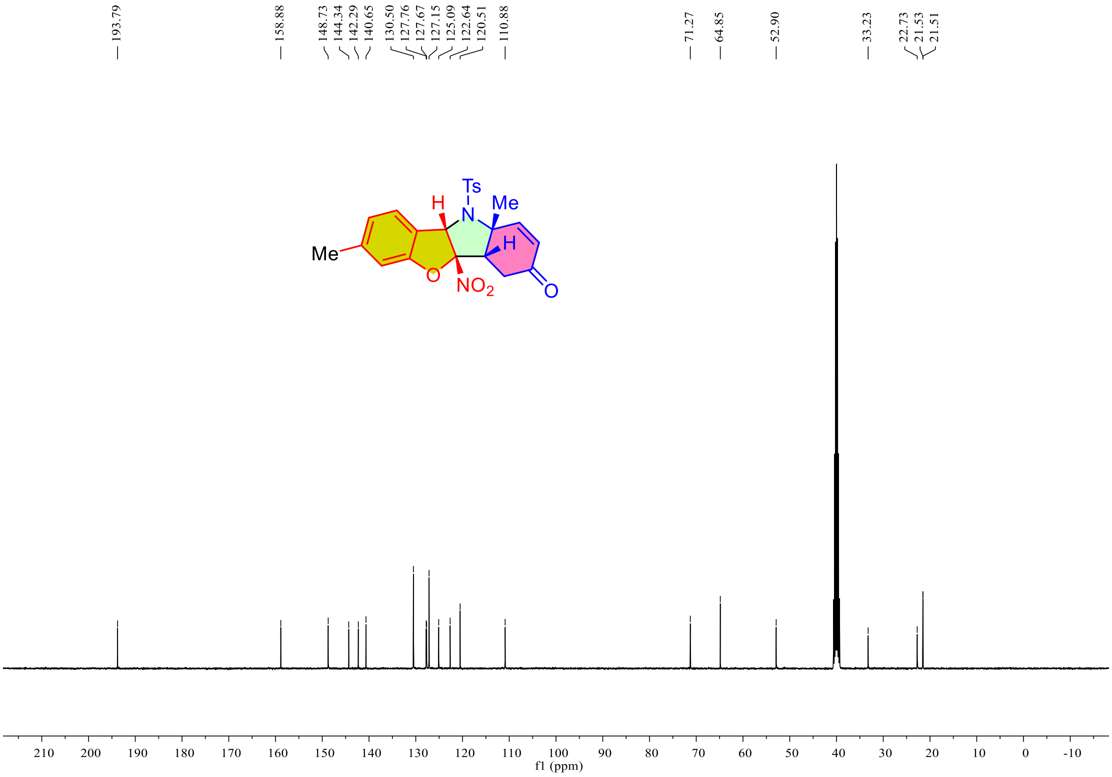

^{1}H NMR (400 MHz, DMSO-d_6) of **3ak**

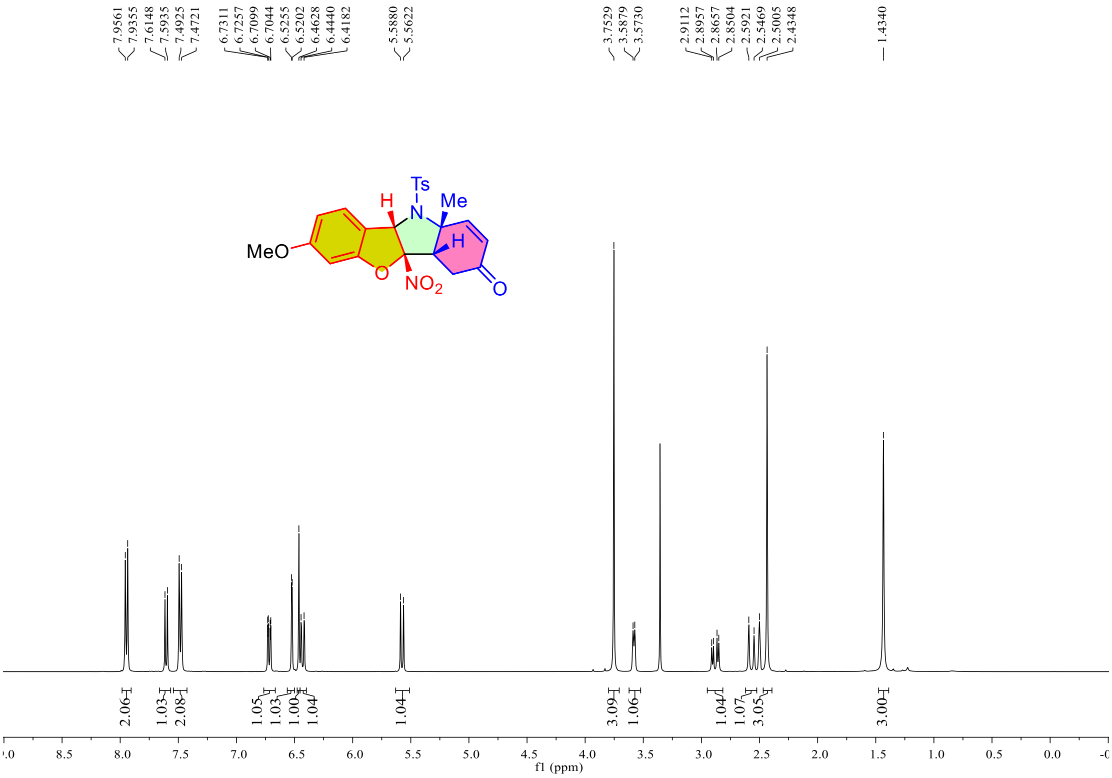

$^{13}C\{^{1}H\}$ NMR (101 MHz, DMSO-d_6) of **3ak**

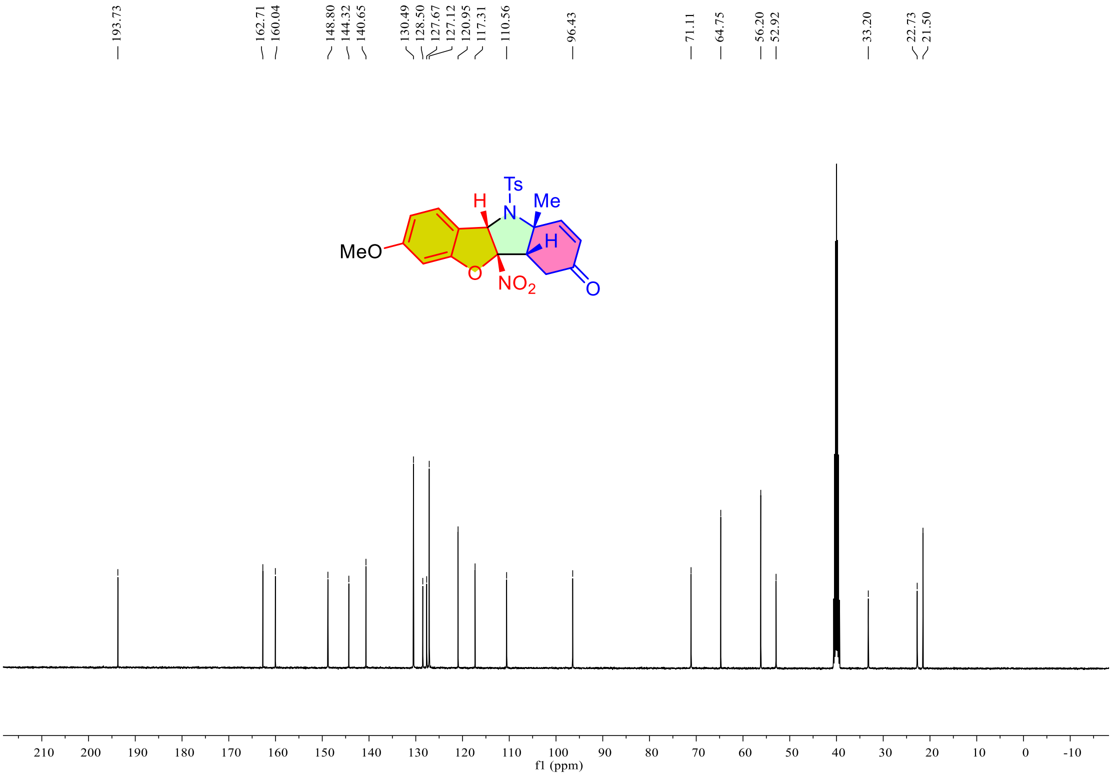

^{1}H NMR (400 MHz, DMSO-d_6) of **3al**

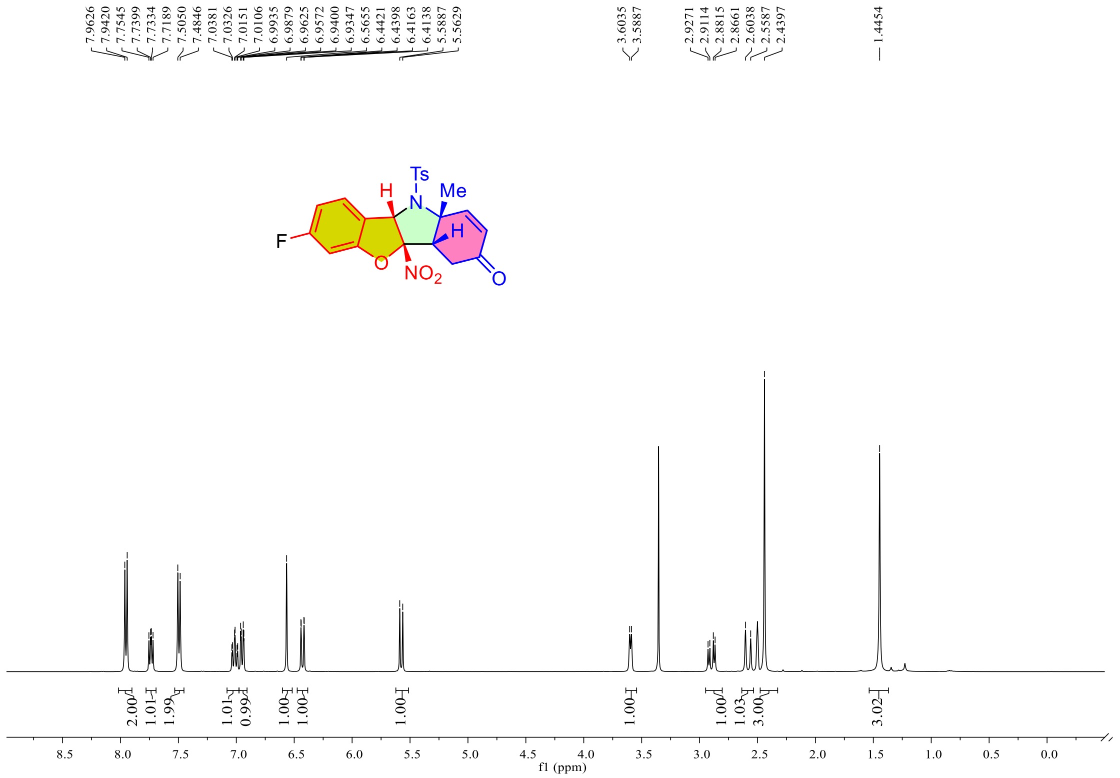

$^{13}C\{^{1}H\}$ NMR (101 MHz, DMSO-d_6) of **3al**

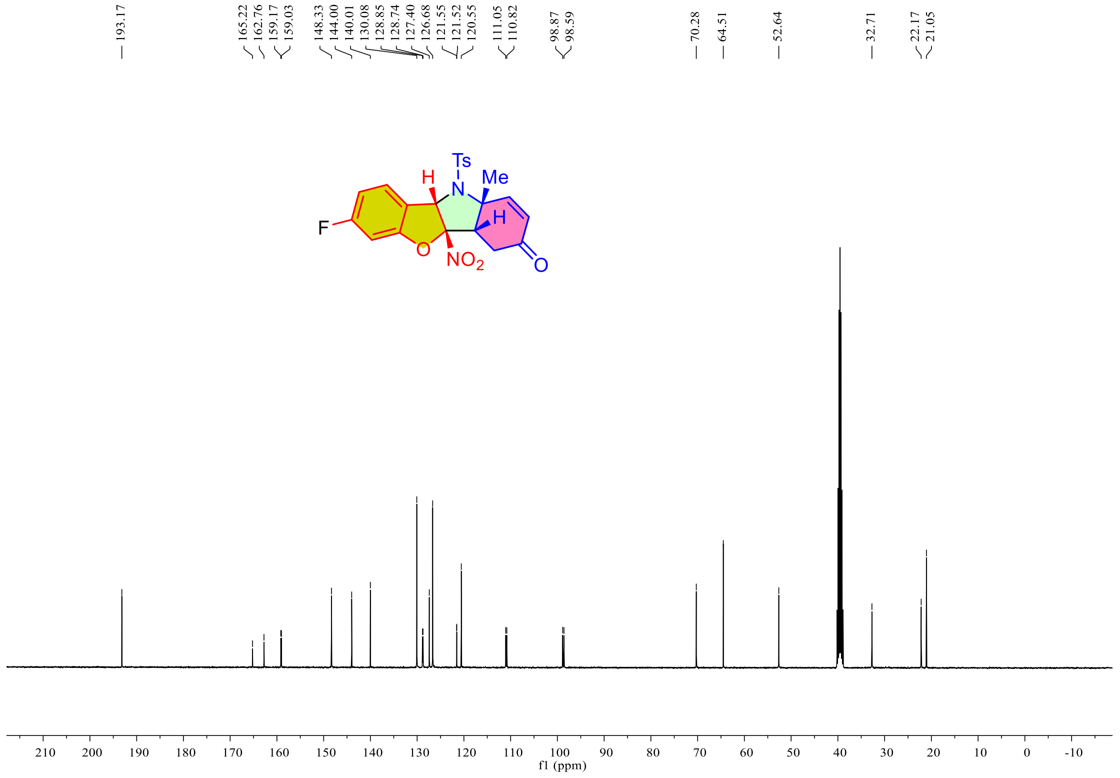

^{1}H NMR (400 MHz, DMSO-d_6) of **3am**

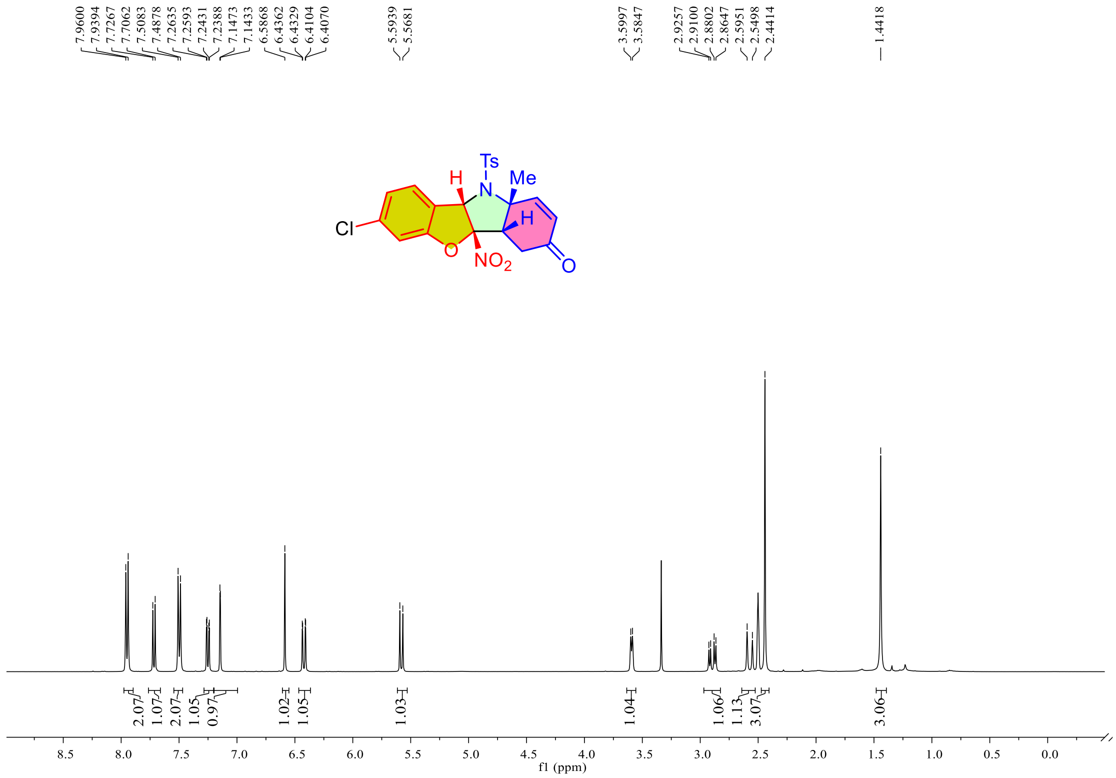

$^{13}C\{^{1}H\}$ NMR (101 MHz, DMSO-d_6) of **3am**

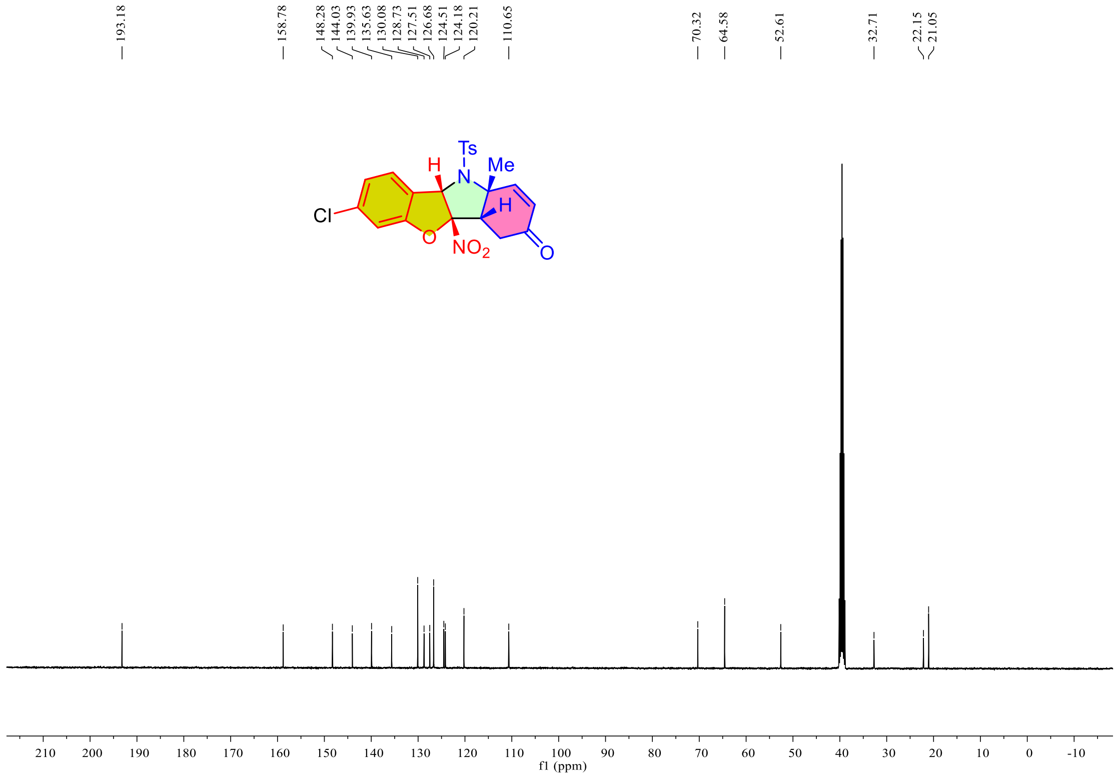

^{1}H NMR (400 MHz, DMSO-d_6) of **3an**

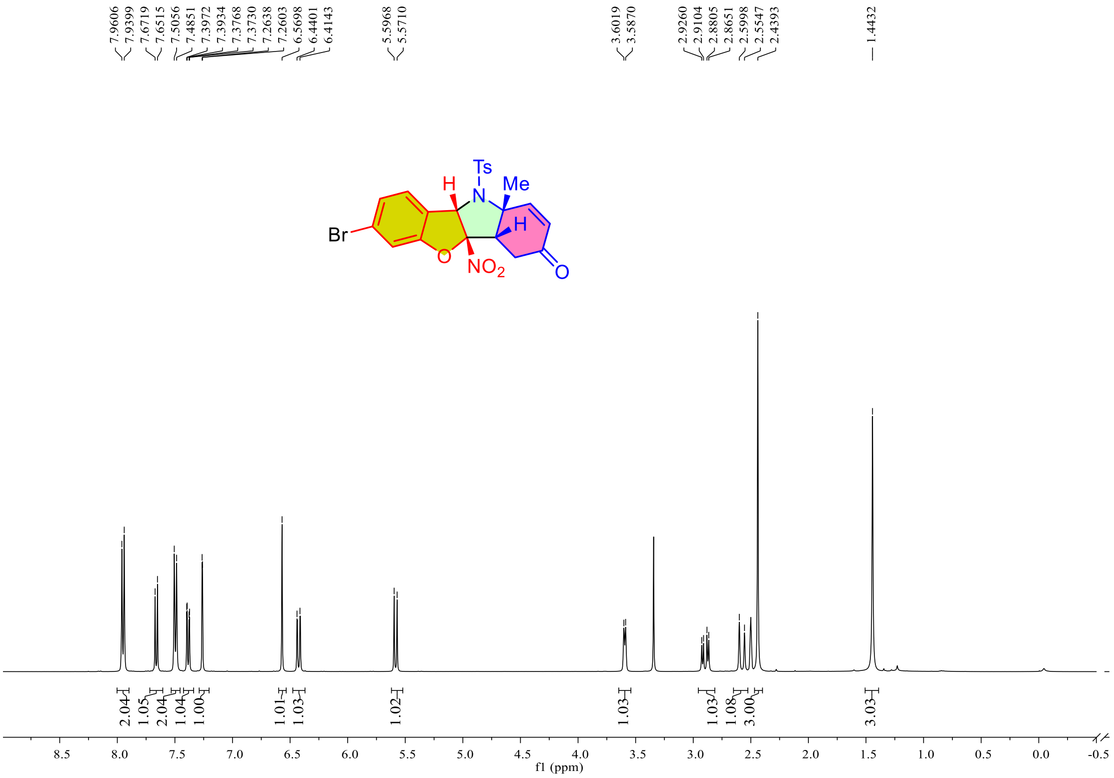

^{13}C{^{1}H} NMR (101 MHz, DMSO-d_6) of **3an**

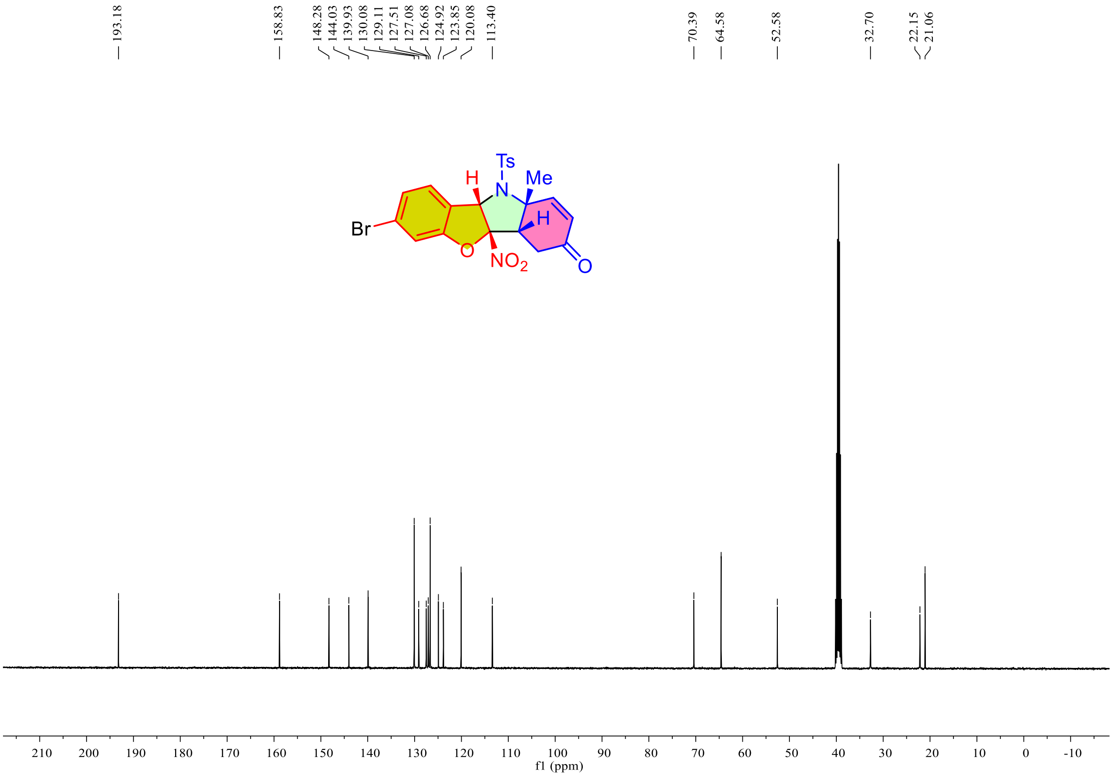

^{1}H NMR (400 MHz, DMSO-d_6) of **3ao**

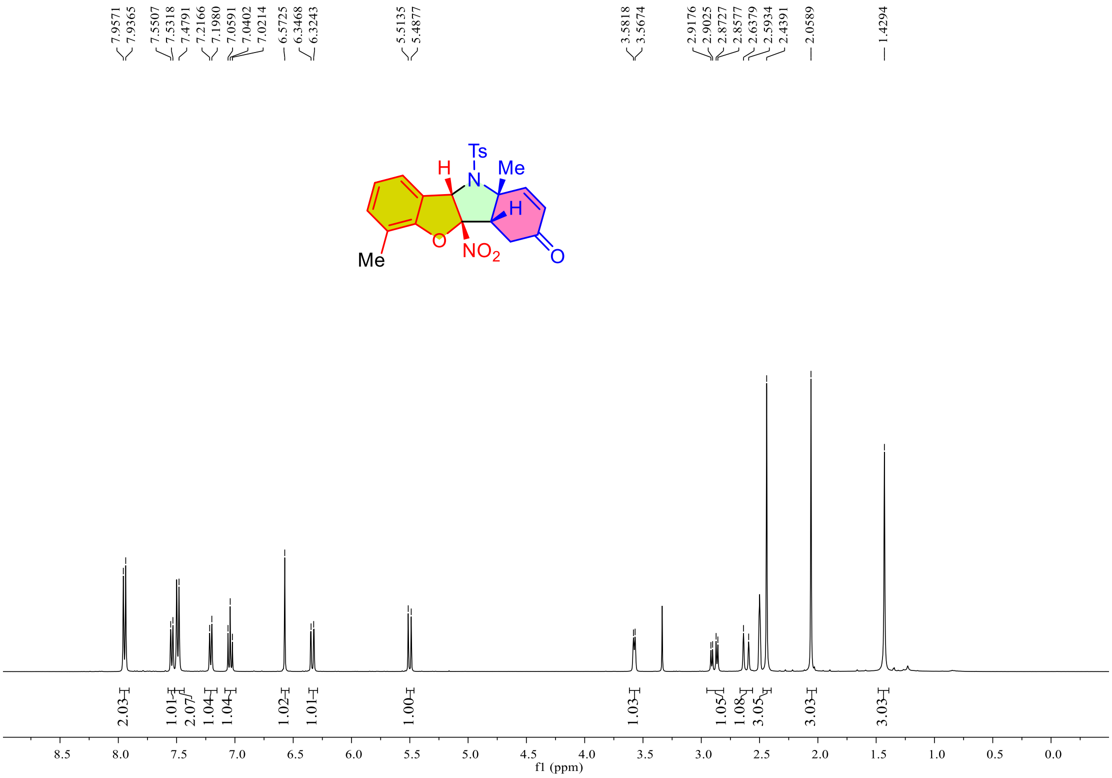

$^{13}C\{^{1}H\}$ NMR (101 MHz, DMSO-d_6) of **3ao**

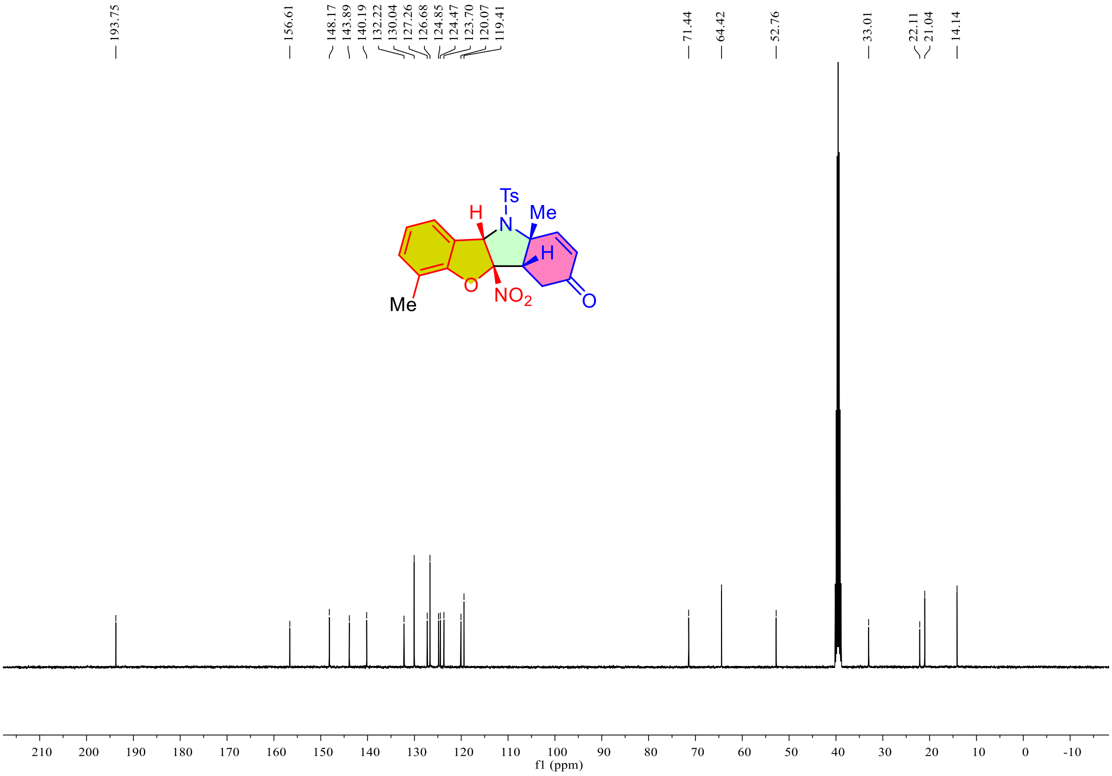

^1H NMR (400 MHz, $CDCl_3$) of **3ap**

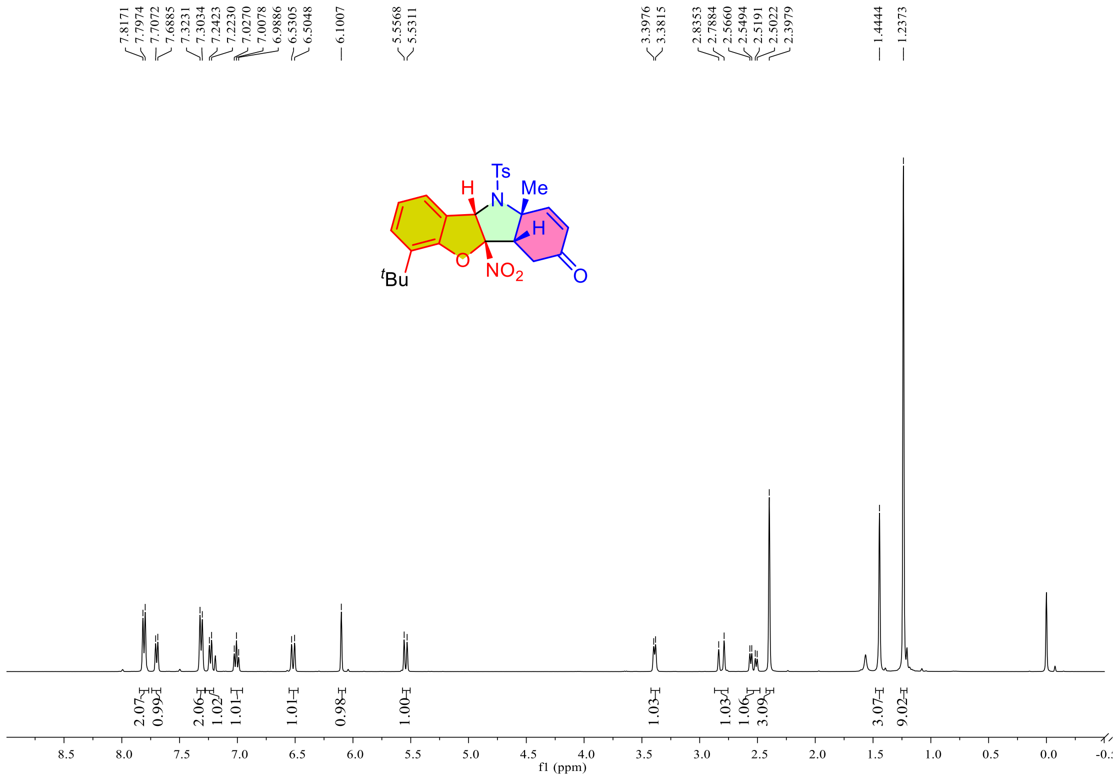

$^{13}C\{^1H\}$ NMR (101 MHz, $CDCl_3$) of **3ap**

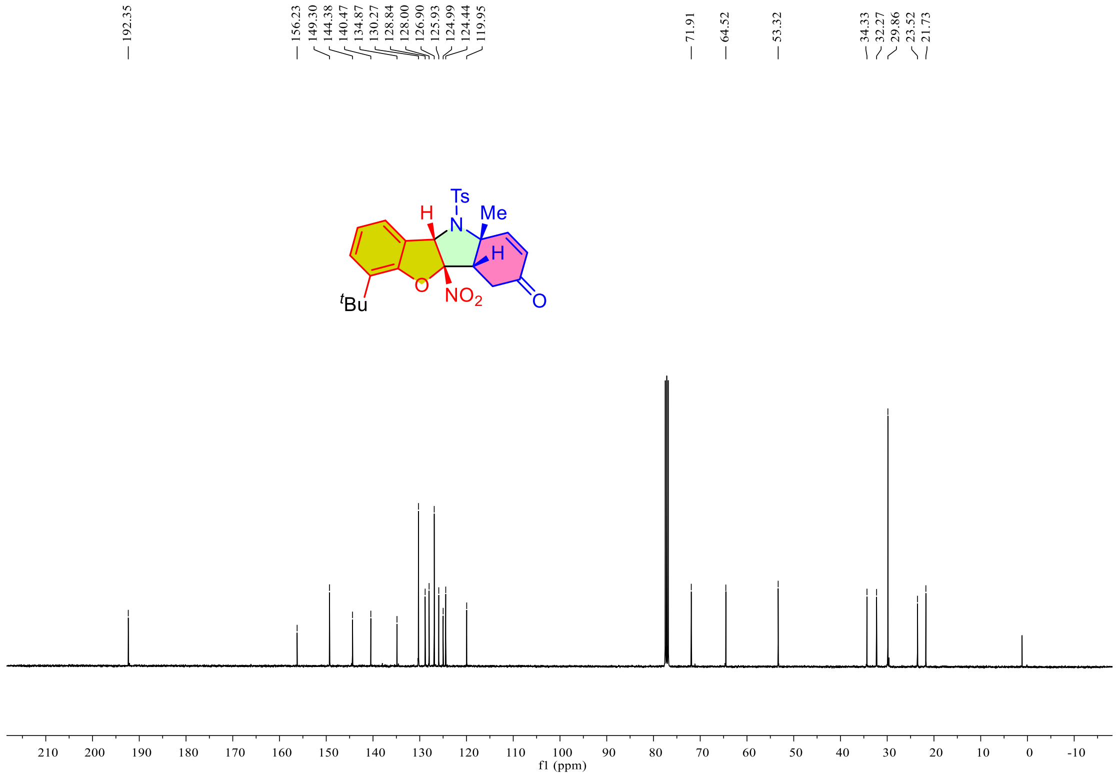

^{1}H NMR (400 MHz, DMSO-d_6) of **3aq**

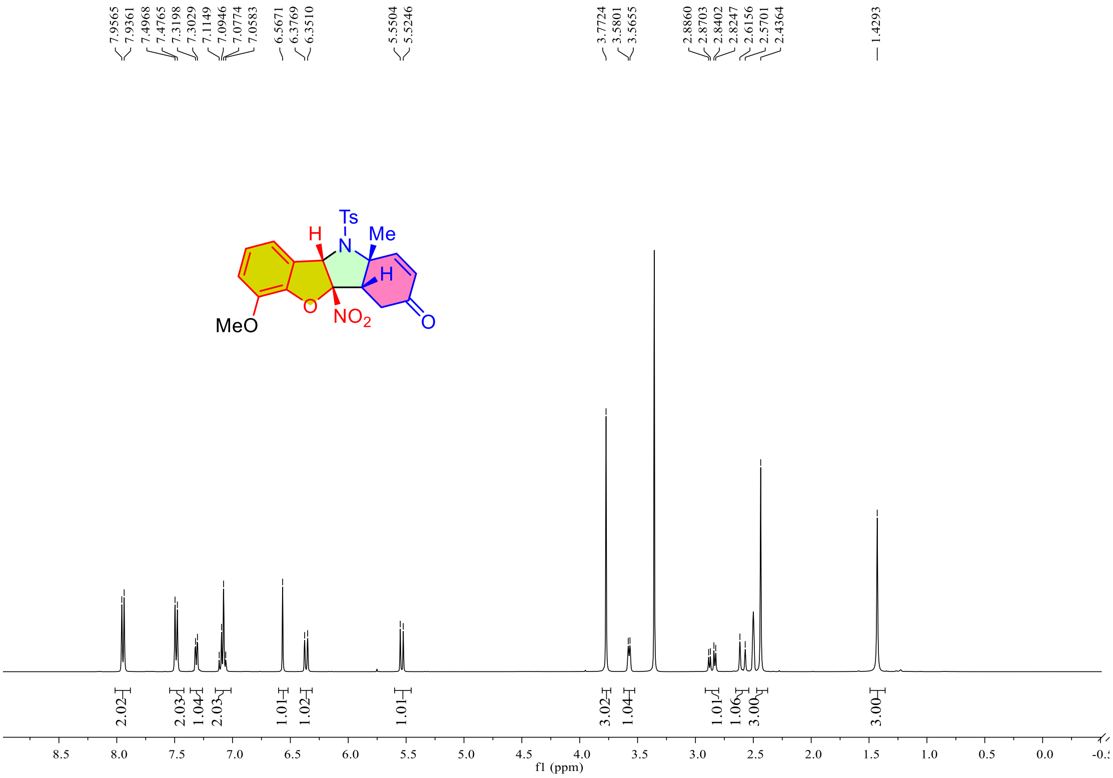

^{13}C{^{1}H} NMR (101 MHz, DMSO-d_6) of **3aq**

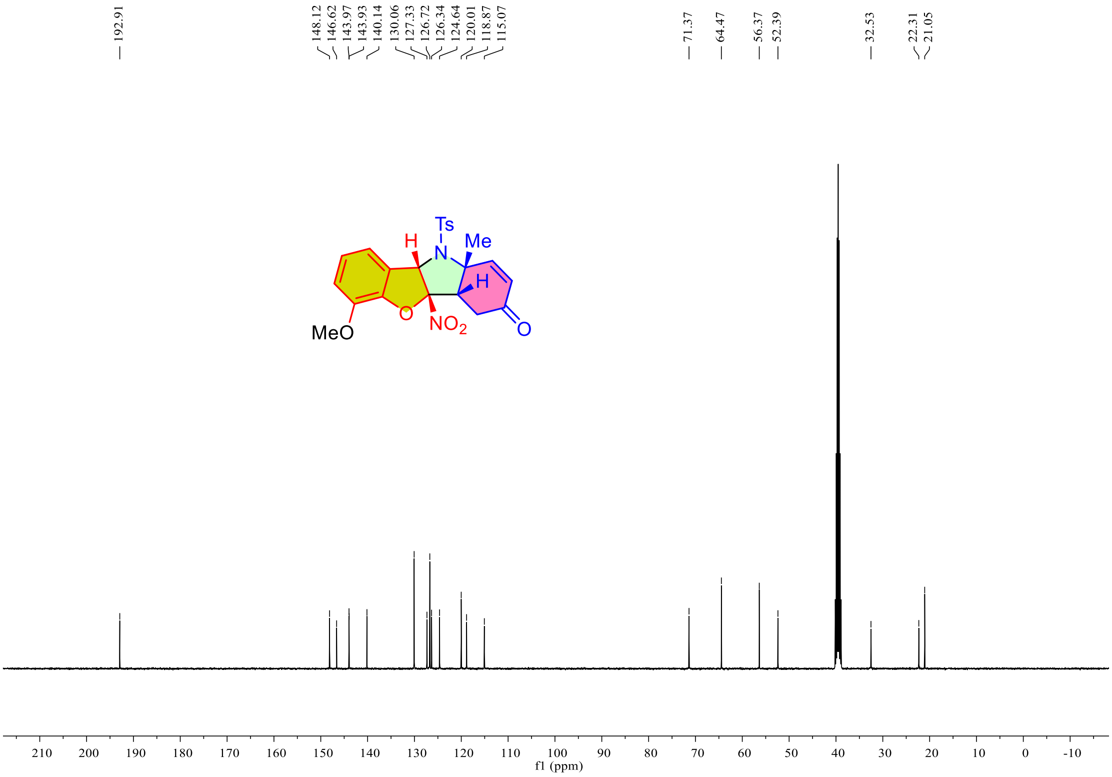

^{1}H NMR (400 MHz, DMSO-d_6) of **3ar**

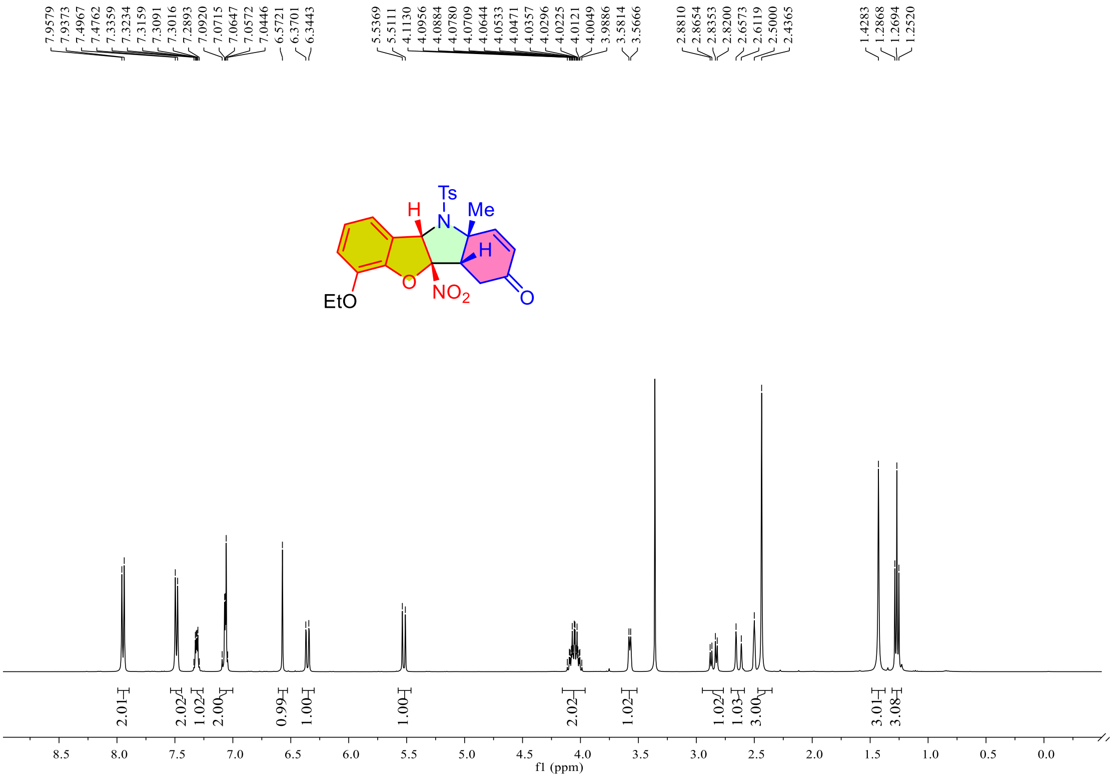

$^{13}C\{^{1}H\}$ NMR (101 MHz, DMSO-d_6) of **3ar**

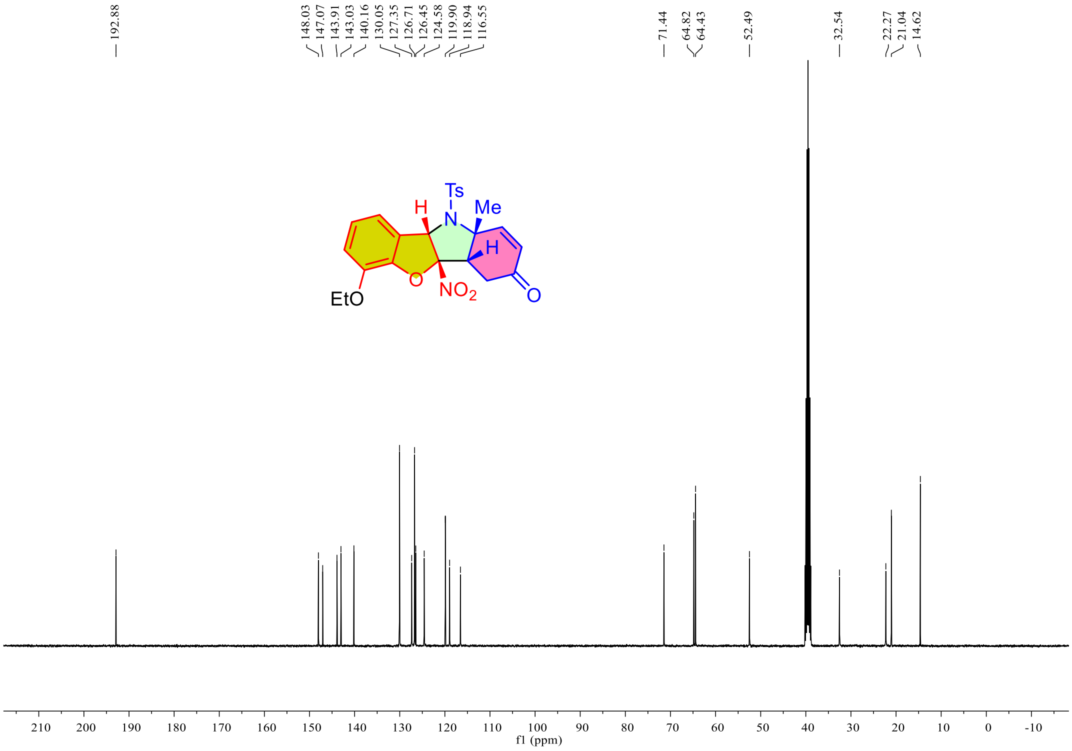

^{1}H NMR (400 MHz, DMSO-d_6) of **3as**

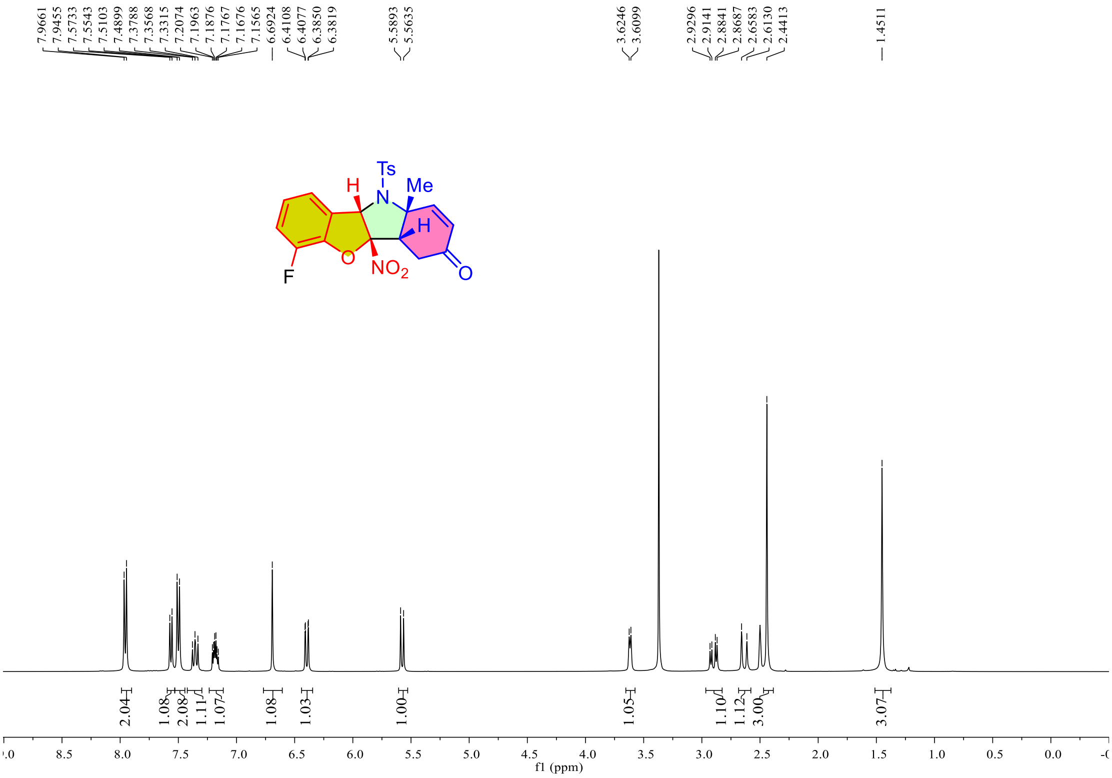

^{13}C{^{1}H} NMR (101 MHz, DMSO-d_6) of **3as**

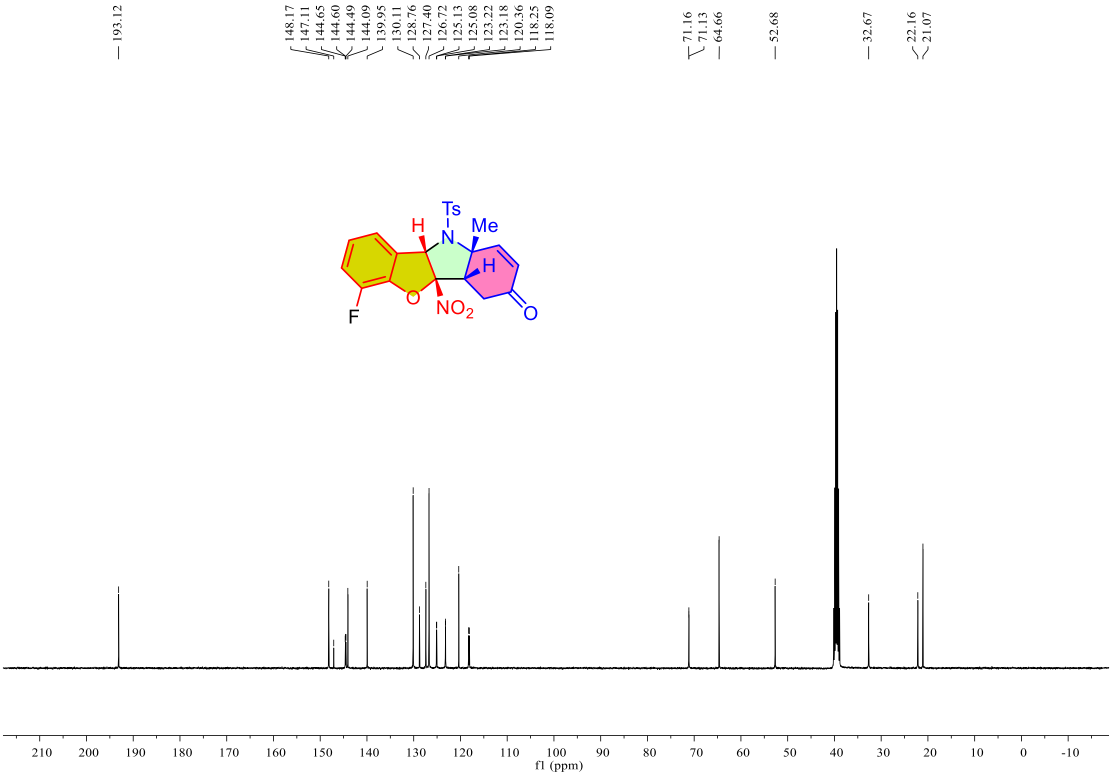

^{1}H NMR (400 MHz, DMSO-d_6) of **3at**

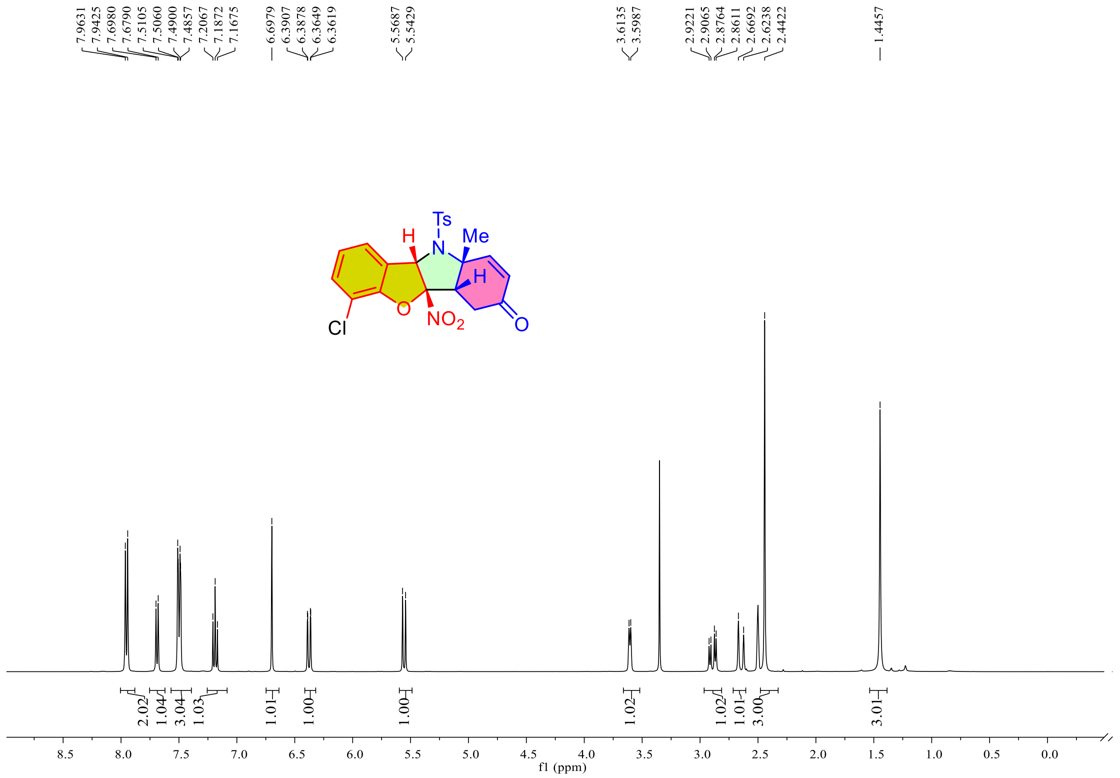

^{13}C{^{1}H} NMR (101 MHz, DMSO-d_6) of **3at**

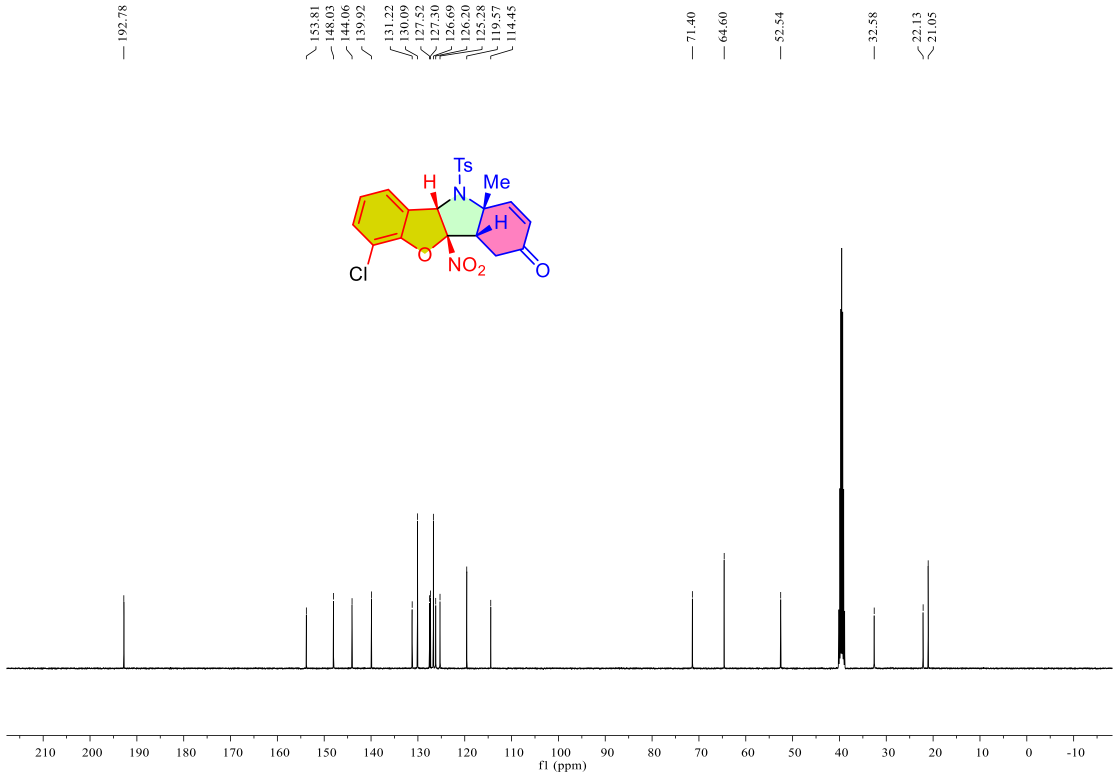

^{1}H NMR (400 MHz, DMSO-d_6) of **3au**

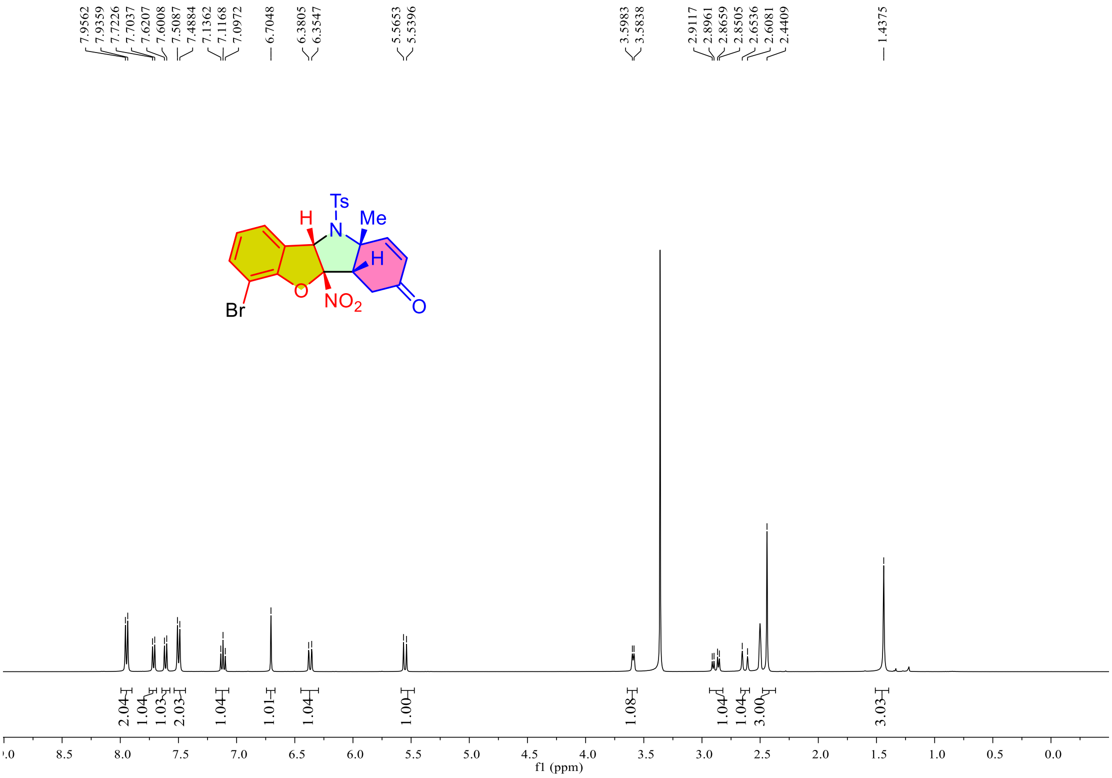

$^{13}C\{^{1}H\}$ NMR (101 MHz, DMSO-d_6) of **3au**

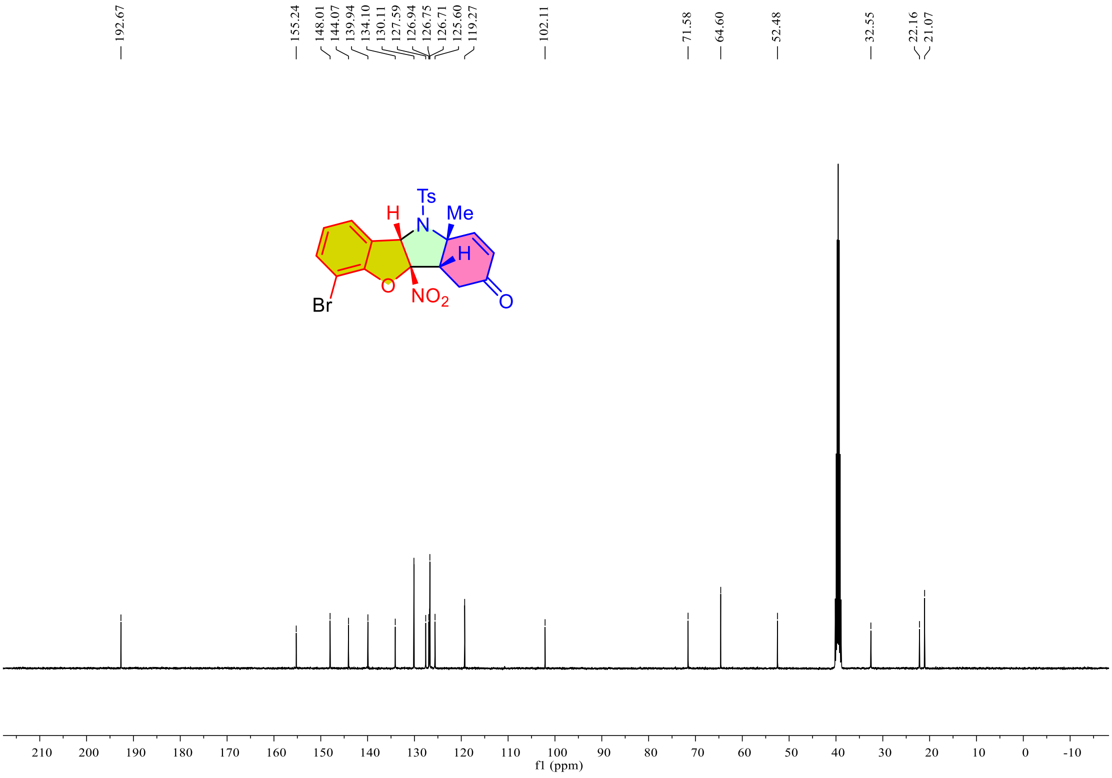

^{1}H NMR (400 MHz, $CDCl_3$) of **3av**

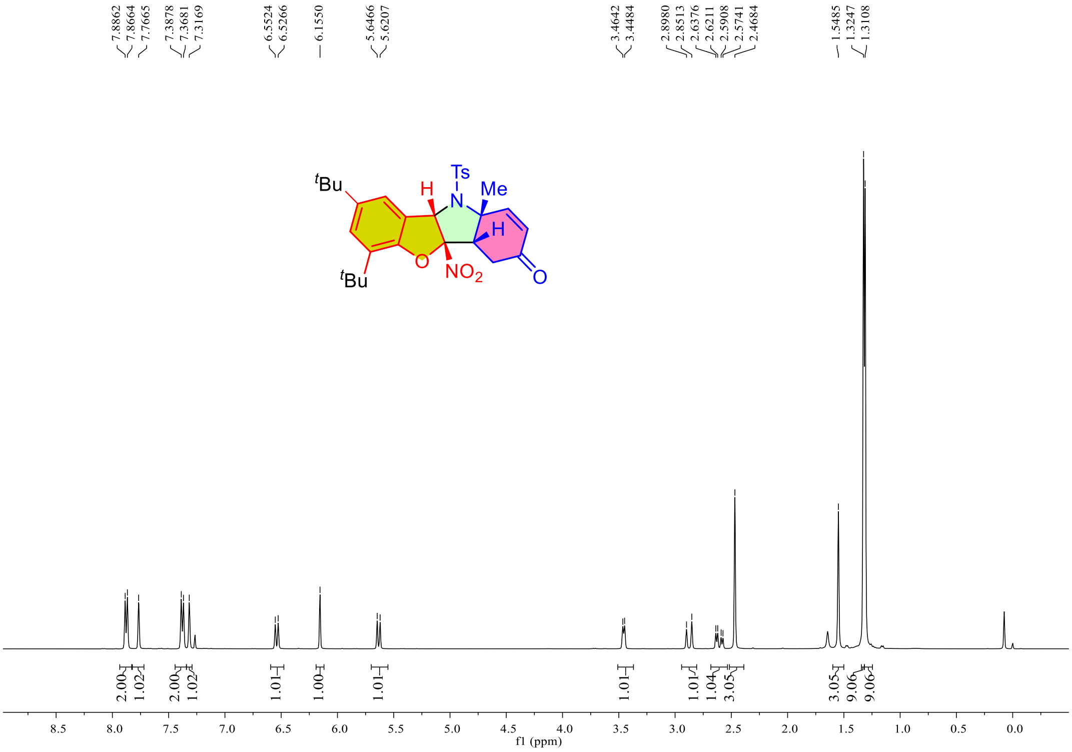

$^{13}C\{^{1}H\}$ NMR (101 MHz, $CDCl_3$) of **3av**

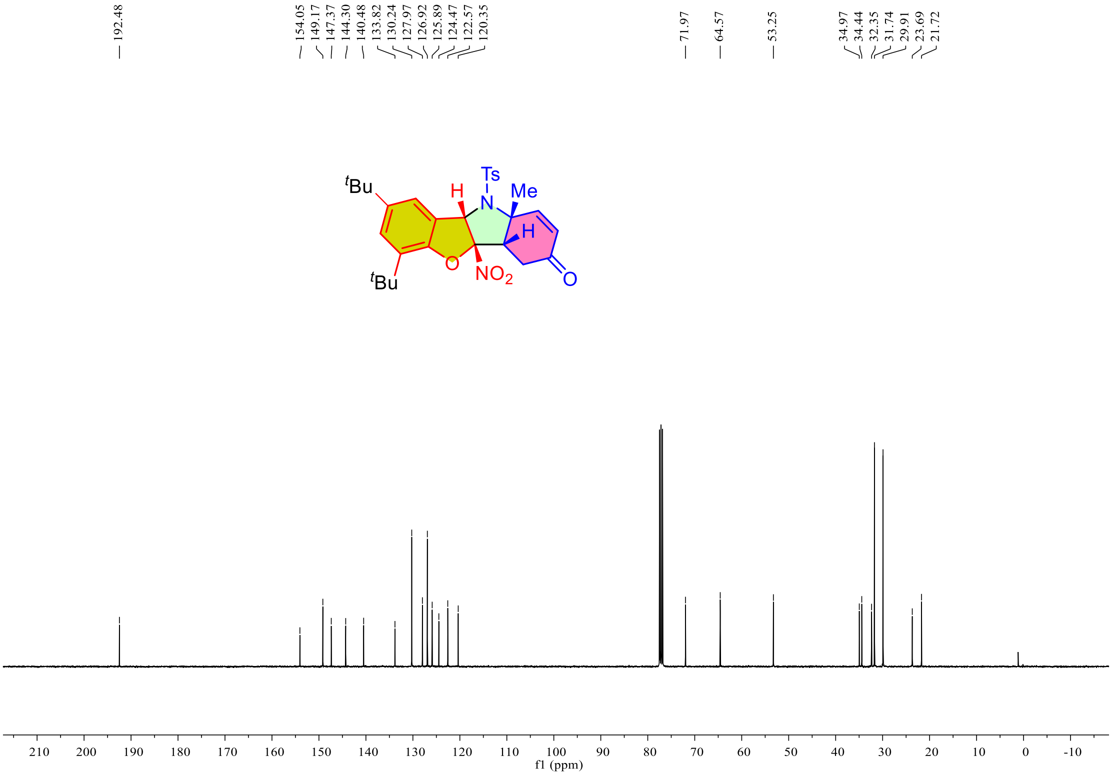

^{1}H NMR (400 MHz, $CDCl_3$) of **3aw**

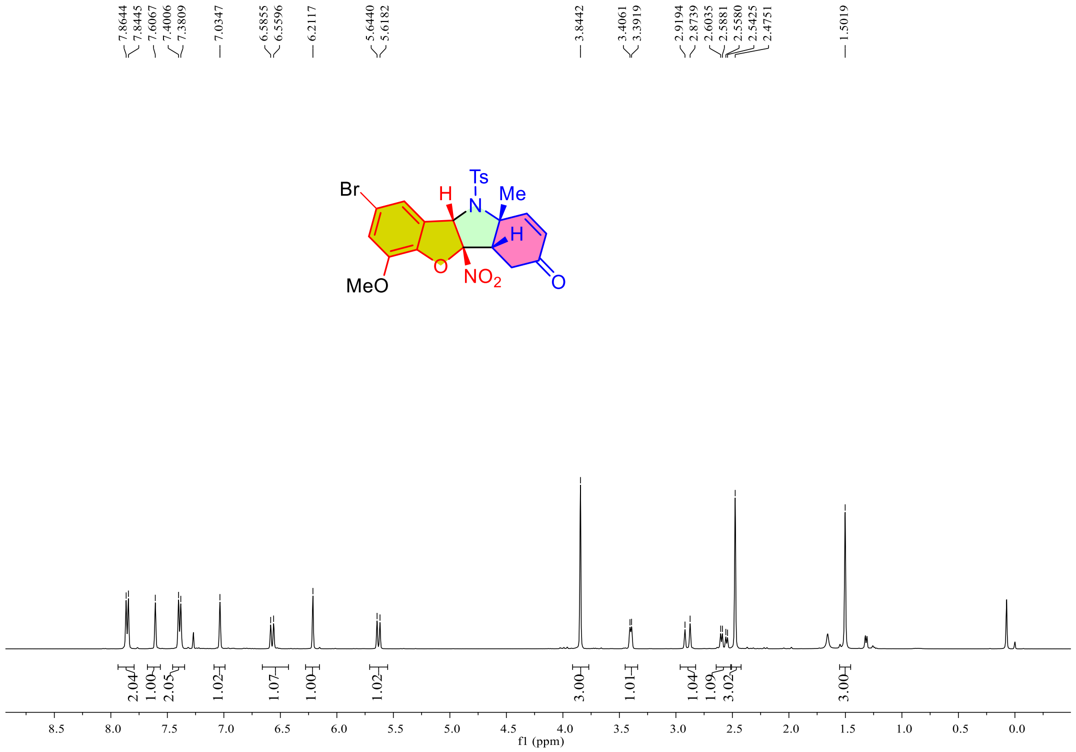

^{13}C{^{1}H} NMR (101 MHz, $CDCl_3$) of **3aw**

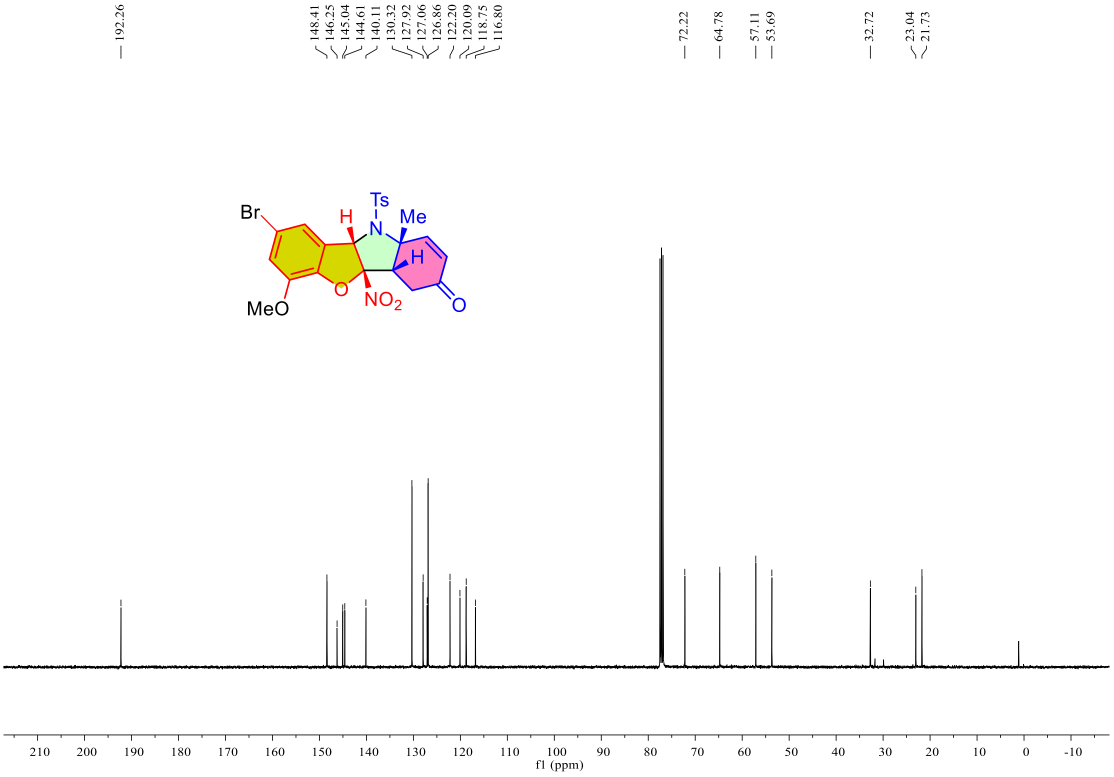

^{1}H NMR (400 MHz, DMSO-d_6) of **3ax**

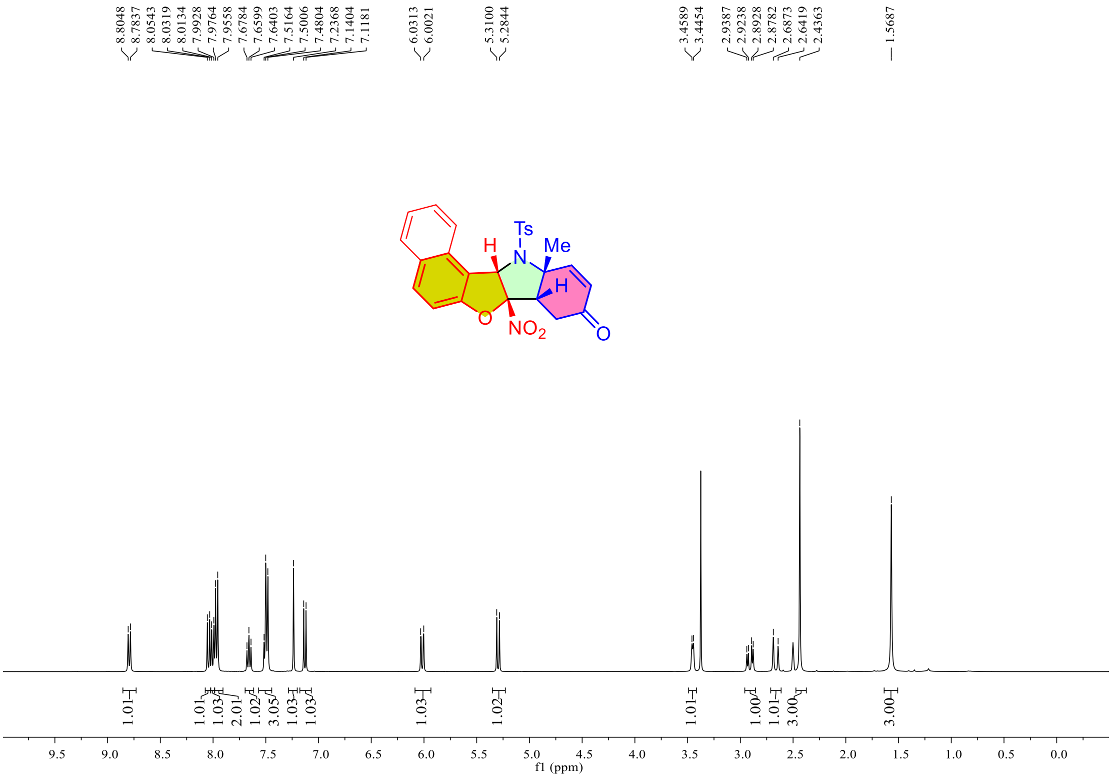

$^{13}C\{^{1}H\}$ NMR (101 MHz, DMSO-d_6) of **3ax**

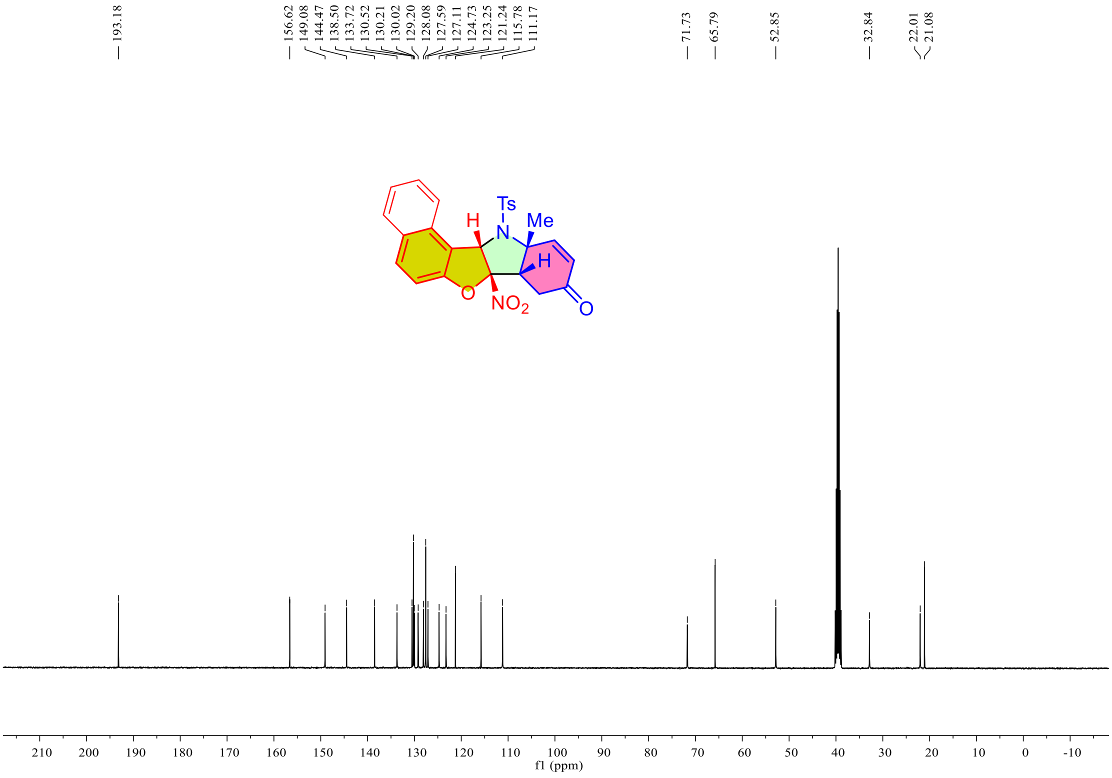

^{1}H NMR (400 MHz, $CDCl_3$) of **3ay**

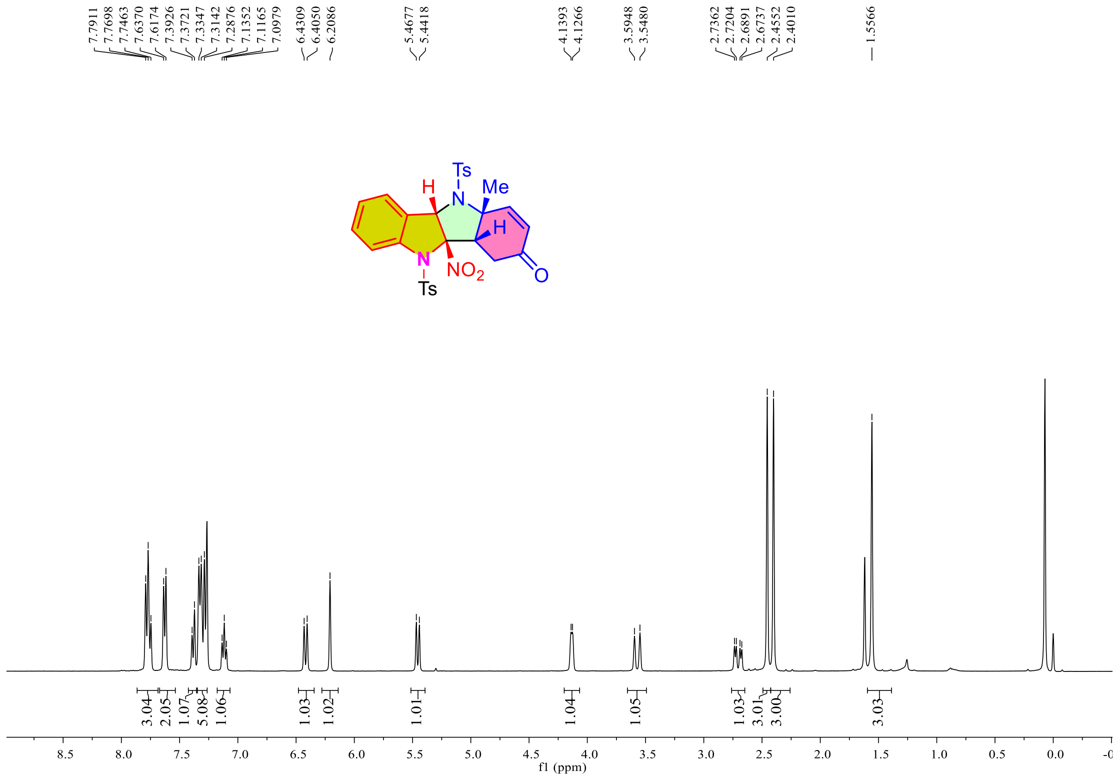

$^{13}C\{^1H\}$ NMR (101 MHz, $CDCl_3$) of **3ay**

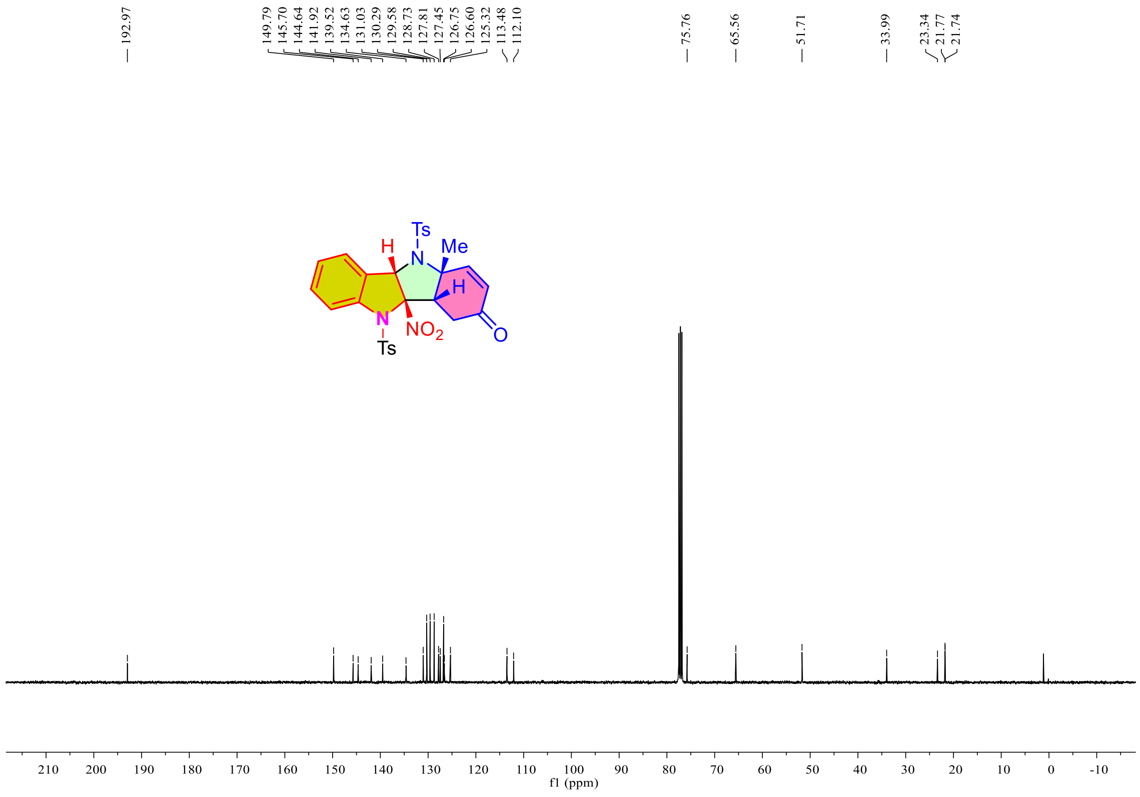

^{1}H NMR (400 MHz, $CDCl_3$) of **4**

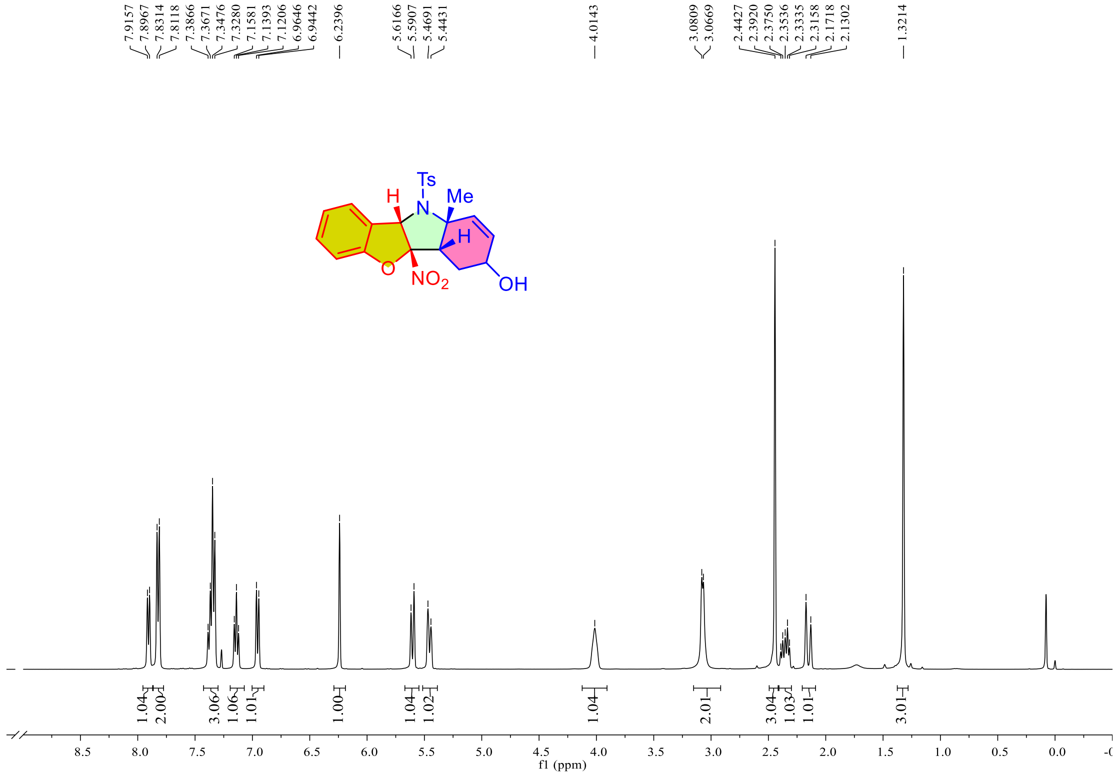

$^{13}C\{^{1}H\}$ NMR (101 MHz, $CDCl_3$) of **4**

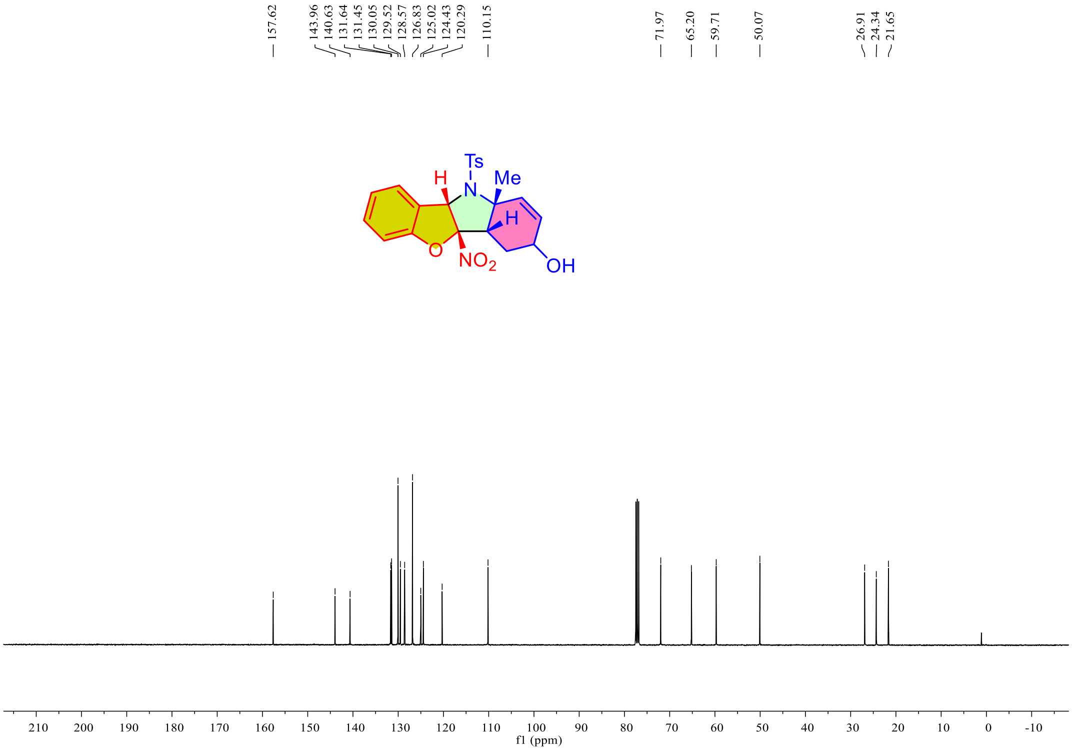

^{1}H NMR (400 MHz, $CDCl_3$) of **5**

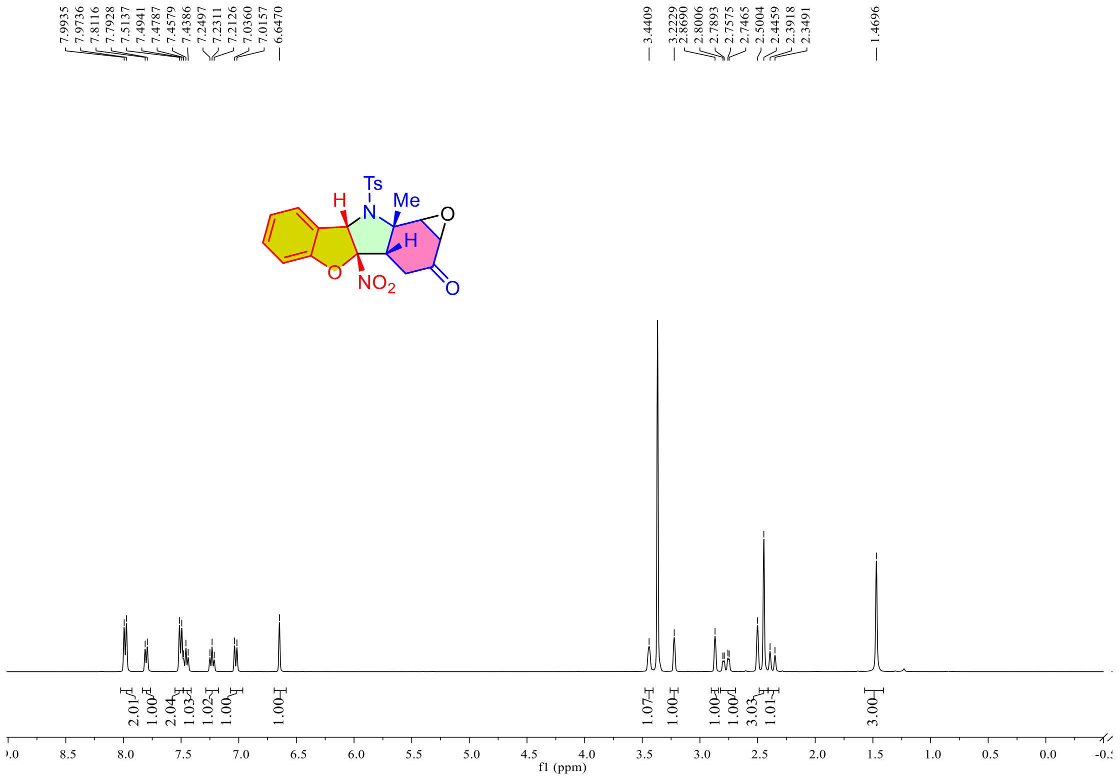

^{13}C{^{1}H} NMR (101 MHz, $CDCl_3$) of **5**

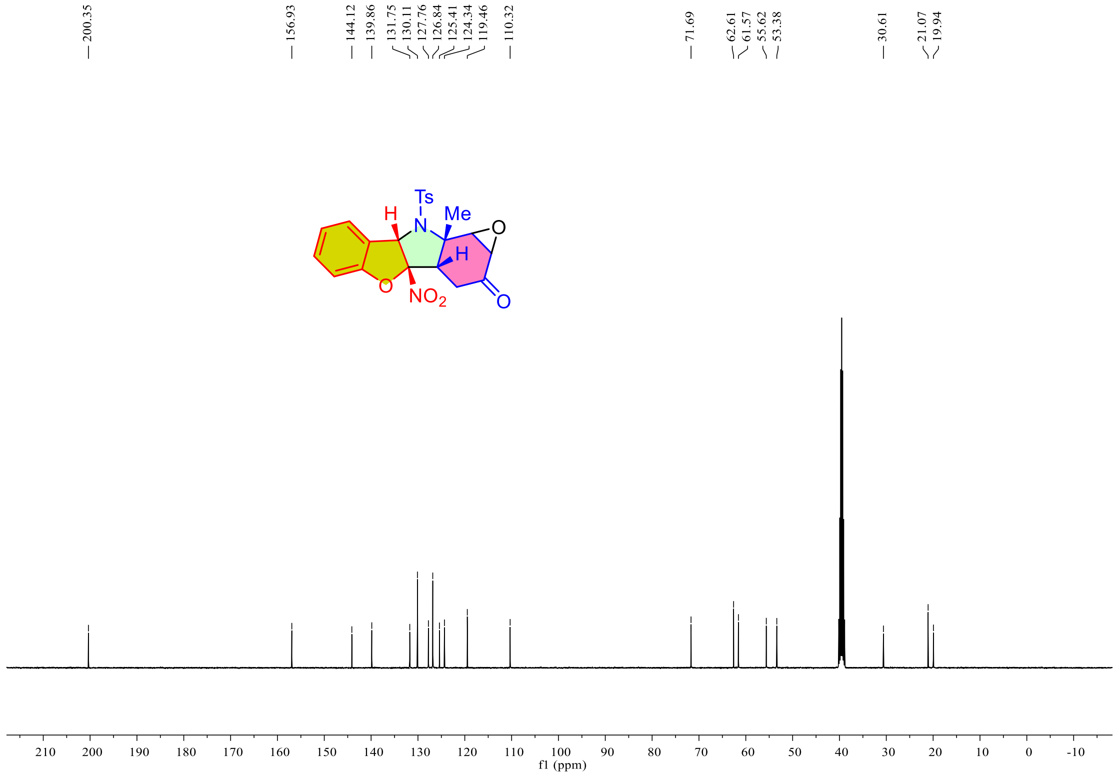

^{1}H NMR (400 MHz, $CDCl_3$) of **6**

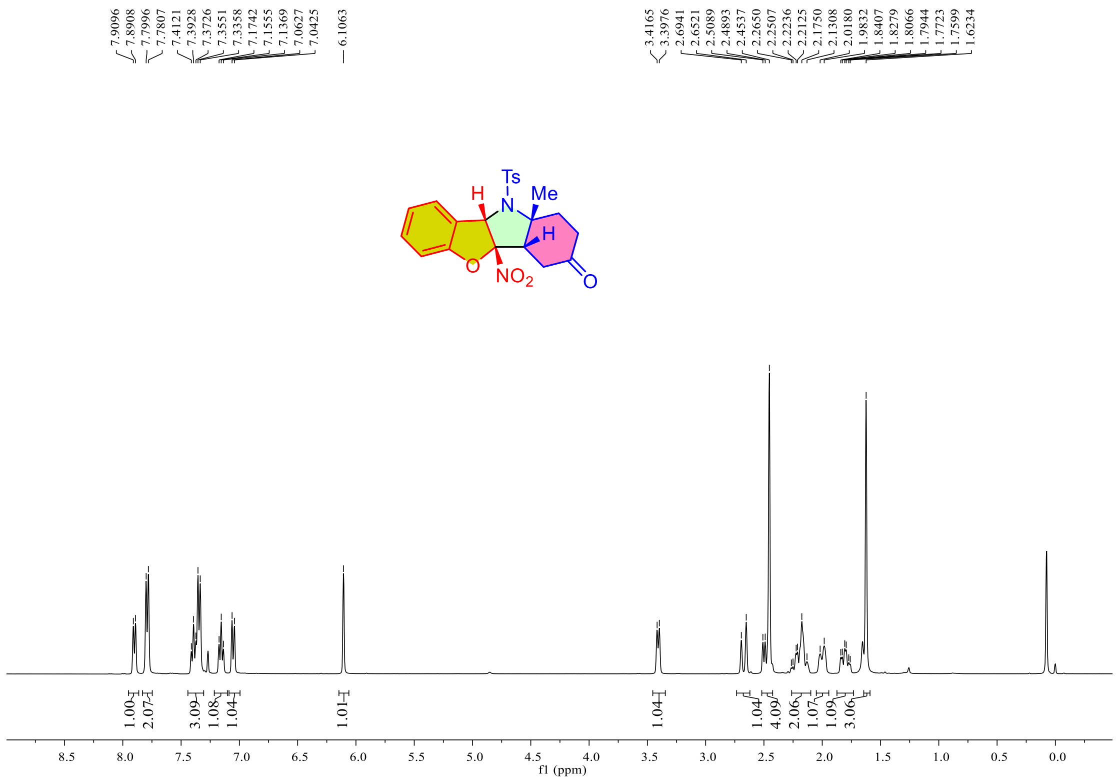

$^{13}C\{^{1}H\}$ NMR (101 MHz, $CDCl_3$) of **6**

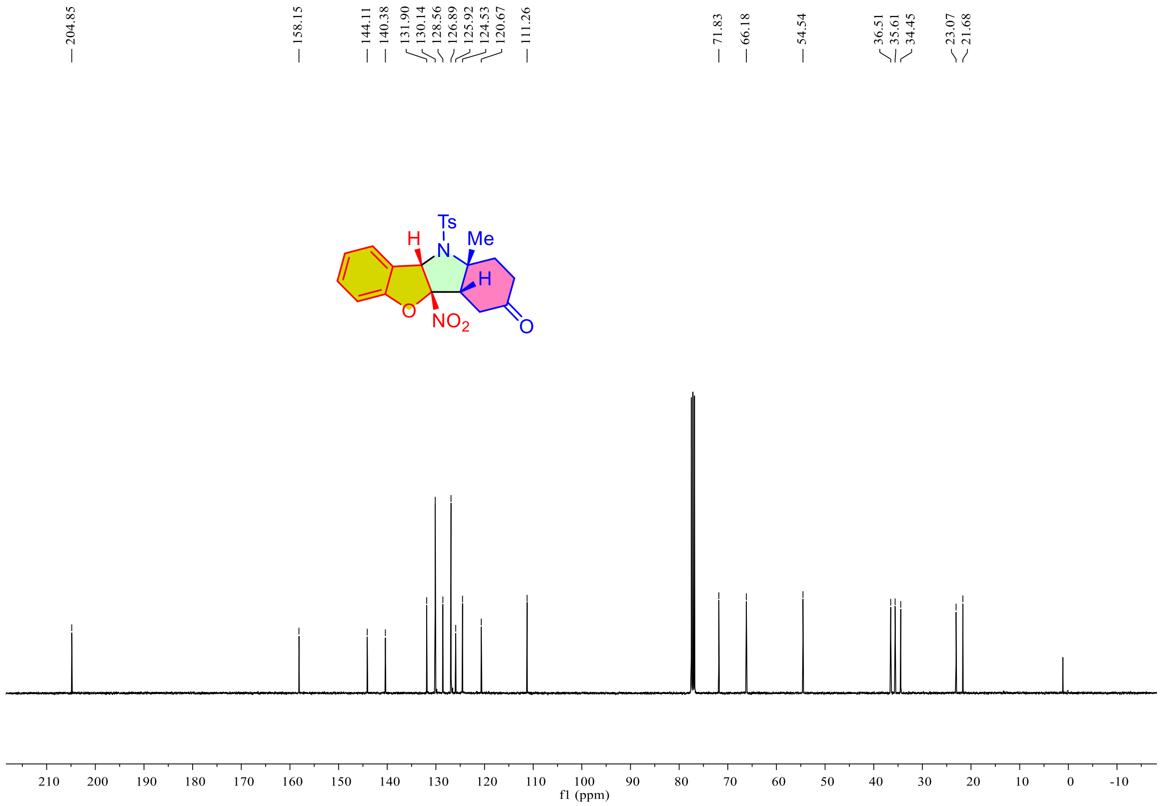

Supplement: Supplementary file 1 [file molecules-29-01163-s001.zip › Supporting Information.pdf]
